# Global Analysis of Alternative Splicing Difference in Peripheral Immune Organs between Tongcheng Pigs and Large White Pigs Artificially Infected with PRRSV *In Vivo*

**DOI:** 10.1155/2020/4045204

**Published:** 2020-01-30

**Authors:** Yu Zhang, Liyao Xue, Hang Xu, Wan Liang, Qingqing Wu, Qingde Zhang, Xiang Zhou, Bang Liu

**Affiliations:** ^1^Key Laboratory of Agricultural Animal Genetics, Breeding, and Reproduction of Ministry of Education, College of Animal Science and Technology, Huazhong Agricultural University, Wuhan 430070, Hubei, China; ^2^Laboratory Animal Centre, College of Veterinary Medicine, Huazhong Agricultural University, Wuhan 430070, Hubei, China

## Abstract

Alternative splicing (AS) plays a significant role in regulating gene expression at the transcriptional level in eukaryotes. Flexibility and diversity of transcriptome and proteome can be significantly increased through alternative splicing of genes. In the present study, transcriptome data of peripheral immune organs including spleen and inguinal lymph nodes (ILN) were used to identify AS difference between PRRSV-resistant Tongcheng (TC) pigs and PRRSV-susceptible Large White (LW) pigs artificially infected with porcine reproductive and respiratory syndrome virus (PRRSV) *in vivo*. The results showed that PRRSV infection induced global alternative splicing events (ASEs) with different modes. Among them, 373 genes and 595 genes in the spleen and ILN of TC pigs, while 458 genes and 560 genes in the spleen and ILN of LW pigs had significantly differential ASEs. Alternative splicing was subject to tissue-specific and lineage-specific regulation in response to PRRSV infection. Enriched GO terms and pathways showed that genes with differential ASEs played important roles in transcriptional regulation, immune response, metabolism, and apoptosis. Furthermore, a splicing factor associated with apoptosis, *SRSF4*, was significantly upregulated in LW pigs. Functional analysis on apoptosis associated genes was validated by RT-PCR and DNA sequencing. These findings revealed different response to PRRSV between PRRSV-resistant TC pigs and PRRSV-susceptible LW pigs at the level of alternative splicing, suggesting the potential relationship between AS and disease resistance to PRRSV.

## 1. Introduction

Porcine reproductive and respiratory syndrome (PRRS) has devastated the swine industries in Africa, Asia, Europe, and North America for many years. Since 2006, highly pathogenic PRRS (HP-PRRS) has emerged in China and then spread rapidly to other Asian countries [1, 2]. No doubt, PRRS presents a major health challenge and economic burdens for the swine industry worldwide [3]. The major clinical symptoms of PRRS include severe respiratory diseases in pigs of any age and reproductive disorder in sows [1, 4]. The respiratory symptom is characterized by fever, interstitial pneumonia, and severe lesions in lung and lymph nodes, which usually cause high mortality in nursery pigs. The reproductive disorder is typically shown increased rates of abortions, mummies, and stillbirth in sows [1, 4]. The pathogen of PRRS is called the PRRS virus (PRRSV), which is a small positive strand RNA virus belonging to *Arterivirus* family. Currently, there are two main PRRSV genotypes based on geographic origins: type I (European) and type II (North American) PRRSV [5]. Type I and type II PRRSV only share approximate 55–70% nucleotide identity and 50–80% amino acid identity of viral genes [6]. The target cells of PRRSV are monocytes and macrophages in different tissues, and the fully differentiated porcine alveolar macrophages (PAMs) are the major target cell [7, 8].

Vaccination is unsuccessful in preventing PRRSV infection due to its rapid evolution and hundreds of new strains emergence with a high mutation rate and recombination rate [9]. As a result, it has been suggested that genetic improvement by enhancing host resistance to PRRSV is a feasible strategy to control PRRS [10]. In order to investigate potential genes or pathways associated with host resistance to PRRSV or pathogenesis of PRRSV, studies have explored whole genome transcriptome analysis of PAMs, lungs or blood samples challenged with different virulent PRRSV strains using microarray, serial analysis of gene expression (SAGE) and RNA-seq [11–14]. Microarray data revealed critical differentially expressed genes in lung tissue between Dapulian (DPL) pigs and Duroc × Landrace × Yorkshire (DLY) pigs after PRRSV infection resulting in different clinical outcomes [14]. Transcriptome analysis of lung dendritic cells (DCs) derived from Pietrain and Duroc revealed Duroc reacted more distinctly and strongly than Pietrain during various periods of PRRSV infection [15]. The transcriptome study of PAMs in Tongcheng (TC) pigs and Large White (LW) pigs showed that two breeds had different immune responses to PRRSV infection [16]. It is mainly manifested that the differentially expressed genes in TC pigs are enriched in activation of leukocyte extravasation and suppression of apoptosis while LW pigs are enriched in the suppression of G*α*q and PI3K-AKT signaling [17]. Although those transcriptome studies have provided useful genes and pathways information related to the pathogenesis of PRRSV and host immune response, they have not investigated the complexity of transcriptome arising from RNA processing.

Alternative splicing (AS) is one of the most important posttranscriptional RNA processing, which can significantly change the sequence of RNA transcript [18]. AS plays a critical role in regulating gene expression at the transcriptional level in eukaryotes. Flexibility and diversity of transcriptome and proteome can be significantly increased through alternative splicing of genes [19, 20]. Alternative splicing events (ASEs) mainly include skipped exon (SE), retained intron (RI), an alternative to 5′ splicing site (AS5), an alternative to 3′ splicing site (AS3), and mutually exclusive exon (MXE) [21]. Virus-host interaction studies of a wide range of viruses have identified that AS can change many biological processes in host cells upon viral infection, including activation of host immune response, modulation of synthesis system, and cellular protein quality control [22–24]. Poliovirus protease 2A (2A^pro^) can impair RNA splicing to modulate host gene expression and avoid antiviral response [25]. Influenza A virus inhibits the cellular gene expression through interaction with the spliceosome complex by NS1 protein [26]. Moreover, the genetic variability of different individuals or breeds has been proven to affect alternative splicing outcomes [27, 28]. Therefore, the global profiling of alternative splicing differences among different breeds in response to PRRSV infection will provide us new insights of host resistance to PRRSV. In the present study, transcriptome data of peripheral immune organs including the spleen and inguinal lymph nodes (ILN) were used to identify AS difference between PRRSV-resistant TC pigs and PRRSV-susceptible LW pigs artificially infected with PRRSV *in vivo.* The results will enhance the understanding of the molecular mechanism of genetic resistance to PRRSV infection and provide detailed information for future studies.

## 2. Materials and Methods

### 2.1. Animals, Experimental Design, and Sample Collection

The PRRSV challenge experiments were conducted by our previous study [16]. Briefly, a total of twenty-four five-week-old pigs including twelve TC pigs and twelve LW pigs were selected to perform the artificial challenge experiment as described previously. All selected pigs are free of PRRSV, porcine circovirus (PCV), and pseudorabies virus (PRV). Six TC pigs and six LW pigs were intramuscularly challenged with PRRSV WuH3 strain at a viral dose of 10^5^ CCID50/mL (3 mL/15 kg), and the rest control pigs were challenged with the same amount of RPMI-1640 (Gibco, Grand Island, NY, USA). Then, all pigs were humanely euthanized for sample collection on day 7 after challenging. In this study, spleen and inguinal lymph nodes were collected for total RNA extraction and RNA sequencing library construction. All animal procedures were supervised and approved by the Ethical Committee for Animal Experiments at Huazhong Agricultural University (permit number: HZAUSW-2013-005).

### 2.2. RNA Preparation and Sequencing

Total RNA extraction was performed as described previously [17]. RNA degradation and contamination were monitored by electrophoresis. RNA purity, concentration, and integrity were measured by Agilent 2100 Bioanalyzer (Agilent, Santa Clara, California, USA). Total RNA of the spleen and ILN from twelve selected pigs (three pigs selected randomly from each group) were used for RNA sequencing libraries construction by using the NEBNext® UltraTM RNA Library Prep kit for Illumina® (NEB, Ipswich, MA, USA), and all the procedures and standards were performed following the manufacturer's protocols. After quality control, all libraries were sequenced on an Illumina Hiseq 2500 platform and 150 bp paired-end reads were generated. The clean reads were obtained by using FastQC software version 1.3 with the default parameters [29].

### 2.3. Alternative Splicing Analysis

The replicate multivariate analysis of transcript splicing (rMATS) v4.0.2 [21] was used to screen alternative splicing events across different samples. First, all clean reads were aligned to the *Sus scrofa* reference genome using TopHat v2.0.13 with default parameters [30]. Second, the aligned reads were run on rMATS v4.0.2 for alternative splicing analysis. The obtained ASEs were classified into five types of ASEs including skipped exon (SE), retained intron (RI), alternative 5′ splice sites (A5SS), alternative 3′ splice sites (A3SS), and mutually exclusive exons (MXE). The differential ASEs between the PRRSV infected group and the control group were identified by change in the percent in (∆PSI), which is a common parameter to describe the degree of alternative splicing. The value of ∆PSI were estimated as in the following formula:

(1) $$\begin{array}{c} \text{PSI}=\frac{I/l_{I}}{\left(I/l_{I}\right)+\left(S/l_{S}\right)},\\ \left|\Delta \text{PSI}\right|=\left|{\text{PSI}}_{\text{Infection}}-{\text{PSI}}_{\text{Control}}\right|. \end{array}$$

*I* represents the count of reads specific to the splicing transcript. *S* represents the count of reads specific to the reference transcript. *l*_*I*_ represents the length of splicing transcript, and *l*_*S*_ represents the length of reference transcript. |ΔPSI| means the absolute value of the change in the percent-spliced-in PSI_Infection_ represents the mean PSI value of an ASE in the PRRSV infected group. PSI_Control_ represents the mean PSI value of an ASE in the control group.

For pairwise comparison, we performed the –c 0.0001 parameter to compute *P* value of ASEs in rMATs running and then extracted the ASEs with a *P* value <0.05 and a |ΔPSI| ≥ 10% for the following functional enrichment analysis.

### 2.4. Enrichment of GO Category and KEGG Pathway

The Database for Annotation, Visualization and Integrated Discovery (DAVID) version 6.7 (https://david.ncifcrf.gov/) was used and Bonferroni correction was applied to obtained adjusted *P* values. The genes containing differential ASEs were sorted by the enrichment of Gene ontology (GO) categories and KEGG pathway database. The human homologous genes were used to do functional analysis in this study.

### 2.5. Alternative Splicing Factor Analysis

All porcine splicing factor genes were collected on ENSEMBL database (http://asia.ensembl.org/index.html) and analyzed their expression by fragments per kilobase of transcript per million mapped reads (FPKM) value. Genes with an adjusted *P* values <0.05 and an absolute fold change >1.5 were considered as significantly differentially expressed in this study.

### 2.6. Validation of Alternative Splicing Events

The expression of candidate genes with ASEs was calculated by FPKM value, and cDNA was reverse-transcribed using the PrimeScript RT Reagent Kit (TaKaRa Biotechnology Co. Ltd., Beijing, China) according to the manufacturer's protocol. Primers for semiquantitative PCR were designed using Primer Premier version 5. Glyceraldehyde-3-phosphate dehydrogenase (GAPDH) was used as an internal control for normalization. A list of all primers and product sizes is included in [Supplementary-material supplementary-material-1]. Semiquantitative PCR reaction mixture contained the following: 1 × PCR buffer (Mg2^+^), 0.1 mM dNTP mixture, 1 unit of TaKaRa Taq (TaKaRa Biotechnology Co. Ltd., Beijing, China), 100 nM of forward and reverse primers, 1 *μ*L cDNA template, and sterile water to reach 10 *μ*L. The PCR cycling conditions were as follows: 95°C for 5 min, followed by variable cycles (20 to 30) of 95°C for 30 s, 60°C for 30 s, 72°C for 30 s, and a final extension of 3 min at 72°C. Then the PCR products were electrophoresed in 2% agarose gel. DNA molecules were recovered from the gel and sequenced in TsingKe biological technology company.

## 3. Results

### 3.1. Increasing Global Alternative Splicing Events in Peripheral Immune Organs in response to PRRSV Infection

**Figure 1:**
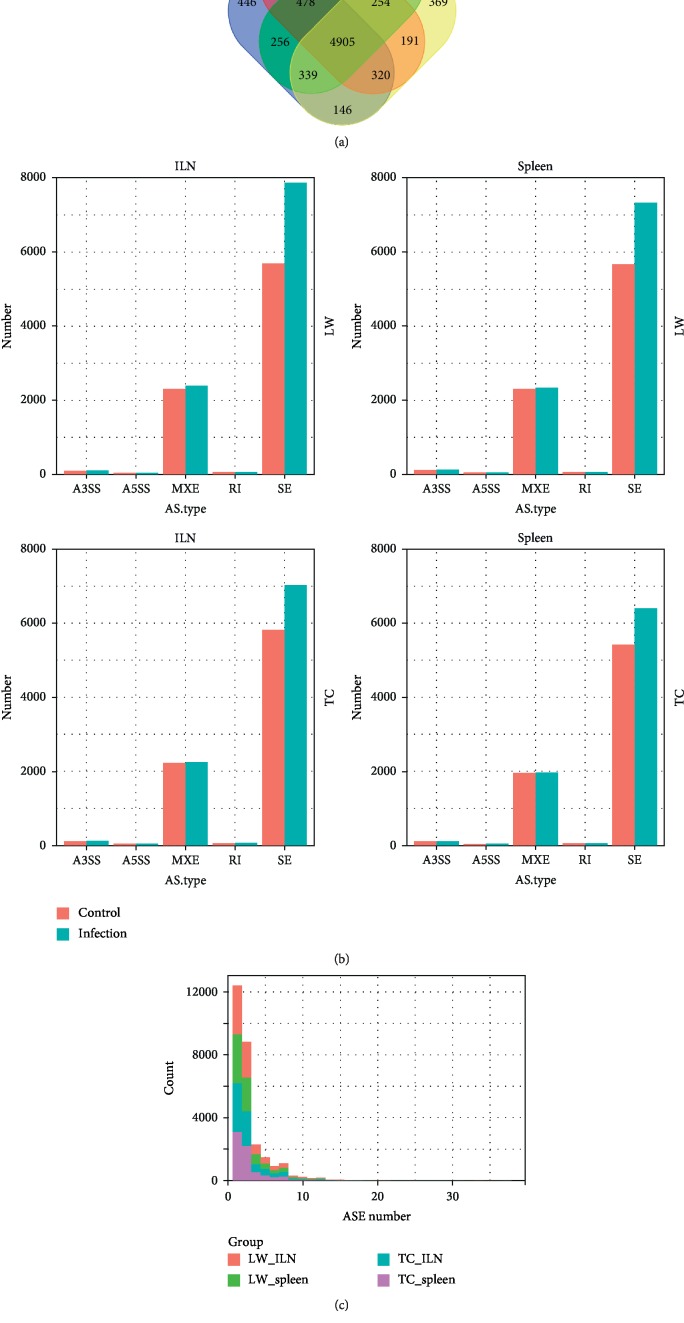
The number and distribution of alternative splicing events. (a) Number of ASEs detected in the ILN and spleen of LW pigs and TC pigs; (b) distribution of five ASE types in the ILN and spleen of LW pigs and TC pigs; (c) distribution of ASE among genes in the ILN and spleen of LW pigs and TC pigs. LW_ILN: inguinal lymph nodes of LW pigs, LW_spleen: spleen of LW pigs, TC_ILN: inguinal lymph nodes of TC pigs, TC_spleen: spleen of TC pigs, A3SS: alternative 3′ splice site, A5SS: alternative 5′ splice sites, MXE: mutually exclusive exons, RI: retained intron, SE: skipped exon.

In total, twenty-four RNA-seq libraries were used to analyze alternative splicing events in peripheral immune organs in response to PRRSV infection. The ILN transcriptome dataset contains twelve samples from the control group (LW_ILN_C and TC_ILN_C) and the PRRSV infected group (LW_ILN_I and TC_ILN_I), while the spleen transcriptome dataset also contains twelve samples from the control group (LW_S_C and TC_S_C) and the PRRSV infected group (LW_S_I and TC_S_I), and each group contained three individuals for analysis. Furthermore, we have identified 20781 and 19475 ASEs, belonging to 7175 and 7040 genes in the ILN of LW pigs and TC pigs, respectively. Meanwhile, 19730 and 17888 ASEs were identified in the spleen of LW pigs and TC pigs, which were assigned to 6991 and 6766 genes, respectively. The Venn diagram showed the four datasets had 4905 genes in common, accounting for average of 70%, while only about 5.6% ASE genes were unique in each dataset (Figure 1(a) and Table 1). Generally, ASEs were categorized as five types including A3SS, A5SS, MXE, RI, and SE. It can be noticed that the number of SE accounted for the highest proportion in all four datasets (average 72%), followed by MXE (25%), but there were less than 3% ASEs belonging to A3SS, A5SS, and RI (Figure 1(b) and Table 1). Among the five AS types, SE in PRRSV infected groups was significantly more than in control groups regardless of the tissues and breeds. Besides, the percentage of SE in LW pigs was much higher in the ILN (38.4%) and the spleen (29.3%), compared to only 20.7% and 18.0% in TC pigs. However, the difference of ASE genes between the ILN and the spleen was barely noticeable. The number of ASEs per gene, ranging from 1 to 37, experienced a gradual downward trend in the frequency. It was clear that the single ASE genes accounted for the largest rate, about 46.6%, and almost 98.2% ASE genes had less than ten ASEs. Furthermore, the maximum number of ASEs per gene was found in *EIF4G1*, which underwent 37 ASEs in total.

### 3.2. Comparison of Differential Alternative Splicing Events between TC Pigs and LW Pigs in response to PRRSV Infection

**Figure 2:**
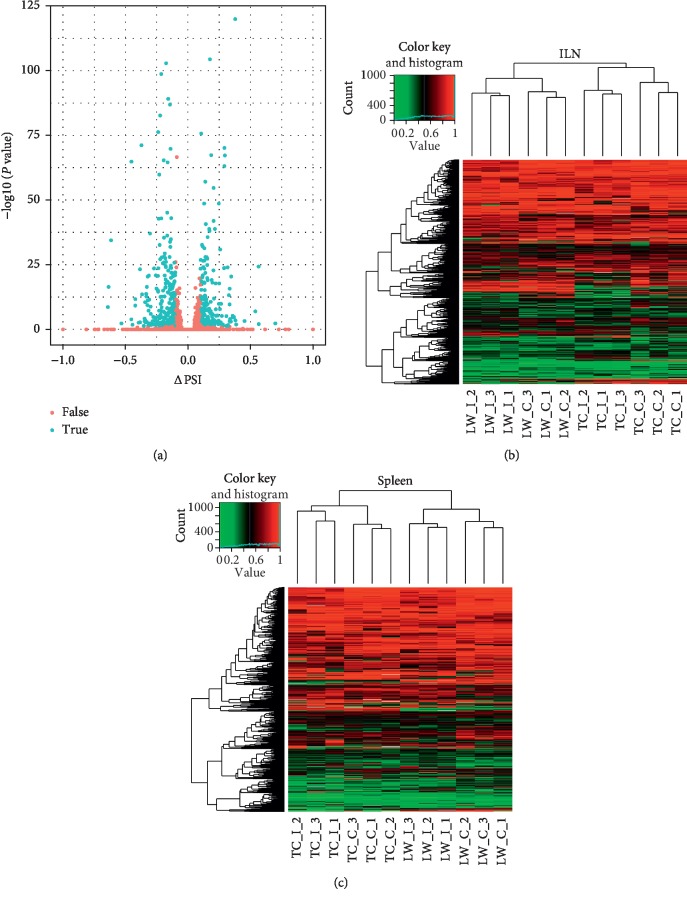
Differential alternative splicing events analysis on splicing level in response to PRRSV infection. (a) Volcano plots analysis of the differential ASEs in response to PRRSV infection. ASEs were detected and quantified using the percent-spliced-in (PSI) metric, and the blue dots indicated differential ASEs; (b) heatmap analysis of the PSI distribution of differential ASEs in the ILN; (c) heatmap analysis of the PSI distribution of differential ASEs in the spleen.

**Figure 3:**
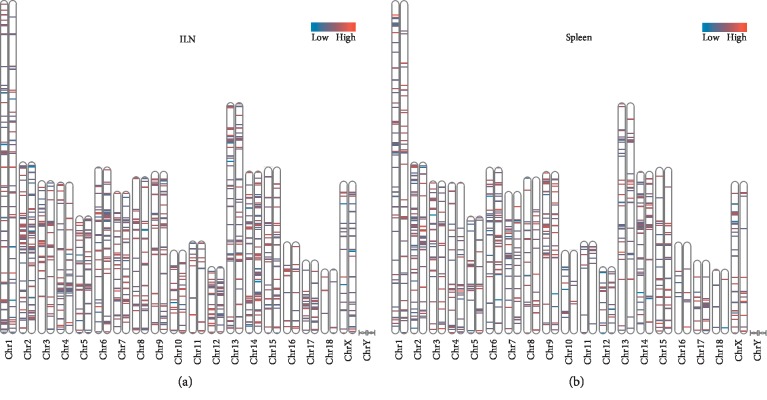
Distribution of differential alternative splicing genes among chromosomes in response to PRRSV infections. (a) Mapping ΔPSI of differential ASGs on chromosomes in the ILN; (b) mapping ΔPSI of differential ASGs on chromosomes in the spleen; the left chromosome on each paired chromosome represents LW pigs, and the right one represents TC pigs. Lines on chromosomes represent differential ASEs.

In order to identify the differential ASE pattern between TC pigs and LW pigs in response to PRRSV infection, a stringent cutoff (|ΔPSI| ≥ 10% and *P* value <0.05) was set to select differential ASEs (Figure 2(a)). Six hundred and fifty and 530 ASEs in the ILN and spleen of LW pigs were selected as differential ASEs, as well as 697 and 434 differential ASEs in the ILN and spleen of TC pigs were selected. Moreover, there were 560 genes and 458 genes containing significant differential ASEs in the ILN and spleen of LW pigs, while TC pigs had 595 differential alternative splicing genes (ASGs) and 373 differential ASGs in the ILN and spleen. One hundred and fifty-seven common differential ASGs were identified in both of the ILN and spleen of LW pigs (Tables [Supplementary-material supplementary-material-1] and [Supplementary-material supplementary-material-1]). However, 403 and 301 differential ASGs were only expressed in the ILN and spleen, respectively. Moreover, TC pigs also displayed the same trend that only 122 differential ASGs were shared by the ILN and spleen, accounting for 20% in the ILN dataset and 33% in the spleen dataset. In the ILN dataset, TC pigs and LW pigs shared 170 differential ASGs, accounting for 28% in TC pigs and 30% in LW pigs. In the spleen dataset, TC pigs and LW pigs shared 109 differential ASGs, accounting for 29% in TC pigs and 24% in LW pigs. Our results clearly showed lineage-specific and tissue-specific alternative splicing regulation in host response to PRRSV infection (Figures 2(b) and 2(c)). Then, we analyzed the ASE types in the above differential ASGs (Tables [Supplementary-material supplementary-material-1] and [Supplementary-material supplementary-material-1]), and the results showed that the SE was still dominated in differential ASGs, but its proportion decreased (53.5% in the ILN of LW pigs, 63.2% in the spleen of LW, 46.6% in the ILN of TC pigs, and 64.5% in the spleen of TC pigs). Besides, the proportion of MXE increased significantly (44.3% in the ILN of LW pigs, 33.6% in the spleen of LW, 50.8% in the ILN of TC pigs, and 33.9% in the spleen of TC pigs), and the frequency of the other three ASE types (A3SS, A5SS, and RI) in each breed and tissue is less than or equal to 10 ([Supplementary-material supplementary-material-1]). Furthermore, we have investigated the distribution of differential ASEs across pig genome. As shown in Figure 3, the distribution of genes varies from different chromosomes. For example, the largest number of differential ASGs was located on chromosome 2, harboring 71 differential ASGs in the ILN of LW pigs, 69 differential ASGs in the ILN of TC pigs, 62 differential ASGs in the spleen of LW pigs, and 43 differential ASGs in the spleen of TC pigs. Chromosome 18 contains differential ASGs at the lowest proportion for only 4 differential ASGs in the ILN of LW pigs, 6 differential ASGs in the ILN of TC pigs, 4 differential ASGs in the spleen of LW pigs and 6 differential ASGs in the spleen of TC pigs.

### 3.3. Functional Enrichment Analysis of Alternative Splicing Genes between TC Pigs and LW Pigs in response to PRRSV Infection

**Figure 4:**
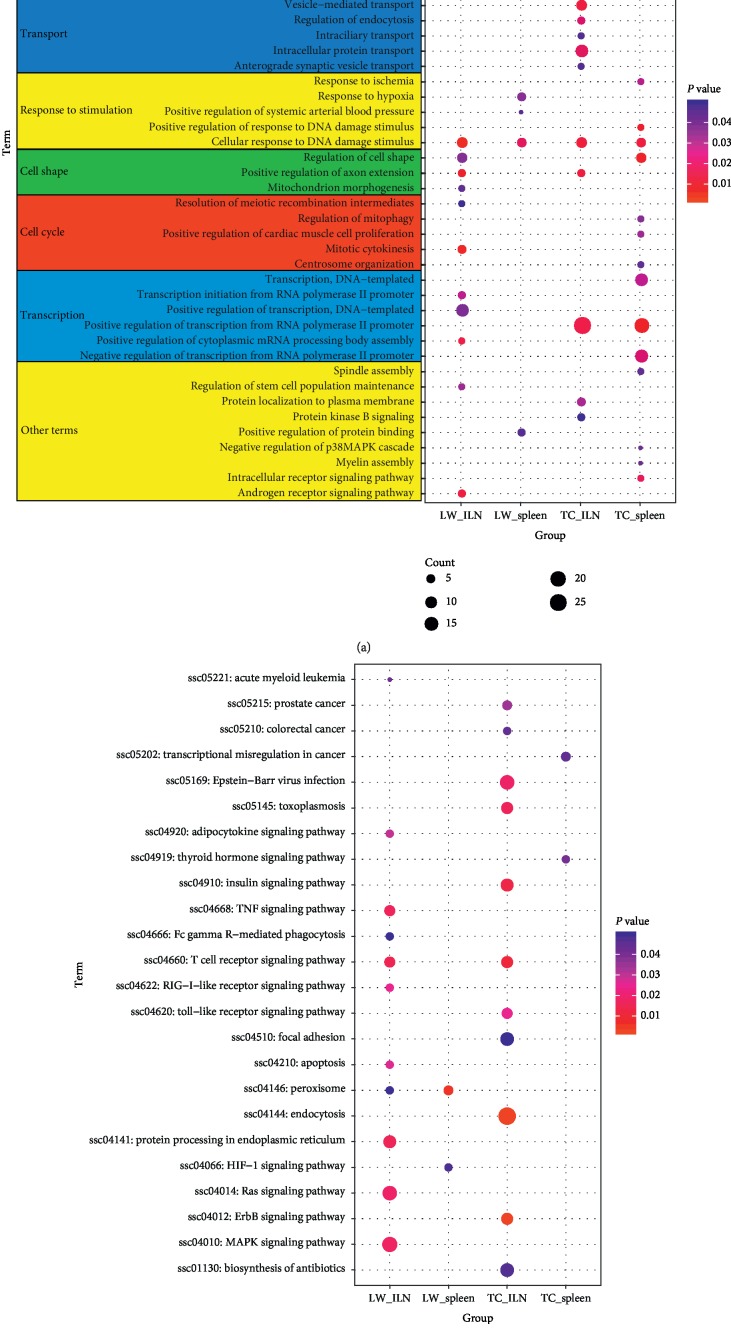
GO and KEGG pathway enrichment of differential alternative splicing genes. (a) GO enrichment analysis results; (b) KEGG pathway enrichment analysis results. The *X*-axis represents four groups, including ILN_LW (ILN of LW pigs), ILN_TC (ILN of TC pigs), spleen_LW (spleen of LW pigs), spleen_TC (spleen of TC pigs). The color represents the *P* value, and the size represents the number of gene.

To further investigate the biological functions of genes with differential ASEs, we performed GO analysis and KEGG enrichment analysis by DAVID. The results of GO functional enrichment were shown in Figure 4(a); the differential ASGs of LW pigs in response to PRRSV infection were significantly enriched in important protein posttranslational modifications including protein phosphorylation, autophosphorylation, and ubiquitination. The differential ASGs also were enriched in programmed cell death including response to tumor necrosis factor (*MAP2K7*, *YTHDC2*, *CHI3L1*), extrinsic apoptotic signaling pathway (*HSF4*, *HUNTINGTIN*, *ZYMND11*, and *PELI3*) and autophagosome assembly. These GO terms play vital roles in host cell apoptosis and protein metabolism in response to virus infection. While the differential ASE genes of TC pigs were assigned to GO terms related to tissue development including brain, embryonic organ, and nervous system development, and biological process involved in material transport such as anterograde synaptic vesicle transport, intracellular protein transport, intraciliary transport, endocytosis, and vesicle-mediated transport, which were associated with the host immune response to PRRSV infection. Among LW and TC pigs, differential ASGs were enriched in GO terms related to changes in cell morphology, especially in axon extension, mitochondrion morphogenesis, cell shape, and response to intracellular environmental stimuli such as hypoxia, ischemia, and DNA damage stimulus. In addition, differential ASGs were also widely involved in cell proliferation and transcription related GO terms in both of LW pigs and TC pigs ([Supplementary-material supplementary-material-1]). Meanwhile, the KEGG pathway analysis identified that there are great differences among different breeds and tissues. As shown in Figure 4(b), differential ASGs in the ILN were significantly enriched in T cell receptor signaling pathway in both LW pigs (*MAP2K7*, *IKBKG*, *TEC*, *PAK1*, *FYN*, *AKT2*, and *CD4*) and TC pigs (*MAP2K7*, *IKBKG*, *NFKBIE*, *PIK3CA*, and *DLG1*). Differential ASGs in the ILN of LW pigs were uniquely enriched in TNF signaling pathway (*RIPK1*, *AKT2*, *TRAF2*, *IKBKG*, *MAP2K7*, *FLIP-L*, and *CASP10*), apoptosis (*RIPK1*, *AKT2*, *TRAF2*, *IKBKG*, *FLIP-L*, and *CASP10*), MAPK signaling pathway, and RIG-I-like receptor signaling pathway (*RIPK1*, *TRAF2*, *SIKE1*, *IKBKG*, and *CASP10*). Compared with LW pigs, KEGG pathway analysis in the ILN of TC pigs revealed significant enrichment in endocytosis, ErbB signaling pathway, Toll-like receptor signaling pathway (*RIPK1*, *TLR4*, *CD40*, *MAPK8*, *TLR8*, *IKBKG*, *MAP2K7*, and *PIK3CA*) and Epstein-Barr virus infection (*RIPK1*, *SLA-6*, *NFKBIE*, *PSMC6*, *CD40*, *MAPK8*, *ENTPD1*, *GSK3B*, *IKBKG*, *MAP2K7*, *POLR2B*, and *PIK3CA*). Besides, differential ASGs in the spleen of LW pigs tended to involve peroxisome and HIF-1 signaling pathway, while the thyroid hormone signaling pathway and transcriptional misregulation in cancer were enriched in TC pigs ([Supplementary-material supplementary-material-1]).

### 3.4. Differential Expression of Splicing Factors between TC Pigs and LW Pigs

**Figure 5:**
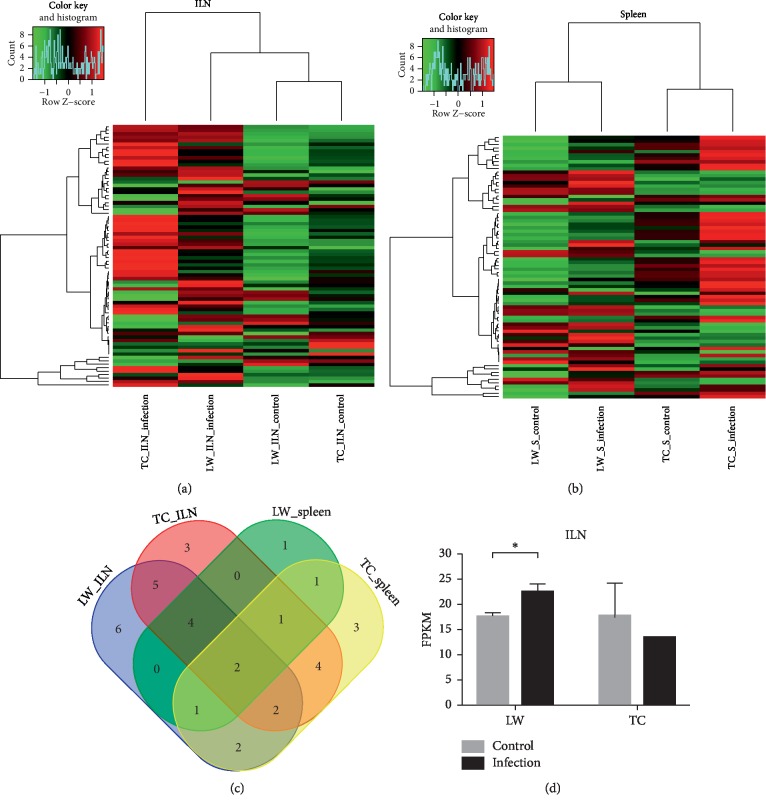
Differentially expressed splicing factors analysis in response to PRRSV infection. (a) Cluster analysis of differentially expressed alternative splicing factors in the ILN; (b) cluster analysis of differentially expressed alternative splicing factors in spleen; (c) comparison of differentially expressed alternative splicing factors in the ILN and spleen of LW pigs and TC pigs; (d) expression of *SRSF4* in the ILN of TC pigs and LW pigs. Expression of each ASE was converted to Z scores.

In order to determine whether alternative splicing factors play important roles in regulating the transcriptome difference between TC pigs and LW pigs, the expression of alternative splicing factors was investigated in this study. We identified 22 and 10 differentially expressed splicing factors in the ILN and spleen of LW pigs after PRRSV infection, respectively. Meanwhile, we also identified 23 and 16 differentially expressed splicing factors in the ILN and spleen of TC pigs after PRRSV infection, respectively. Among them, *HNRNPU* and *DHX38* were upregulated in the ILN and spleen of both breeds, while *HNRNPLL* is downregulated. Interestingly, most of the differentially expressed splicing factors were upregulated after PRRSV infection, but there were different responses between TC pigs and LW pigs to PRRSV infection. For example, *SRSF4* was upregulated in the ILN of LW pigs but had no significant expression differences in the ILN of TC pigs and downregulated in the spleen of TC pig after PRRSV infection (Figure 5(d)). Expression information of splicing factors is presented in [Supplementary-material supplementary-material-1].

### 3.5. Verification of Differential ASEs in TC Pigs and LW Pigs

**Figure 6:**
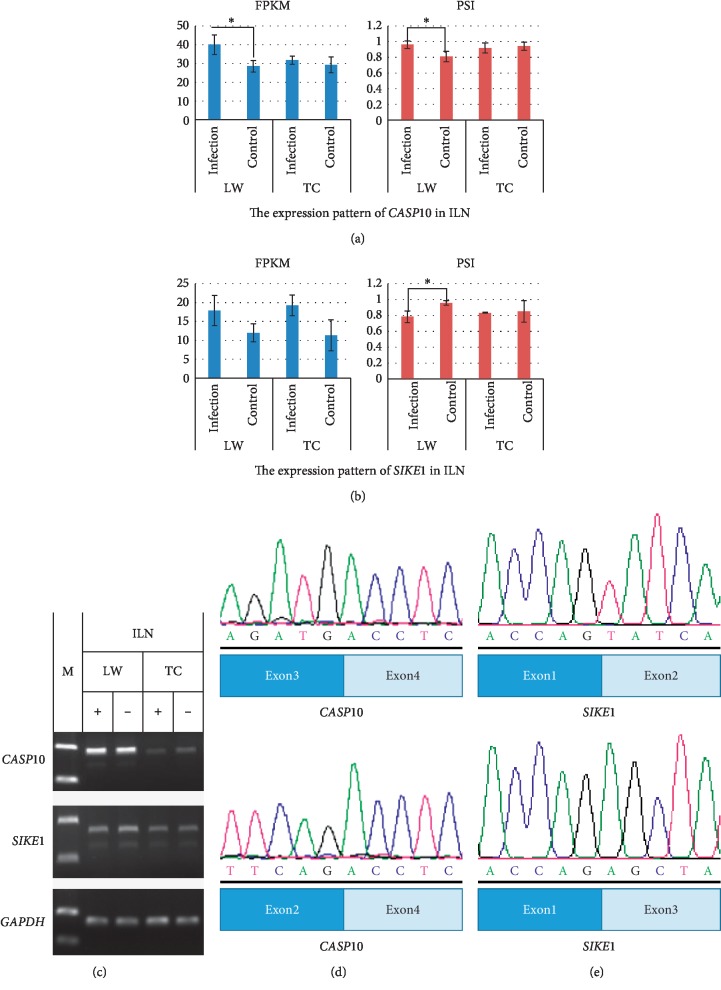
Verification of alternative splicing events in *CASP10* and *SIKE1*. (a) Semiquantitative PCR of *CASP10* and *SIKE1* in the ILN; (b) sequencing results of splicing sites in different transcripts of *CASP10*; (c) DNA sequencing results of splicing sites in different transcripts of *SIKE1*. (d) The expression of *CASP10* in the ILN; (e) the expression of *SIKE1* in the ILN.

The ASEs reported in this study were validated via PCR and DNA sequencing. Genes involving apoptotic and immune response pathways including *CASP10* and *SIKE1* were selected for validation. *CASP10* encodes Caspase-10 and directly affects the apoptotic process in different cells [31, 32]. Through PCR reaction and DNA sequencing, we found that exon 3 of *CASP10* (ENSSSCT00000053464) was deleted in the spliced transcripts, which resulted in frame-shifting mutation and lost its original function (Figure 6(c)). From transcriptome data of LW pigs, both the expression level of functional transcripts and the value of PSI in *CASP10* were increased in the infected group but no significant change occurred in TC pigs after PRRSV infection (Figure 6(a)). *SIKE1*, a critical member in the RIG-I-like receptor signaling pathway, acts as a suppressor of *TLR3* and virus-triggered interferon activation pathways by interacting with IKK-epsilon and *TBK1* [33]. The spliced transcript of *SIKE1* (ENSSSCT00000045016) generated a truncated protein with the loss of exon 2. Moreover, its splicing levels were significantly different between the infection and control group in LW pigs (Figure 6(b)). After PCR validation, we sequenced PCR products and aligned them with transcript reference sequences. The results showed that the transcript sequence generated by splicing was consistent with the predictions (Figures 6(d) and 6(e)). The sequencing results are shown in [Supplementary-material supplementary-material-1].

## 4. Discussion

Alternative splicing has been reported to play a critical role in producing protein diversity, regulating development and growth, and controlling responses to different stimuli by regulating gene expression and increasing the diversity of transcripts. Many viruses can widely modify splicing patterns of many cell transcripts involved in gene expression and RNA processing [34–38]. Alternative splicing related to viral infection was usually caused by manipulation of the spliceosome or host response to viral infection [39]. In this study, we used the transcriptome data of different peripheral immune organs to reveal that alternative splicing events were significantly increased in response to PRRSV infection, especially the increase in the frequency of SE type. Previous reports have revealed that SE was always the most frequent alternative splicing event in a large number of animal studies [20, 40]. It has been reported that the splicing level of host RNA will be changed when the virus invades the organism, which may be caused by the direct effect of the virus and the role of the immune response of the organism [4]. Therefore, we can speculate that PRRSV will significantly affect the host alternative splicing regulation.

In this study, we have reported 9184 alternative splicing genes, which indicated that ASEs were widespread in organisms and related to most biological activities. *EIF4G1* was identified as the gene with most ASEs among all alternative splicing genes. The protein encoded by *EIF4G1* is a component of the multisubunit protein complex EIF4F. The complex can inhibit viral replication in many viral infections [41]. Alternative splicing is subject to tissues-specific and lineage-specific regulation in primates [42]. We found that the number of differential ASGs in the spleen was less than that in the ILN in both of TC pigs and LW pigs. Similar results have been observed in previous studies. Human transcriptome reported that 10–30% of alternative splicing genes showed evidence of tissue-specific splice form [43]. Human virus infection studies indicated specific alternative splicing in different tissues in response to viral infection [20]. Tissue-specific alternative splicing is regulated by splicing regulatory elements and tissues-specific RNA-binding factors, which result in generating tissue-specific proteins. SE was considered to be one of the most important ASEs in regulating tissue-specific alternative splicing events between different tissues [20]. Previous studies have demonstrated that exons regulation transcripts mediated by tissue-specific alternative splicing can significantly remodel protein-protein interaction [44, 45]. Moreover, the shared differential ASGs of TC pigs and LW pigs in response to PRRSV infection were about 170 genes, almost 30% of total differential ASGs, which indicated lineage-specific alternative splicing regulation between TC pigs and LW pigs. Researches have shown that alternative splicing was tightly regulated and the expression of different splice forms was frequently changed between species [42]. Porcine myoneurin (*MYNN*) was identified to express different alternative splicing isoforms in LW pigs and Mashen (MS) pigs [28]. Functional enrichment of differential ASGs in different tissues and different breeds also proved tissue-specific and linage-specific alternative splicing regulation in response to PRRSV infection. Differential ASGs in the ILN of both TC pigs and LW pigs have enriched more functional pathways compared with differential ASGs in the spleen of both TC pigs and LW pigs. The functional pathway was mainly enriched in the TNF signaling pathway, apoptotic pathway, RIG-I receptor signaling pathway, and MAPK signaling pathway in the ILN of LW pigs. However, ASE genes in the ILN of TC pigs were mainly enriched in the Toll-like receptor signaling pathway. Several studies have shown that alternative splicing could affect host immune response mainly through apoptosis-related biological processes [46], DNA damage response [47, 48], and RIG-I pathway [49, 50]. These results were consistent with our previous results that TC pigs and LW pigs had difference immune response to PRRSV infection [16].

Splicing factors are critical for alternative splicing regulation because they can bind to short regulatory motifs on the pre-mRNA and activate alternative splicing events [51]. *SRSF4* was significantly upregulated only in the ILN of LW pigs in response to PRRSV infection, but there was no significant change in TC pigs. *SRSF4* was reported to act as a splicing regulator of Caspase-8 and mediates Caspase-8 induced apoptosis as a proapoptotic protease [52]. *SRSF4* can regulate alternative splicing induced by cisplatin and contribute to apoptosis [53]. *SRSF4* was significantly upregulated only in the ILN of LW pigs, which was consistent with the result that TC pigs and LW pigs performed different apoptosis regulation in response to PRRSV infection in our previous result [17].

## Figures and Tables

**Table 1:** Alternative splicing events in the ILN and spleen of LW pigs and TC pigs.

Tissue	ASE type	LW-I			LW-C			TC-I			TC-C		
		L1	L2	L3	L4	L5	L6	T1	T2	T3	T4	T5	T6
ILN	A3SS	109	95	103	90	92	91	120	120	111	115	121	102
	A5SS	46	42	40	40	36	38	52	43	45	46	42	40
	MXE	2375	2421	2374	2325	2299	2286	2251	2247	2250	2235	2230	2203
	RI	60	50	63	59	59	59	63	62	63	60	62	57
	SE	7600	8351	7663	6136	5446	5484	7406	6980	6686	6979	5934	4539

Spleen	A3SS	126	127	117	123	113	112	106	115	103	111	113	108
	A5SS	50	49	49	46	48	45	43	43	40	38	37	37
	MXE	2341	2326	2331	2313	2307	2306	1935	1979	1968	1949	1949	1966
	RI	61	58	64	66	61	60	58	60	56	55	58	57
	SE	7095	7863	7051	6014	5349	5658	5653	7273	6263	4986	5346	5933

L1-L6 represents different individuals from LW pigs; T1-T6 represents different individuals from TW pigs.

## Data Availability

The raw RNA-seq data for this study have been submitted to SRA database (https://www.ncbi.nlm.nih.gov/sra) under accession no. PRJNA488960.

## Supplementary Materials

Supplementary Materials Table S1: PCR Primers used in the validation of alternative splicing transcripts. Table S2: differential ASE Statistics upon PRRSV infection in different groups. Table S3: information of differential ASEs upon PRRSV infection. Table S4: detailed information of enriched GO terms belonging to biological process by ASE genes. Table S5: description of KEGG pathways enrichment by ASE genes. Table S6: expression levels of splicing factors in the ILN and spleen of TC pigs and LW pigs upon PRRSV infection. Figure S1: (a) CASP10.SPLICING.fasta; (b) SIKE1.SPLICING.fasta.
