# Supplementary material for: Global Analysis of Alternative Splicing Difference in Peripheral Immune Organs between Tongcheng Pigs and Large White Pigs Artificially Infected with PRRSV In Vivo
Source: Biomed Res Int. 2020 Jan 30;2020:4045204. doi: 10.1155/2020/4045204 (PMC7011390; doi:10.1155/2020/4045204)
Supplement: Supplementary Materials — Table S1: PCR Primers used in the validation of alternative splicing transcripts. Table S2: differential ASE Statistics upon PRRSV infection in different groups. Table S3: information of differential ASEs upon PRRSV infection. Table S4: detailed information of enriched GO terms belonging to biological process by ASE genes. Table S5: description of KEGG pathways enrichment by ASE genes. Table S6: expression levels of splicing factors in the ILN and spleen of TC pigs and LW pigs upon PRRSV infection. Figure S1: (a) CASP10.SPLICING.fasta; (b) SIKE1.SPLICING.fasta. [file 4045204.f1.zip › FigureS1.docx]

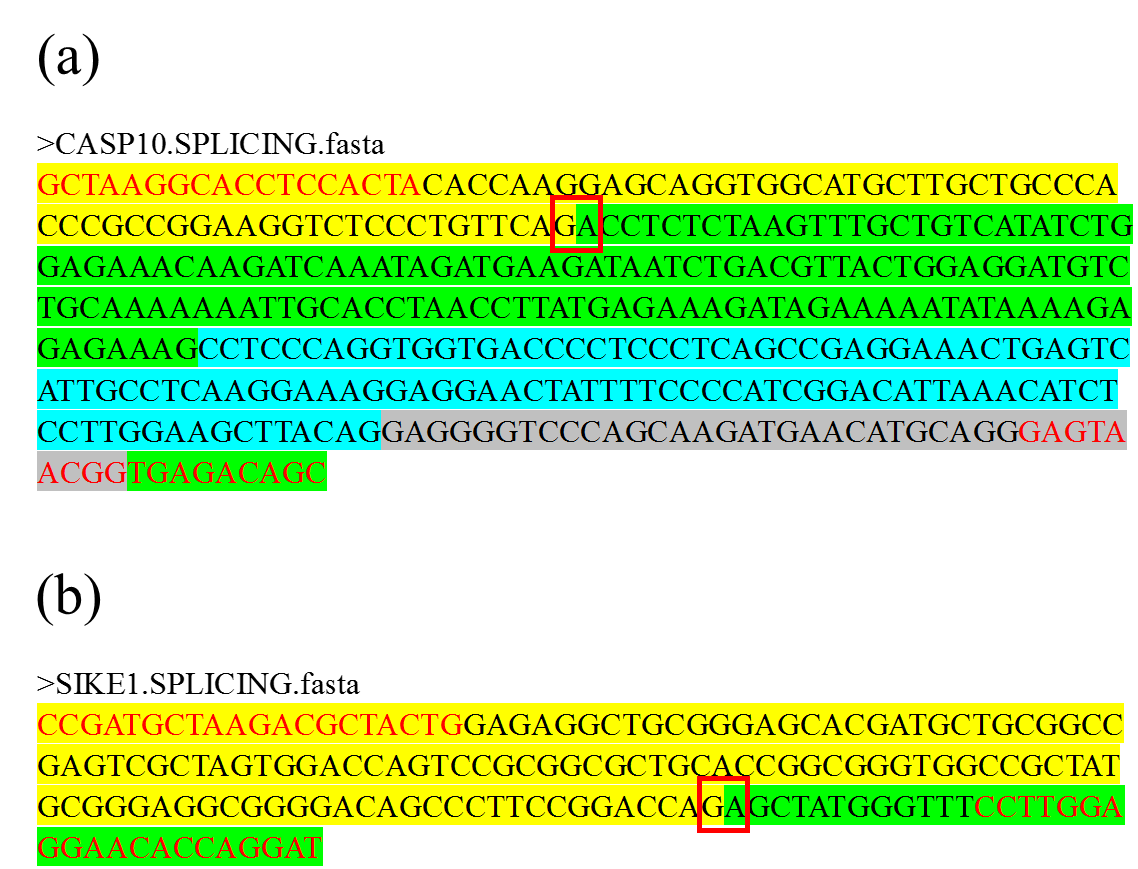


**Figure S1 Sequence information of splicing transcripts in *CASP10* and *SIKE1***. (a) Nucleotide sequence of *CASP10* splicing transcript by Sanger sequencing; (b) Nucleotide sequence of *SIKE1* splicing transcript by Sanger sequencing; Red characters represent sequences of PCR primers; Different colors represent different exons and red boxes represent splicing sites.
