# Supplementary material for: Global Analysis of Alternative Splicing Difference in Peripheral Immune Organs between Tongcheng Pigs and Large White Pigs Artificially Infected with PRRSV In Vivo
Source: Biomed Res Int. 2020 Jan 30;2020:4045204. doi: 10.1155/2020/4045204 (PMC7011390; doi:10.1155/2020/4045204)
Supplement: Supplementary Materials — Table S1: PCR Primers used in the validation of alternative splicing transcripts. Table S2: differential ASE Statistics upon PRRSV infection in different groups. Table S3: information of differential ASEs upon PRRSV infection. Table S4: detailed information of enriched GO terms belonging to biological process by ASE genes. Table S5: description of KEGG pathways enrichment by ASE genes. Table S6: expression levels of splicing factors in the ILN and spleen of TC pigs and LW pigs upon PRRSV infection. Figure S1: (a) CASP10.SPLICING.fasta; (b) SIKE1.SPLICING.fasta. [file 4045204.f1.zip › Supplementary_material_illustration.docx]

**Supplementary Materials**

**Table S1** Primers used in this study.

**Table S2** Statistics of differential ASEs upon PRRSV infection.

**Table S3** Information of significant differential ASE genes between PRRSV-infected and control groups.

**Table S4** Significantly enriched biological process GO terms of differential ASE genes in ILN and spleen between LW pigs and TC pigs upon PRRSV infection.

**Table S5** Significantly enriched KEGG pathways of differential ASE genes in ILN and spleen between LW pigs and TC pigs upon PRRSV infection.

**Table S6** Expression levels of splicing factors in ILN and Spleen of TC pigs and LW pigs upon PRRSV infection.

**Figure S1** Sequence information of splicing transcripts in *CASP10* and *SIKE1.*
