# Supplementary material for: Global Analysis of Alternative Splicing Difference in Peripheral Immune Organs between Tongcheng Pigs and Large White Pigs Artificially Infected with PRRSV In Vivo
Source: Biomed Res Int. 2020 Jan 30;2020:4045204. doi: 10.1155/2020/4045204 (PMC7011390; doi:10.1155/2020/4045204)
Supplement: Supplementary Materials — Table S1: PCR Primers used in the validation of alternative splicing transcripts. Table S2: differential ASE Statistics upon PRRSV infection in different groups. Table S3: information of differential ASEs upon PRRSV infection. Table S4: detailed information of enriched GO terms belonging to biological process by ASE genes. Table S5: description of KEGG pathways enrichment by ASE genes. Table S6: expression levels of splicing factors in the ILN and spleen of TC pigs and LW pigs upon PRRSV infection. Figure S1: (a) CASP10.SPLICING.fasta; (b) SIKE1.SPLICING.fasta. [file 4045204.f1.zip › TableS1.docx]

**Table S1 Primers used in this study.**

| Gene | GenBank ID | Primer Name | Primer sequences (5'→3') | Product size (bp) |
| --- | --- | --- | --- | --- |
| SIKE1 | XM_001924186.5 | SIKE1-F | CCGATGCTAAGACGCTACTG | Reference transcript: 269 |
|  |  | SIKE1-R | ATCCTGGTGTTCCTCCAAGG | Splicing transcript: 163 |
| CASP10 | XM_021074526.1 | CASP10-F | GCTAAGGCACCTCCACTA | Reference transcript: 462 |
|  |  | CASP10-R | GCTGTCTCACCGTTACTC | Splicing transcript: 368 |
| GAPDH | NM_001206359.1 | GAPDH-F | CGTCCCTGAGACACGATGGT | 194 |
|  |  | GAPDH-R | GCCTTGACTGTGCCGTGGAAC |  |
