# Supplementary material for: Global Analysis of Alternative Splicing Difference in Peripheral Immune Organs between Tongcheng Pigs and Large White Pigs Artificially Infected with PRRSV In Vivo
Source: Biomed Res Int. 2020 Jan 30;2020:4045204. doi: 10.1155/2020/4045204 (PMC7011390; doi:10.1155/2020/4045204)
Supplement: Supplementary Materials — Table S1: PCR Primers used in the validation of alternative splicing transcripts. Table S2: differential ASE Statistics upon PRRSV infection in different groups. Table S3: information of differential ASEs upon PRRSV infection. Table S4: detailed information of enriched GO terms belonging to biological process by ASE genes. Table S5: description of KEGG pathways enrichment by ASE genes. Table S6: expression levels of splicing factors in the ILN and spleen of TC pigs and LW pigs upon PRRSV infection. Figure S1: (a) CASP10.SPLICING.fasta; (b) SIKE1.SPLICING.fasta. [file 4045204.f1.zip › TableS2.docx]

**Table S2 Statistics of differential ASEs upon PRRSV infection**

|  | LW_ILN | LW_Spleen | TC_ILN | TC_Spleen |
| --- | --- | --- | --- | --- |
| MXE | 288 | 178 | 354 | 147 |
| A3SS | 3 | 5 | 6 | 3 |
| A5SS | 6 | 4 | 2 | 0 |
| RI | 5 | 8 | 10 | 4 |
| SE | 348 | 335 | 325 | 280 |
| Total ASEs | 650 | 530 | 697 | 434 |
| ASE Genes | 560 | 458 | 595 | 373 |
