# Supplementary material for: Global Analysis of Alternative Splicing Difference in Peripheral Immune Organs between Tongcheng Pigs and Large White Pigs Artificially Infected with PRRSV In Vivo
Source: Biomed Res Int. 2020 Jan 30;2020:4045204. doi: 10.1155/2020/4045204 (PMC7011390; doi:10.1155/2020/4045204)
Supplement: Supplementary Materials — Table S1: PCR Primers used in the validation of alternative splicing transcripts. Table S2: differential ASE Statistics upon PRRSV infection in different groups. Table S3: information of differential ASEs upon PRRSV infection. Table S4: detailed information of enriched GO terms belonging to biological process by ASE genes. Table S5: description of KEGG pathways enrichment by ASE genes. Table S6: expression levels of splicing factors in the ILN and spleen of TC pigs and LW pigs upon PRRSV infection. Figure S1: (a) CASP10.SPLICING.fasta; (b) SIKE1.SPLICING.fasta. [file 4045204.f1.zip › TableS3.docx]

| **Table S3 Information of significantly differential ASE genes between PRRSV-infected and control groups** | | | | | |
| --- | --- | --- | --- | --- | --- |
| Group | ASE Type | GeneID | Gene Name | *P*-value | ΔPSI |
| LW_ILN | A3SS | ENSSSCG00000012149 | RBBP7 | 0.024306 | -0.116 |
| LW_ILN | A3SS | ENSSSCG00000008820 | TEC | 0.00022 | -0.341 |
| LW_ILN | A3SS | ENSSSCG00000009839 | NA | 5.88E-24 | -0.384 |
| LW_ILN | A5SS | ENSSSCG00000005122 | TEK | 3.31E-28 | -0.181 |
| LW_ILN | A5SS | ENSSSCG00000005462 | GNG10 | 0.047789 | 0.181 |
| LW_ILN | A5SS | ENSSSCG00000012825 | IKBKG | 8.59E-14 | 0.136 |
| LW_ILN | A5SS | ENSSSCG00000015815 | FGFR1 | 0.023365 | 0.172 |
| LW_ILN | A5SS | ENSSSCG00000004535 | TCF4 | 1.38E-08 | 0.165 |
| LW_ILN | A5SS | ENSSSCG00000011199 | TBC1D5 | 5.42E-06 | 0.358 |
| LW_ILN | MXE | ENSSSCG00000016069 | NA | 0.003019 | -0.117 |
| LW_ILN | MXE | ENSSSCG00000011498 | SLC25A26 | 3.14E-15 | 0.141 |
| LW_ILN | MXE | ENSSSCG00000016859 | C7 | 0.015152 | -0.113 |
| LW_ILN | MXE | ENSSSCG00000007280 | ITCH | 3.65E-07 | 0.109 |
| LW_ILN | MXE | ENSSSCG00000000981 | CRELD2 | 2.77E-05 | 0.255 |
| LW_ILN | MXE | ENSSSCG00000009567 | RASA3 | 1.23E-34 | 0.128 |
| LW_ILN | MXE | ENSSSCG00000006897 | NA | 0.000152 | -0.12 |
| LW_ILN | MXE | ENSSSCG00000001572 | FGD2 | 0.004753 | 0.125 |
| LW_ILN | MXE | ENSSSCG00000011612 | RPN1 | 0.002226 | 0.121 |
| LW_ILN | MXE | ENSSSCG00000015106 | HYOU1 | 0.000102 | 0.101 |
| LW_ILN | MXE | ENSSSCG00000016661 | SEPT7 | 1.61E-69 | 0.14 |
| LW_ILN | MXE | ENSSSCG00000022521 | DPY19L3 | 9.56E-24 | -0.187 |
| LW_ILN | MXE | ENSSSCG00000011194 | ANKRD28 | 0.00702 | -0.152 |
| LW_ILN | MXE | ENSSSCG00000010799 | COG7 | 1.23E-36 | -0.152 |
| LW_ILN | MXE | ENSSSCG00000022521 | DPY19L3 | 0.029895 | -0.124 |
| LW_ILN | MXE | ENSSSCG00000014878 | NA | 4.46E-09 | -0.122 |
| LW_ILN | MXE | ENSSSCG00000022521 | DPY19L3 | 0.007698 | -0.266 |
| LW_ILN | MXE | ENSSSCG00000017688 | GGNBP2 | 2.69E-66 | 0.134 |
| LW_ILN | MXE | ENSSSCG00000005376 | TBC1D2 | 0.009309 | -0.146 |
| LW_ILN | MXE | ENSSSCG00000005480 | NA | 1.11E-09 | 0.118 |
| LW_ILN | MXE | ENSSSCG00000011090 | NEBL | 3.98E-41 | 0.378 |
| LW_ILN | MXE | ENSSSCG00000026697 | UHRF1BP1L | 1.32E-06 | 0.102 |
| LW_ILN | MXE | ENSSSCG00000026934 | PIGX | 0.000253 | -0.125 |
| LW_ILN | MXE | ENSSSCG00000014081 | COL4A3BP | 0.005518 | -0.125 |
| LW_ILN | MXE | ENSSSCG00000018032 | TRPV2 | 7.66E-20 | -0.151 |
| LW_ILN | MXE | ENSSSCG00000017072 | GALNT10 | 0.000152 | -0.185 |
| LW_ILN | MXE | ENSSSCG00000001009 | RIPK1 | 0.00572 | 0.175 |
| LW_ILN | MXE | ENSSSCG00000011772 | NA | 2.82E-08 | -0.211 |
| LW_ILN | MXE | ENSSSCG00000016857 | DAB2 | 8.39E-10 | -0.148 |
| LW_ILN | MXE | ENSSSCG00000021170 | FAR1 | 0.001655 | 0.127 |
| LW_ILN | MXE | ENSSSCG00000026898 | NA | 3.31E-11 | 0.131 |
| LW_ILN | MXE | ENSSSCG00000015868 | ARL6IP6 | 2.54E-18 | 0.123 |
| LW_ILN | MXE | ENSSSCG00000001087 | MRS2 | 6.12E-07 | -0.202 |
| LW_ILN | MXE | ENSSSCG00000021702 | NA | 0.011294 | -0.135 |
| LW_ILN | MXE | ENSSSCG00000017264 | AMZ2 | 2.78E-23 | -0.179 |
| LW_ILN | MXE | ENSSSCG00000010854 | TMEM63A | 5.33E-07 | -0.11 |
| LW_ILN | MXE | ENSSSCG00000024881 | NA | 1.12E-11 | 0.199 |
| LW_ILN | MXE | ENSSSCG00000007146 | SIGLEC1 | 0.01249 | 0.11 |
| LW_ILN | MXE | ENSSSCG00000024756 | NA | 0.001973 | -0.19 |
| LW_ILN | MXE | ENSSSCG00000009290 | NA | 1.67E-05 | -0.161 |
| LW_ILN | MXE | ENSSSCG00000001958 | BAZ1A | 0.041092 | 0.111 |
| LW_ILN | MXE | ENSSSCG00000007383 | TOMM34 | 0.004656 | 0.15 |
| LW_ILN | MXE | ENSSSCG00000005838 | TRAF2 | 8.26E-21 | 0.114 |
| LW_ILN | MXE | ENSSSCG00000005122 | TEK | 8.55E-10 | -0.103 |
| LW_ILN | MXE | ENSSSCG00000011609 | NA | 0.022803 | 0.105 |
| LW_ILN | MXE | ENSSSCG00000009372 | MRPS31 | 1E-36 | 0.135 |
| LW_ILN | MXE | ENSSSCG00000004651 | GALK2 | 1.8E-16 | -0.233 |
| LW_ILN | MXE | ENSSSCG00000001341 | SLA-11 | 4.99E-05 | -0.13 |
| LW_ILN | MXE | ENSSSCG00000004929 | PARP16 | 0.000278 | -0.117 |
| LW_ILN | MXE | ENSSSCG00000009113 | METTL14 | 0.018727 | -0.157 |
| LW_ILN | MXE | ENSSSCG00000022784 | PLEKHO1 | 0.000108 | -0.144 |
| LW_ILN | MXE | ENSSSCG00000008237 | RETSAT | 9.07E-05 | -0.154 |
| LW_ILN | MXE | ENSSSCG00000004109 | ZC3H12D | 2.6E-13 | 0.129 |
| LW_ILN | MXE | ENSSSCG00000009330 | ALOX5AP | 2.38E-23 | 0.14 |
| LW_ILN | MXE | ENSSSCG00000011385 | NA | 2.57E-20 | 0.153 |
| LW_ILN | MXE | ENSSSCG00000017308 | CDC27 | 3.71E-43 | 0.146 |
| LW_ILN | MXE | ENSSSCG00000003580 | EYA3 | 9.87E-22 | 0.156 |
| LW_ILN | MXE | ENSSSCG00000005356 | DCAF10 | 8.2E-52 | -0.183 |
| LW_ILN | MXE | ENSSSCG00000029183 | SEC11A | 0.005361 | -0.105 |
| LW_ILN | MXE | ENSSSCG00000006457 | FCRL3 | 1.23E-06 | -0.216 |
| LW_ILN | MXE | ENSSSCG00000006457 | FCRL3 | 2.29E-10 | -0.105 |
| LW_ILN | MXE | ENSSSCG00000011881 | IQCB1 | 1.07E-08 | 0.178 |
| LW_ILN | MXE | ENSSSCG00000009084 | SPATA5 | 0.048567 | 0.116 |
| LW_ILN | MXE | ENSSSCG00000003815 | ALG6 | 0.02642 | -0.211 |
| LW_ILN | MXE | ENSSSCG00000008877 | ETFDH | 0.044836 | -0.105 |
| LW_ILN | MXE | ENSSSCG00000027287 | TMEM189 | 4.17E-70 | 0.191 |
| LW_ILN | MXE | ENSSSCG00000027287 | TMEM189 | 6.88E-05 | 0.129 |
| LW_ILN | MXE | ENSSSCG00000006703 | PRKAB2 | 1.48E-05 | -0.169 |
| LW_ILN | MXE | ENSSSCG00000006703 | PRKAB2 | 0.018869 | -0.132 |
| LW_ILN | MXE | ENSSSCG00000004634 | NA | 0.047414 | 0.135 |
| LW_ILN | MXE | ENSSSCG00000027459 | RNF13 | 0.015052 | 0.106 |
| LW_ILN | MXE | ENSSSCG00000010605 | OBFC1 | 0.02023 | 0.156 |
| LW_ILN | MXE | ENSSSCG00000013467 | SGTA | 2.01E-23 | -0.112 |
| LW_ILN | MXE | ENSSSCG00000016194 | USP37 | 2.94E-27 | -0.13 |
| LW_ILN | MXE | ENSSSCG00000010528 | ZFYVE27 | 0.000181 | -0.106 |
| LW_ILN | MXE | ENSSSCG00000014015 | C5orf45 | 7.33E-22 | 0.193 |
| LW_ILN | MXE | ENSSSCG00000014998 | AASDHPPT | 2.6E-12 | 0.1 |
| LW_ILN | MXE | ENSSSCG00000000215 | SMARCD1 | 0.002042 | 0.107 |
| LW_ILN | MXE | ENSSSCG00000014998 | AASDHPPT | 7.81E-18 | 0.108 |
| LW_ILN | MXE | ENSSSCG00000005646 | NA | 1.76E-32 | -0.259 |
| LW_ILN | MXE | ENSSSCG00000013402 | NA | 0.014498 | 0.172 |
| LW_ILN | MXE | ENSSSCG00000012122 | TCEANC | 1.79E-07 | 0.11 |
| LW_ILN | MXE | ENSSSCG00000006098 | DPY19L4 | 0.039685 | -0.129 |
| LW_ILN | MXE | ENSSSCG00000017906 | RNF167 | 3.28E-10 | -0.143 |
| LW_ILN | MXE | ENSSSCG00000027264 | NA | 5.72E-26 | 0.109 |
| LW_ILN | MXE | ENSSSCG00000013570 | MCOLN1 | 0.011754 | 0.173 |
| LW_ILN | MXE | ENSSSCG00000025463 | NA | 3.65E-12 | -0.107 |
| LW_ILN | MXE | ENSSSCG00000008493 | CEBPZ | 0.036931 | 0.1 |
| LW_ILN | MXE | ENSSSCG00000001099 | CMAH | 4.54E-15 | 0.172 |
| LW_ILN | MXE | ENSSSCG00000003848 | LRP8 | 1.13E-14 | 0.174 |
| LW_ILN | MXE | ENSSSCG00000011197 | OXNAD1 | 0.003076 | -0.12 |
| LW_ILN | MXE | ENSSSCG00000026473 | NA | 0.038094 | -0.24 |
| LW_ILN | MXE | ENSSSCG00000012984 | SCYL1 | 0.009375 | 0.132 |
| LW_ILN | MXE | ENSSSCG00000024872 | CPT1C | 0.000568 | -0.195 |
| LW_ILN | MXE | ENSSSCG00000009676 | ZNF395 | 6.9E-10 | -0.168 |
| LW_ILN | MXE | ENSSSCG00000001641 | NA | 2.28E-08 | 0.105 |
| LW_ILN | MXE | ENSSSCG00000015947 | DCAF17 | 0.01069 | -0.308 |
| LW_ILN | MXE | ENSSSCG00000025425 | FUK | 3.41E-06 | 0.105 |
| LW_ILN | MXE | ENSSSCG00000014364 | ANKHD1 | 1.65E-78 | 0.138 |
| LW_ILN | MXE | ENSSSCG00000024373 | TRIP12 | 0.00597 | -0.115 |
| LW_ILN | MXE | ENSSSCG00000013857 | EPS15L1 | 0.000396 | -0.303 |
| LW_ILN | MXE | ENSSSCG00000013857 | EPS15L1 | 5.79E-17 | -0.382 |
| LW_ILN | MXE | ENSSSCG00000025606 | NA | 2.87E-05 | -0.118 |
| LW_ILN | MXE | ENSSSCG00000030616 | KIAA1551 | 3.01E-05 | 0.183 |
| LW_ILN | MXE | ENSSSCG00000002989 | AKT2 | 0.004619 | 0.147 |
| LW_ILN | MXE | ENSSSCG00000015052 | USP28 | 0.008481 | 0.372 |
| LW_ILN | MXE | ENSSSCG00000008298 | DUSP11 | 2.8E-30 | 0.191 |
| LW_ILN | MXE | ENSSSCG00000022453 | NA | 2.28E-07 | -0.16 |
| LW_ILN | MXE | ENSSSCG00000010563 | BTRC | 1.29E-05 | 0.122 |
| LW_ILN | MXE | ENSSSCG00000008988 | CCNG2 | 0.000556 | 0.138 |
| LW_ILN | MXE | ENSSSCG00000004421 | FYN | 2.9E-15 | -0.207 |
| LW_ILN | MXE | ENSSSCG00000015023 | NA | 0.011607 | 0.188 |
| LW_ILN | MXE | ENSSSCG00000006165 | SEC31B | 0.03219 | 0.202 |
| LW_ILN | MXE | ENSSSCG00000012262 | KDM6A | 3.78E-07 | 0.138 |
| LW_ILN | MXE | ENSSSCG00000002799 | CNOT1 | 0.003494 | -0.113 |
| LW_ILN | MXE | ENSSSCG00000008415 | NA | 2.58E-48 | 0.16 |
| LW_ILN | MXE | ENSSSCG00000008221 | KDM3A | 5.65E-51 | 0.157 |
| LW_ILN | MXE | ENSSSCG00000012329 | PHF8 | 0.037369 | 0.115 |
| LW_ILN | MXE | ENSSSCG00000015721 | ERCC3 | 1.4E-59 | 0.152 |
| LW_ILN | MXE | ENSSSCG00000026940 | CASP10 | 4.89E-24 | 0.128 |
| LW_ILN | MXE | ENSSSCG00000014394 | RNF14 | 0.032646 | -0.135 |
| LW_ILN | MXE | ENSSSCG00000011552 | ARPC4 | 5.09E-10 | 0.106 |
| LW_ILN | MXE | ENSSSCG00000015789 | SNX25 | 0.029745 | -0.145 |
| LW_ILN | MXE | ENSSSCG00000009720 | DDX60 | 7.87E-45 | -0.163 |
| LW_ILN | MXE | ENSSSCG00000016567 | STRIP2 | 5.65E-17 | -0.181 |
| LW_ILN | MXE | ENSSSCG00000020675 | ATF7IP2 | 1.22E-12 | -0.19 |
| LW_ILN | MXE | ENSSSCG00000020675 | ATF7IP2 | 6.73E-05 | -0.24 |
| LW_ILN | MXE | ENSSSCG00000013299 | APIP | 4.73E-13 | 0.124 |
| LW_ILN | MXE | ENSSSCG00000000393 | PAN2 | 0.000419 | -0.166 |
| LW_ILN | MXE | ENSSSCG00000010699 | ATE1 | 7.79E-06 | -0.1 |
| LW_ILN | MXE | ENSSSCG00000024383 | HAUS4 | 3.2E-18 | -0.179 |
| LW_ILN | MXE | ENSSSCG00000002452 | NA | 1.38E-07 | 0.102 |
| LW_ILN | MXE | ENSSSCG00000011804 | RFC4 | 2.76E-69 | 0.21 |
| LW_ILN | MXE | ENSSSCG00000025952 | NA | 0.02813 | 0.132 |
| LW_ILN | MXE | ENSSSCG00000001400 | DDX39B | 1.59E-52 | 0.118 |
| LW_ILN | MXE | ENSSSCG00000011415 | MAPKAPK3 | 3.11E-11 | 0.147 |
| LW_ILN | MXE | ENSSSCG00000009473 | MYCBP2 | 3.93E-09 | 0.103 |
| LW_ILN | MXE | ENSSSCG00000028293 | RCOR3 | 0.005959 | -0.145 |
| LW_ILN | MXE | ENSSSCG00000025802 | NA | 0.000528 | 0.107 |
| LW_ILN | MXE | ENSSSCG00000007089 | MGME1 | 1.74E-05 | 0.154 |
| LW_ILN | MXE | ENSSSCG00000005983 | ATAD2 | 0.004058 | -0.105 |
| LW_ILN | MXE | ENSSSCG00000009142 | SEC24B | 0.014305 | -0.135 |
| LW_ILN | MXE | ENSSSCG00000000145 | MYH9 | 0.038566 | 0.115 |
| LW_ILN | MXE | ENSSSCG00000022990 | NA | 2.13E-12 | 0.11 |
| LW_ILN | MXE | ENSSSCG00000013772 | ASF1B | 0.000248 | 0.218 |
| LW_ILN | MXE | ENSSSCG00000017574 | NA | 0.006151 | 0.264 |
| LW_ILN | MXE | ENSSSCG00000012896 | NDUFV1 | 1.47E-09 | 0.121 |
| LW_ILN | MXE | ENSSSCG00000015135 | SORL1 | 4.62E-66 | 0.141 |
| LW_ILN | MXE | ENSSSCG00000000458 | MON2 | 3.59E-56 | 0.145 |
| LW_ILN | MXE | ENSSSCG00000000656 | CLEC2B | 0.000311 | -0.147 |
| LW_ILN | MXE | ENSSSCG00000009238 | COQ2 | 1.24E-17 | -0.11 |
| LW_ILN | MXE | ENSSSCG00000009238 | COQ2 | 6.95E-17 | -0.157 |
| LW_ILN | MXE | ENSSSCG00000027857 | DMXL1 | 2.62E-62 | 0.184 |
| LW_ILN | MXE | ENSSSCG00000000502 | CNOT2 | 0.001377 | 0.147 |
| LW_ILN | MXE | ENSSSCG00000006096 | INTS8 | 0.001934 | -0.121 |
| LW_ILN | MXE | ENSSSCG00000022830 | KANSL1L | 6.12E-20 | 0.287 |
| LW_ILN | MXE | ENSSSCG00000009834 | ATXN2 | 0.006604 | 0.205 |
| LW_ILN | MXE | ENSSSCG00000003085 | CLPTM1 | 6.24E-34 | 0.133 |
| LW_ILN | MXE | ENSSSCG00000017325 | PLEKHM1 | 2.57E-12 | 0.156 |
| LW_ILN | MXE | ENSSSCG00000004569 | LACTB | 0.000277 | 0.125 |
| LW_ILN | MXE | ENSSSCG00000011394 | RBM6 | 0.011279 | 0.112 |
| LW_ILN | MXE | ENSSSCG00000011504 | EOGT | 1.16E-07 | 0.108 |
| LW_ILN | MXE | ENSSSCG00000005505 | NA | 1.83E-11 | -0.153 |
| LW_ILN | MXE | ENSSSCG00000028117 | NA | 0.00802 | 0.215 |
| LW_ILN | MXE | ENSSSCG00000002455 | GOLGA5 | 0.040403 | -0.129 |
| LW_ILN | MXE | ENSSSCG00000025503 | ADGRG5 | 0.011114 | 0.189 |
| LW_ILN | MXE | ENSSSCG00000011873 | HSPBAP1 | 6.97E-05 | 0.168 |
| LW_ILN | MXE | ENSSSCG00000007979 | LUC7L | 0.024185 | 0.19 |
| LW_ILN | MXE | ENSSSCG00000013766 | IL27RA | 3.74E-16 | 0.109 |
| LW_ILN | MXE | ENSSSCG00000014562 | NA | 1.11E-07 | 0.343 |
| LW_ILN | MXE | ENSSSCG00000021042 | BRWD3 | 2.1E-93 | -0.178 |
| LW_ILN | MXE | ENSSSCG00000013634 | YIPF2 | 3.28E-17 | 0.111 |
| LW_ILN | MXE | ENSSSCG00000015784 | ACSL1 | 0.035518 | 0.108 |
| LW_ILN | MXE | ENSSSCG00000017743 | CRLF3 | 0.00178 | -0.124 |
| LW_ILN | MXE | ENSSSCG00000016864 | C5orf51 | 0.002755 | 0.122 |
| LW_ILN | MXE | ENSSSCG00000011162 | LARP4B | 0.000212 | 0.116 |
| LW_ILN | MXE | ENSSSCG00000013416 | MED16 | 1.32E-05 | 0.143 |
| LW_ILN | MXE | ENSSSCG00000015050 | ZW10 | 0.020476 | -0.122 |
| LW_ILN | MXE | ENSSSCG00000005934 | TRAPPC9 | 1.86E-12 | 0.444 |
| LW_ILN | MXE | ENSSSCG00000007004 | INTS10 | 0.034617 | -0.118 |
| LW_ILN | MXE | ENSSSCG00000030354 | MAP3K2 | 1.05E-25 | -0.133 |
| LW_ILN | MXE | ENSSSCG00000027270 | TIMM9 | 1.32E-28 | 0.152 |
| LW_ILN | MXE | ENSSSCG00000023975 | C15orf52 | 0.031771 | -0.273 |
| LW_ILN | MXE | ENSSSCG00000025192 | RPL9 | 0.000345 | -0.12 |
| LW_ILN | MXE | ENSSSCG00000018049 | NA | 1.06E-07 | 0.257 |
| LW_ILN | MXE | ENSSSCG00000012743 | MTMR1 | 1.96E-08 | -0.107 |
| LW_ILN | MXE | ENSSSCG00000000902 | NR2C1 | 1.37E-14 | -0.111 |
| LW_ILN | MXE | ENSSSCG00000004134 | PEX3 | 0.000207 | 0.1 |
| LW_ILN | MXE | ENSSSCG00000007879 | NA | 3.98E-36 | 0.131 |
| LW_ILN | MXE | ENSSSCG00000020921 | USP33 | 0.002572 | -0.177 |
| LW_ILN | MXE | ENSSSCG00000011496 | ADAMTS9 | 2.03E-06 | -0.122 |
| LW_ILN | MXE | ENSSSCG00000007682 | SH2B2 | 0.039429 | 0.133 |
| LW_ILN | MXE | ENSSSCG00000015104 | TRAPPC4 | 0.003013 | 0.131 |
| LW_ILN | MXE | ENSSSCG00000000522 | NAP1L1 | 9.94E-06 | -0.152 |
| LW_ILN | MXE | ENSSSCG00000004364 | HACE1 | 0.006489 | 0.252 |
| LW_ILN | MXE | ENSSSCG00000010584 | MFSD13A | 0.002148 | -0.319 |
| LW_ILN | MXE | ENSSSCG00000015671 | NA | 1.02E-39 | -0.209 |
| LW_ILN | MXE | ENSSSCG00000005787 | NA | 5.57E-07 | 0.211 |
| LW_ILN | MXE | ENSSSCG00000005787 | NA | 5.8E-62 | 0.181 |
| LW_ILN | MXE | ENSSSCG00000005787 | NA | 0.04325 | 0.113 |
| LW_ILN | MXE | ENSSSCG00000002896 | NA | 1.4E-10 | -0.301 |
| LW_ILN | MXE | ENSSSCG00000002330 | PCNX | 0.002039 | -0.141 |
| LW_ILN | MXE | ENSSSCG00000024091 | ITGAD | 1.52E-09 | -0.41 |
| LW_ILN | MXE | ENSSSCG00000010097 | LZTR1 | 9.84E-05 | 0.133 |
| LW_ILN | MXE | ENSSSCG00000013601 | MARCH2 | 9.5E-11 | 0.134 |
| LW_ILN | MXE | ENSSSCG00000029212 | GDE1 | 1.09E-29 | 0.156 |
| LW_ILN | MXE | ENSSSCG00000011849 | TNK2 | 0.000211 | -0.137 |
| LW_ILN | MXE | ENSSSCG00000004044 | IGF2R | 0.007912 | 0.12 |
| LW_ILN | MXE | ENSSSCG00000010620 | XPNPEP1 | 0.000464 | 0.199 |
| LW_ILN | MXE | ENSSSCG00000014048 | HK3 | 0.002653 | 0.153 |
| LW_ILN | MXE | ENSSSCG00000014048 | HK3 | 0.002384 | 0.111 |
| LW_ILN | MXE | ENSSSCG00000000094 | SUN2 | 1.4E-15 | 0.111 |
| LW_ILN | MXE | ENSSSCG00000009396 | PHF11 | 4.91E-06 | -0.213 |
| LW_ILN | MXE | ENSSSCG00000015034 | SDHD | 0.000198 | -0.125 |
| LW_ILN | MXE | ENSSSCG00000003403 | APITD1 | 1.19E-06 | 0.129 |
| LW_ILN | MXE | ENSSSCG00000000683 | CDCA3 | 7.06E-11 | 0.142 |
| LW_ILN | MXE | ENSSSCG00000003652 | AKIRIN1 | 2.43E-17 | 0.141 |
| LW_ILN | MXE | ENSSSCG00000011895 | PLA1A | 0.001522 | -0.139 |
| LW_ILN | MXE | ENSSSCG00000006751 | SIKE1 | 3.39E-13 | 0.113 |
| LW_ILN | MXE | ENSSSCG00000023315 | THOC6 | 0.005418 | -0.131 |
| LW_ILN | MXE | ENSSSCG00000017280 | ICAM2 | 5.91E-15 | 0.125 |
| LW_ILN | MXE | ENSSSCG00000023894 | NA | 0.013434 | 0.114 |
| LW_ILN | MXE | ENSSSCG00000023204 | AXIN1 | 0.003131 | -0.105 |
| LW_ILN | MXE | ENSSSCG00000015933 | FASTKD1 | 0.003395 | -0.1 |
| LW_ILN | MXE | ENSSSCG00000010170 | DISC1 | 1.39E-11 | -0.108 |
| LW_ILN | MXE | ENSSSCG00000007133 | ACSS1 | 1.02E-58 | 0.197 |
| LW_ILN | MXE | ENSSSCG00000000704 | TAPBPL | 0.004496 | 0.125 |
| LW_ILN | MXE | ENSSSCG00000013556 | ADGRE1 | 0.046223 | -0.168 |
| LW_ILN | MXE | ENSSSCG00000007895 | ZC3H7A | 0.000487 | 0.102 |
| LW_ILN | MXE | ENSSSCG00000014060 | KIAA1191 | 0.033367 | 0.163 |
| LW_ILN | MXE | ENSSSCG00000014060 | KIAA1191 | 8.3E-17 | 0.141 |
| LW_ILN | MXE | ENSSSCG00000013322 | IMMP1L | 0.01797 | 0.149 |
| LW_ILN | MXE | ENSSSCG00000027677 | ABI3 | 1.33E-34 | -0.16 |
| LW_ILN | MXE | ENSSSCG00000003792 | LRRC40 | 0.004205 | -0.202 |
| LW_ILN | MXE | ENSSSCG00000008293 | DGUOK | 0.007109 | 0.136 |
| LW_ILN | MXE | ENSSSCG00000008293 | DGUOK | 4.06E-07 | 0.113 |
| LW_ILN | MXE | ENSSSCG00000008068 | NA | 4.77E-16 | -0.119 |
| LW_ILN | MXE | ENSSSCG00000017207 | NUP85 | 0.008947 | -0.164 |
| LW_ILN | MXE | ENSSSCG00000014882 | RSF1 | 9.69E-66 | 0.159 |
| LW_ILN | MXE | ENSSSCG00000009426 | CCDC122 | 1.24E-08 | -0.347 |
| LW_ILN | MXE | ENSSSCG00000002508 | SETD3 | 4.98E-16 | 0.102 |
| LW_ILN | MXE | ENSSSCG00000007609 | ARPC1A | 5.05E-06 | -0.143 |
| LW_ILN | MXE | ENSSSCG00000029196 | DIP2B | 1.14E-09 | 0.1 |
| LW_ILN | MXE | ENSSSCG00000006337 | HSD17B7 | 1.03E-17 | -0.105 |
| LW_ILN | MXE | ENSSSCG00000011857 | LMLN | 0.030045 | -0.256 |
| LW_ILN | MXE | ENSSSCG00000008300 | STAMBP | 0.022776 | 0.171 |
| LW_ILN | MXE | ENSSSCG00000016882 | PARP8 | 0.001799 | -0.135 |
| LW_ILN | MXE | ENSSSCG00000028260 | NA | 0.034016 | 0.1 |
| LW_ILN | MXE | ENSSSCG00000028260 | NA | 5.41E-13 | 0.2 |
| LW_ILN | MXE | ENSSSCG00000026106 | TXNDC15 | 3.19E-33 | 0.201 |
| LW_ILN | MXE | ENSSSCG00000012035 | SYNJ1 | 0.016634 | -0.105 |
| LW_ILN | MXE | ENSSSCG00000001970 | HEATR5A | 3.45E-62 | 0.185 |
| LW_ILN | MXE | ENSSSCG00000005286 | CEP78 | 0.004307 | -0.171 |
| LW_ILN | MXE | ENSSSCG00000028983 | NA | 5.16E-09 | 0.251 |
| LW_ILN | MXE | ENSSSCG00000014579 | NA | 0.000895 | -0.107 |
| LW_ILN | MXE | ENSSSCG00000004166 | AHI1 | 1.71E-78 | 0.174 |
| LW_ILN | MXE | ENSSSCG00000011446 | NEK4 | 0.026255 | 0.118 |
| LW_ILN | MXE | ENSSSCG00000002792 | HSF4 | 0.012693 | -0.159 |
| LW_ILN | MXE | ENSSSCG00000013906 | KXD1 | 1.44E-05 | -0.106 |
| LW_ILN | MXE | ENSSSCG00000014800 | RNF121 | 0.003472 | 0.165 |
| LW_ILN | MXE | ENSSSCG00000004382 | SEC63 | 8.42E-08 | 0.105 |
| LW_ILN | MXE | ENSSSCG00000008226 | POLR1A | 0.020946 | -0.104 |
| LW_ILN | MXE | ENSSSCG00000025186 | NA | 0.000388 | 0.126 |
| LW_ILN | MXE | ENSSSCG00000012742 | MTM1 | 0.010842 | -0.253 |
| LW_ILN | MXE | ENSSSCG00000012742 | MTM1 | 5.88E-44 | -0.166 |
| LW_ILN | MXE | ENSSSCG00000004725 | ZNF106 | 1.21E-24 | -0.142 |
| LW_ILN | MXE | ENSSSCG00000004020 | SFT2D1 | 1.22E-93 | 0.125 |
| LW_ILN | MXE | ENSSSCG00000010122 | CDC45 | 9.84E-08 | 0.179 |
| LW_ILN | MXE | ENSSSCG00000010122 | CDC45 | 3.04E-18 | -0.131 |
| LW_ILN | MXE | ENSSSCG00000027410 | CBFA2T2 | 3.63E-08 | -0.114 |
| LW_ILN | MXE | ENSSSCG00000022011 | NMI | 1.63E-22 | -0.115 |
| LW_ILN | MXE | ENSSSCG00000011064 | ACBD5 | 2.05E-16 | -0.13 |
| LW_ILN | MXE | ENSSSCG00000009492 | GPR180 | 0.001604 | -0.136 |
| LW_ILN | MXE | ENSSSCG00000013764 | RFX1 | 1.16E-14 | 0.231 |
| LW_ILN | MXE | ENSSSCG00000012149 | RBBP7 | 0.000679 | 0.112 |
| LW_ILN | MXE | ENSSSCG00000006252 | NA | 0.001173 | 0.132 |
| LW_ILN | MXE | ENSSSCG00000006529 | SLC50A1 | 0.000278 | 0.115 |
| LW_ILN | MXE | ENSSSCG00000006912 | HFM1 | 4.05E-10 | 0.137 |
| LW_ILN | MXE | ENSSSCG00000030408 | DDX58 | 0.0003 | -0.103 |
| LW_ILN | MXE | ENSSSCG00000027374 | C1orf50 | 7.78E-25 | 0.135 |
| LW_ILN | MXE | ENSSSCG00000015476 | CHI3L1 | 2.31E-18 | -0.196 |
| LW_ILN | MXE | ENSSSCG00000007454 | ZMYND8 | 0.005631 | -0.104 |
| LW_ILN | MXE | ENSSSCG00000014431 | AFAP1L1 | 9.92E-23 | -0.226 |
| LW_ILN | MXE | ENSSSCG00000008845 | SRD5A3 | 0.03248 | 0.125 |
| LW_ILN | MXE | ENSSSCG00000013638 | ILF3 | 9.32E-07 | -0.111 |
| LW_ILN | MXE | ENSSSCG00000015588 | ANGEL2 | 1.84E-22 | 0.141 |
| LW_ILN | MXE | ENSSSCG00000006864 | CDC14A | 2.43E-05 | 0.222 |
| LW_ILN | MXE | ENSSSCG00000012489 | TRMT2B | 0.015467 | -0.373 |
| LW_ILN | RI | ENSSSCG00000022107 | CHTOP | 1.47E-34 | -0.143 |
| LW_ILN | RI | ENSSSCG00000006452 | CD1D | 2.7E-13 | -0.22 |
| LW_ILN | RI | ENSSSCG00000012173 | SAT1 | 1.31E-87 | -0.144 |
| LW_ILN | RI | ENSSSCG00000009585 | GADD45G | 2.06E-41 | 0.143 |
| LW_ILN | RI | ENSSSCG00000010212 | ANK3 | 2.68E-15 | -0.238 |
| LW_ILN | SE | ENSSSCG00000002266 | CHD2 | 3.14E-23 | -0.125 |
| LW_ILN | SE | ENSSSCG00000025248 | NA | 1.56E-36 | -0.139 |
| LW_ILN | SE | ENSSSCG00000011518 | SHQ1 | 0.038 | 0.135 |
| LW_ILN | SE | ENSSSCG00000028724 | NA | 7.36E-10 | -0.122 |
| LW_ILN | SE | ENSSSCG00000012944 | PELI3 | 0.009163 | -0.174 |
| LW_ILN | SE | ENSSSCG00000006508 | NA | 9.78E-05 | 0.123 |
| LW_ILN | SE | ENSSSCG00000006871 | SLC35A3 | 1.18E-06 | -0.147 |
| LW_ILN | SE | ENSSSCG00000007925 | NAGPA | 1.42E-30 | 0.179 |
| LW_ILN | SE | ENSSSCG00000001929 | PARP6 | 0.021455 | 0.224 |
| LW_ILN | SE | ENSSSCG00000007309 | RBM39 | 0 | -0.269 |
| LW_ILN | SE | ENSSSCG00000009075 | MFSD8 | 0.000148 | -0.14 |
| LW_ILN | SE | ENSSSCG00000012118 | TLR8 | 0.000398 | 0.214 |
| LW_ILN | SE | ENSSSCG00000008237 | RETSAT | 0.00196 | -0.111 |
| LW_ILN | SE | ENSSSCG00000013836 | WIZ | 5.39E-74 | -0.284 |
| LW_ILN | SE | ENSSSCG00000000529 | NA | 3.92E-29 | -0.192 |
| LW_ILN | SE | ENSSSCG00000005060 | KTN1 | 0.016226 | 0.139 |
| LW_ILN | SE | ENSSSCG00000005060 | KTN1 | 7.96E-12 | -0.153 |
| LW_ILN | SE | ENSSSCG00000008354 | C1D | 1.21E-13 | 0.115 |
| LW_ILN | SE | ENSSSCG00000013409 | NA | 6.23E-17 | -0.125 |
| LW_ILN | SE | ENSSSCG00000006470 | RRNAD1 | 5.59E-05 | -0.304 |
| LW_ILN | SE | ENSSSCG00000003580 | EYA3 | 2.19E-11 | 0.13 |
| LW_ILN | SE | ENSSSCG00000007855 | NA | 1.28E-21 | -0.166 |
| LW_ILN | SE | ENSSSCG00000006457 | FCRL3 | 0.027443 | -0.156 |
| LW_ILN | SE | ENSSSCG00000006457 | FCRL3 | 8.6E-121 | -0.489 |
| LW_ILN | SE | ENSSSCG00000006457 | FCRL3 | 2.65E-28 | -0.14 |
| LW_ILN | SE | ENSSSCG00000006457 | FCRL3 | 3.25E-38 | -0.142 |
| LW_ILN | SE | ENSSSCG00000006066 | RNF19A | 0.0356 | 0.123 |
| LW_ILN | SE | ENSSSCG00000010987 | UBAP2 | 0.000221 | 0.253 |
| LW_ILN | SE | ENSSSCG00000025252 | PEX5 | 8.85E-08 | 0.307 |
| LW_ILN | SE | ENSSSCG00000008684 | POLN | 0.000124 | 0.138 |
| LW_ILN | SE | ENSSSCG00000007200 | FAM110A | 0.029167 | -0.138 |
| LW_ILN | SE | ENSSSCG00000012659 | ZNF280C | 0.002286 | 0.218 |
| LW_ILN | SE | ENSSSCG00000001457 | SLA-DQB1 | 1.79E-56 | -0.106 |
| LW_ILN | SE | ENSSSCG00000027252 | HCFC1R1 | 8.16E-05 | -0.226 |
| LW_ILN | SE | ENSSSCG00000014276 | SLC22A5 | 1.69E-07 | 0.146 |
| LW_ILN | SE | ENSSSCG00000028377 | CRYZL1 | 0.000335 | -0.111 |
| LW_ILN | SE | ENSSSCG00000016101 | CFLAR | 7.2E-19 | 0.159 |
| LW_ILN | SE | ENSSSCG00000006000 | TAF2 | 1.97E-10 | 0.269 |
| LW_ILN | SE | ENSSSCG00000025114 | NA | 0.025875 | 0.173 |
| LW_ILN | SE | ENSSSCG00000000687 | CD4 | 0.031579 | -0.149 |
| LW_ILN | SE | ENSSSCG00000014015 | C5orf45 | 1.32E-08 | -0.265 |
| LW_ILN | SE | ENSSSCG00000024872 | CPT1C | 6.64E-05 | -0.1 |
| LW_ILN | SE | ENSSSCG00000009744 | SFSWAP | 1.94E-13 | -0.141 |
| LW_ILN | SE | ENSSSCG00000001731 | CENPQ | 0.006431 | 0.38 |
| LW_ILN | SE | ENSSSCG00000006827 | CYB561D1 | 1.37E-06 | 0.106 |
| LW_ILN | SE | ENSSSCG00000012063 | DSCR3 | 0.000102 | 0.199 |
| LW_ILN | SE | ENSSSCG00000026924 | KIFC2 | 0.027155 | -0.169 |
| LW_ILN | SE | ENSSSCG00000011026 | ARHGAP12 | 0.002902 | 0.176 |
| LW_ILN | SE | ENSSSCG00000003848 | LRP8 | 0.000601 | 0.239 |
| LW_ILN | SE | ENSSSCG00000003848 | LRP8 | 4.22E-18 | 0.403 |
| LW_ILN | SE | ENSSSCG00000003848 | LRP8 | 1.14E-06 | -0.256 |
| LW_ILN | SE | ENSSSCG00000006350 | FCGR2B | 5.9E-164 | 0.459 |
| LW_ILN | SE | ENSSSCG00000008881 | RAPGEF2 | 0.009169 | -0.128 |
| LW_ILN | SE | ENSSSCG00000026473 | NA | 9.27E-05 | -0.174 |
| LW_ILN | SE | ENSSSCG00000004408 | SMPD2 | 0.001119 | 0.188 |
| LW_ILN | SE | ENSSSCG00000024564 | IMMT | 8.05E-12 | -0.115 |
| LW_ILN | SE | ENSSSCG00000000265 | ESPL1 | 1.09E-09 | 0.109 |
| LW_ILN | SE | ENSSSCG00000014868 | EMSY | 7.71E-05 | 0.242 |
| LW_ILN | SE | ENSSSCG00000014868 | EMSY | 7.55E-05 | -0.148 |
| LW_ILN | SE | ENSSSCG00000015947 | DCAF17 | 5.75E-09 | 0.198 |
| LW_ILN | SE | ENSSSCG00000015947 | DCAF17 | 5.1E-09 | 0.286 |
| LW_ILN | SE | ENSSSCG00000011041 | NSUN6 | 1.95E-11 | -0.146 |
| LW_ILN | SE | ENSSSCG00000011075 | KIAA1217 | 4.13E-08 | -0.221 |
| LW_ILN | SE | ENSSSCG00000023269 | EXOC1 | 1.02E-19 | 0.174 |
| LW_ILN | SE | ENSSSCG00000013857 | EPS15L1 | 0.002897 | 0.117 |
| LW_ILN | SE | ENSSSCG00000003396 | NA | 7.6E-20 | -0.177 |
| LW_ILN | SE | ENSSSCG00000013857 | EPS15L1 | 6.56E-40 | -0.312 |
| LW_ILN | SE | ENSSSCG00000003784 | LRRIQ3 | 6.63E-80 | -0.237 |
| LW_ILN | SE | ENSSSCG00000013775 | ADGRE5 | 4.27E-17 | 0.136 |
| LW_ILN | SE | ENSSSCG00000028516 | LARP4 | 1.8E-10 | 0.119 |
| LW_ILN | SE | ENSSSCG00000015883 | MARCH7 | 1.6E-292 | -0.175 |
| LW_ILN | SE | ENSSSCG00000014304 | SEC24A | 0.016485 | 0.12 |
| LW_ILN | SE | ENSSSCG00000024628 | ALKBH8 | 1.76E-07 | -0.101 |
| LW_ILN | SE | ENSSSCG00000024628 | ALKBH8 | 6.19E-13 | -0.153 |
| LW_ILN | SE | ENSSSCG00000009334 | HSPH1 | 1.7E-230 | -0.33 |
| LW_ILN | SE | ENSSSCG00000005699 | HMCN2 | 6.48E-24 | -0.136 |
| LW_ILN | SE | ENSSSCG00000015878 | NA | 3.53E-27 | -0.47 |
| LW_ILN | SE | ENSSSCG00000005623 | ST6GALNAC6 | 0.001823 | -0.323 |
| LW_ILN | SE | ENSSSCG00000015720 | BIN1 | 2.99E-14 | -0.248 |
| LW_ILN | SE | ENSSSCG00000010316 | KAT6B | 0.021008 | -0.347 |
| LW_ILN | SE | ENSSSCG00000015908 | NA | 7.5E-08 | -0.652 |
| LW_ILN | SE | ENSSSCG00000012262 | KDM6A | 2.57E-11 | 0.189 |
| LW_ILN | SE | ENSSSCG00000000925 | C12orf29 | 0.048303 | -0.161 |
| LW_ILN | SE | ENSSSCG00000002341 | PAPLN | 0.009454 | -0.131 |
| LW_ILN | SE | ENSSSCG00000029349 | NA | 0.001124 | -0.223 |
| LW_ILN | SE | ENSSSCG00000027446 | NA | 6.87E-56 | -0.216 |
| LW_ILN | SE | ENSSSCG00000007930 | MGRN1 | 0.001182 | -0.239 |
| LW_ILN | SE | ENSSSCG00000026940 | CASP10 | 0.000639 | 0.152 |
| LW_ILN | SE | ENSSSCG00000024481 | NA | 0.005049 | 0.108 |
| LW_ILN | SE | ENSSSCG00000025181 | NA | 2.71E-05 | 0.105 |
| LW_ILN | SE | ENSSSCG00000008297 | TPRKB | 0.000427 | -0.231 |
| LW_ILN | SE | ENSSSCG00000008337 | AAK1 | 4.47E-06 | -0.125 |
| LW_ILN | SE | ENSSSCG00000017177 | ST6GALNAC2 | 9.2E-12 | -0.126 |
| LW_ILN | SE | ENSSSCG00000014394 | RNF14 | 4.44E-26 | -0.139 |
| LW_ILN | SE | ENSSSCG00000008202 | CNNM4 | 1.8E-13 | -0.18 |
| LW_ILN | SE | ENSSSCG00000009890 | MAPKAPK5 | 0.00473 | 0.104 |
| LW_ILN | SE | ENSSSCG00000024201 | FAM208A | 2.72E-35 | -0.215 |
| LW_ILN | SE | ENSSSCG00000015638 | C1orf186 | 3.8E-11 | 0.292 |
| LW_ILN | SE | ENSSSCG00000014240 | CSNK1G3 | 3.5E-23 | -0.242 |
| LW_ILN | SE | ENSSSCG00000021467 | EME2 | 1.97E-05 | 0.284 |
| LW_ILN | SE | ENSSSCG00000016567 | STRIP2 | 0.010325 | 0.163 |
| LW_ILN | SE | ENSSSCG00000029776 | FAM219B | 5.39E-13 | -0.164 |
| LW_ILN | SE | ENSSSCG00000009967 | PITPNB | 2.08E-10 | -0.103 |
| LW_ILN | SE | ENSSSCG00000016395 | RIF1 | 1.23E-69 | -0.195 |
| LW_ILN | SE | ENSSSCG00000004466 | TTK | 6.05E-60 | 0.317 |
| LW_ILN | SE | ENSSSCG00000012521 | GPRASP2 | 7.64E-06 | -0.115 |
| LW_ILN | SE | ENSSSCG00000011741 | GOLIM4 | 3.1E-26 | -0.158 |
| LW_ILN | SE | ENSSSCG00000012586 | ALG13 | 7.96E-08 | 0.104 |
| LW_ILN | SE | ENSSSCG00000011350 | CCDC51 | 3.62E-19 | -0.171 |
| LW_ILN | SE | ENSSSCG00000016214 | STK16 | 8.27E-05 | -0.128 |
| LW_ILN | SE | ENSSSCG00000002429 | FOXN3 | 1.78E-11 | -0.142 |
| LW_ILN | SE | ENSSSCG00000004729 | GANC | 9.41E-06 | -0.363 |
| LW_ILN | SE | ENSSSCG00000015294 | CR1 | 9.14E-13 | 0.233 |
| LW_ILN | SE | ENSSSCG00000023296 | CENPE | 2.99E-07 | 0.181 |
| LW_ILN | SE | ENSSSCG00000016954 | ERBB2IP | 0.035754 | -0.108 |
| LW_ILN | SE | ENSSSCG00000012586 | ALG13 | 9.77E-08 | 0.113 |
| LW_ILN | SE | ENSSSCG00000000966 | SCO2 | 5.44E-22 | 0.139 |
| LW_ILN | SE | ENSSSCG00000026996 | NA | 0.01461 | 0.152 |
| LW_ILN | SE | ENSSSCG00000013788 | NA | 0.008885 | -0.122 |
| LW_ILN | SE | ENSSSCG00000008040 | TSC2 | 1.21E-38 | -0.274 |
| LW_ILN | SE | ENSSSCG00000005173 | NA | 6.32E-93 | 0.293 |
| LW_ILN | SE | ENSSSCG00000009142 | SEC24B | 0.013916 | 0.109 |
| LW_ILN | SE | ENSSSCG00000009230 | WDFY3 | 5.16E-06 | -0.165 |
| LW_ILN | SE | ENSSSCG00000026218 | FIP1L1 | 3.81E-05 | -0.247 |
| LW_ILN | SE | ENSSSCG00000012649 | SMARCA1 | 0.002441 | 0.103 |
| LW_ILN | SE | ENSSSCG00000010654 | ATRNL1 | 1.48E-12 | -0.157 |
| LW_ILN | SE | ENSSSCG00000029482 | CHST15 | 0.036659 | -0.101 |
| LW_ILN | SE | ENSSSCG00000010519 | NA | 0.040122 | -0.242 |
| LW_ILN | SE | ENSSSCG00000011317 | FYCO1 | 1.4E-26 | -0.103 |
| LW_ILN | SE | ENSSSCG00000025290 | ZNF596 | 3.66E-07 | -0.166 |
| LW_ILN | SE | ENSSSCG00000013622 | TMEM205 | 0.002555 | 0.102 |
| LW_ILN | SE | ENSSSCG00000015586 | RPS6KC1 | 1.72E-09 | 0.136 |
| LW_ILN | SE | ENSSSCG00000023801 | LANCL2 | 7.58E-23 | 0.312 |
| LW_ILN | SE | ENSSSCG00000022754 | SLC39A3 | 3.95E-43 | 0.196 |
| LW_ILN | SE | ENSSSCG00000023801 | LANCL2 | 8.03E-11 | 0.193 |
| LW_ILN | SE | ENSSSCG00000013174 | CTNND1 | 5.29E-35 | 0.12 |
| LW_ILN | SE | ENSSSCG00000007999 | NA | 3E-29 | -0.321 |
| LW_ILN | SE | ENSSSCG00000012912 | TBC1D10C | 1.83E-38 | -0.129 |
| LW_ILN | SE | ENSSSCG00000030229 | ABHD14A | 0.000297 | -0.119 |
| LW_ILN | SE | ENSSSCG00000000681 | P3H3 | 2.51E-09 | -0.372 |
| LW_ILN | SE | ENSSSCG00000013658 | S1PR2 | 9.35E-21 | 0.17 |
| LW_ILN | SE | ENSSSCG00000006461 | ARHGEF11 | 0.000345 | 0.636 |
| LW_ILN | SE | ENSSSCG00000030255 | EIF4G1 | 0.025227 | 0.236 |
| LW_ILN | SE | ENSSSCG00000005782 | TM2D3 | 1.21E-13 | -0.18 |
| LW_ILN | SE | ENSSSCG00000030791 | DOCK11 | 4.31E-07 | -0.147 |
| LW_ILN | SE | ENSSSCG00000009139 | PLA2G12A | 0.012621 | -0.243 |
| LW_ILN | SE | ENSSSCG00000010118 | HIRA | 1.21E-09 | 0.157 |
| LW_ILN | SE | ENSSSCG00000014370 | TMCO6 | 0.042341 | 0.132 |
| LW_ILN | SE | ENSSSCG00000014141 | RFESD | 4.28E-07 | 0.185 |
| LW_ILN | SE | ENSSSCG00000015885 | PLA2R1 | 1.71E-14 | -0.161 |
| LW_ILN | SE | ENSSSCG00000010541 | COX15 | 3.95E-67 | 0.259 |
| LW_ILN | SE | ENSSSCG00000028018 | EP400 | 0.032263 | -0.115 |
| LW_ILN | SE | ENSSSCG00000014562 | NA | 6.96E-89 | 0.184 |
| LW_ILN | SE | ENSSSCG00000005658 | ZDHHC12 | 3.03E-06 | -0.181 |
| LW_ILN | SE | ENSSSCG00000004306 | ORC3 | 0.005844 | -0.162 |
| LW_ILN | SE | ENSSSCG00000001242 | GABBR1 | 0.000764 | -0.185 |
| LW_ILN | SE | ENSSSCG00000008262 | SEMA4F | 0.028212 | 0.253 |
| LW_ILN | SE | ENSSSCG00000009244 | SEC31A | 7.93E-17 | 0.158 |
| LW_ILN | SE | ENSSSCG00000015965 | GPR155 | 0.013393 | -0.125 |
| LW_ILN | SE | ENSSSCG00000028964 | PIM2 | 0.023594 | 0.114 |
| LW_ILN | SE | ENSSSCG00000016864 | C5orf51 | 0.00025 | -0.16 |
| LW_ILN | SE | ENSSSCG00000005465 | SUSD1 | 5.19E-38 | -0.315 |
| LW_ILN | SE | ENSSSCG00000000739 | FOXM1 | 0.007225 | 0.131 |
| LW_ILN | SE | ENSSSCG00000008177 | NA | 8.86E-06 | 0.144 |
| LW_ILN | SE | ENSSSCG00000014207 | APC | 0.001777 | -0.156 |
| LW_ILN | SE | ENSSSCG00000018049 | NA | 0.009596 | -0.325 |
| LW_ILN | SE | ENSSSCG00000000702 | ZNF384 | 4.73E-05 | -0.276 |
| LW_ILN | SE | ENSSSCG00000002896 | NA | 4.53E-12 | 0.256 |
| LW_ILN | SE | ENSSSCG00000002896 | NA | 0.032089 | 0.206 |
| LW_ILN | SE | ENSSSCG00000002896 | NA | 5.46E-22 | -0.234 |
| LW_ILN | SE | ENSSSCG00000016831 | SPEF2 | 5.28E-06 | -0.332 |
| LW_ILN | SE | ENSSSCG00000011379 | KLHDC8B | 3.22E-07 | -0.11 |
| LW_ILN | SE | ENSSSCG00000001382 | DDR1 | 0.004939 | -0.328 |
| LW_ILN | SE | ENSSSCG00000024361 | TFB1M | 1.61E-20 | 0.113 |
| LW_ILN | SE | ENSSSCG00000013878 | PGLS | 9.38E-19 | -0.1 |
| LW_ILN | SE | ENSSSCG00000007830 | RBBP6 | 0.042592 | -0.104 |
| LW_ILN | SE | ENSSSCG00000004745 | RPAP1 | 1.01E-16 | 0.102 |
| LW_ILN | SE | ENSSSCG00000015671 | NA | 2.24E-14 | 0.268 |
| LW_ILN | SE | ENSSSCG00000007520 | GNAS | 4.07E-21 | 0.201 |
| LW_ILN | SE | ENSSSCG00000007520 | GNAS | 0.000271 | -0.24 |
| LW_ILN | SE | ENSSSCG00000008842 | KIT | 0.001529 | -0.291 |
| LW_ILN | SE | ENSSSCG00000002426 | ZC3H14 | 8.19E-07 | -0.119 |
| LW_ILN | SE | ENSSSCG00000028347 | PPP1R11 | 1.14E-05 | -0.144 |
| LW_ILN | SE | ENSSSCG00000010850 | ENAH | 8.58E-06 | 0.144 |
| LW_ILN | SE | ENSSSCG00000009356 | RFXAP | 4.38E-12 | 0.128 |
| LW_ILN | SE | ENSSSCG00000027052 | PEX11A | 0.000599 | 0.277 |
| LW_ILN | SE | ENSSSCG00000030431 | RANGRF | 0.002735 | -0.119 |
| LW_ILN | SE | ENSSSCG00000011849 | TNK2 | 0.000331 | -0.164 |
| LW_ILN | SE | ENSSSCG00000000753 | WNK1 | 1.3E-14 | 0.23 |
| LW_ILN | SE | ENSSSCG00000009975 | EMID1 | 8.27E-06 | -0.154 |
| LW_ILN | SE | ENSSSCG00000005725 | TTF1 | 0.037286 | 0.12 |
| LW_ILN | SE | ENSSSCG00000011555 | RPUSD3 | 2.09E-32 | -0.223 |
| LW_ILN | SE | ENSSSCG00000002142 | TEP1 | 0.041877 | -0.131 |
| LW_ILN | SE | ENSSSCG00000014048 | HK3 | 5.89E-05 | -0.219 |
| LW_ILN | SE | ENSSSCG00000014048 | HK3 | 1.63E-07 | -0.28 |
| LW_ILN | SE | ENSSSCG00000028096 | NA | 1.57E-26 | -0.14 |
| LW_ILN | SE | ENSSSCG00000028096 | NA | 0.00028 | -0.221 |
| LW_ILN | SE | ENSSSCG00000015815 | FGFR1 | 1.48E-59 | 0.17 |
| LW_ILN | SE | ENSSSCG00000016473 | NA | 5.97E-05 | -0.111 |
| LW_ILN | SE | ENSSSCG00000006751 | SIKE1 | 2.03E-06 | -0.175 |
| LW_ILN | SE | ENSSSCG00000000697 | CHD4 | 0.00902 | -0.14 |
| LW_ILN | SE | ENSSSCG00000013257 | ATG13 | 6.78E-05 | 0.15 |
| LW_ILN | SE | ENSSSCG00000008697 | HTT | 9.41E-06 | -0.149 |
| LW_ILN | SE | ENSSSCG00000001881 | MAN2C1 | 2.33E-37 | -0.361 |
| LW_ILN | SE | ENSSSCG00000001881 | MAN2C1 | 1.06E-06 | -0.311 |
| LW_ILN | SE | ENSSSCG00000000705 | CD27 | 4.03E-07 | -0.681 |
| LW_ILN | SE | ENSSSCG00000017638 | BZRAP1 | 2.09E-08 | -0.174 |
| LW_ILN | SE | ENSSSCG00000014211 | YTHDC2 | 0.03868 | 0.135 |
| LW_ILN | SE | ENSSSCG00000005191 | MPDZ | 4.42E-13 | -0.16 |
| LW_ILN | SE | ENSSSCG00000004888 | SERPINB8 | 0.041851 | -0.273 |
| LW_ILN | SE | ENSSSCG00000004163 | BCLAF1 | 4.13E-12 | -0.117 |
| LW_ILN | SE | ENSSSCG00000027331 | COL6A3 | 9.44E-05 | -0.202 |
| LW_ILN | SE | ENSSSCG00000015310 | AKAP9 | 2.62E-22 | -0.159 |
| LW_ILN | SE | ENSSSCG00000029573 | NASP | 0.004194 | 0.101 |
| LW_ILN | SE | ENSSSCG00000010774 | ZNF511 | 0.022524 | -0.119 |
| LW_ILN | SE | ENSSSCG00000013755 | CCDC130 | 0.000855 | -0.549 |
| LW_ILN | SE | ENSSSCG00000009978 | AP1B1 | 1.56E-06 | -0.128 |
| LW_ILN | SE | ENSSSCG00000030284 | TRNAU1AP | 4.64E-69 | -0.438 |
| LW_ILN | SE | ENSSSCG00000016174 | FN1 | 1.78E-06 | -0.172 |
| LW_ILN | SE | ENSSSCG00000001970 | HEATR5A | 2.23E-34 | -0.131 |
| LW_ILN | SE | ENSSSCG00000005950 | PHF20L1 | 0.015166 | 0.178 |
| LW_ILN | SE | ENSSSCG00000001970 | HEATR5A | 0.016438 | -0.168 |
| LW_ILN | SE | ENSSSCG00000017099 | FASTKD3 | 6.87E-07 | 0.112 |
| LW_ILN | SE | ENSSSCG00000013322 | IMMP1L | 0.003461 | -0.273 |
| LW_ILN | SE | ENSSSCG00000013322 | IMMP1L | 8.18E-28 | -0.181 |
| LW_ILN | SE | ENSSSCG00000013322 | IMMP1L | 0.006119 | 0.15 |
| LW_ILN | SE | ENSSSCG00000013322 | IMMP1L | 1.45E-32 | 0.165 |
| LW_ILN | SE | ENSSSCG00000009771 | DDX55 | 1.79E-08 | 0.194 |
| LW_ILN | SE | ENSSSCG00000022385 | BTBD8 | 0.001542 | 0.186 |
| LW_ILN | SE | ENSSSCG00000011882 | GOLGB1 | 1.94E-05 | 0.108 |
| LW_ILN | SE | ENSSSCG00000028674 | CR2 | 2.2E-153 | 0.17 |
| LW_ILN | SE | ENSSSCG00000006303 | NA | 8.8E-19 | 0.338 |
| LW_ILN | SE | ENSSSCG00000006303 | NA | 0.018045 | 0.227 |
| LW_ILN | SE | ENSSSCG00000014909 | SYTL2 | 8.09E-05 | 0.301 |
| LW_ILN | SE | ENSSSCG00000009564 | TMEM255B | 1.69E-17 | -0.239 |
| LW_ILN | SE | ENSSSCG00000026110 | SRPK2 | 3.52E-10 | -0.261 |
| LW_ILN | SE | ENSSSCG00000009048 | GAB1 | 3.8E-16 | 0.443 |
| LW_ILN | SE | ENSSSCG00000004901 | PIGN | 0.005646 | -0.219 |
| LW_ILN | SE | ENSSSCG00000017360 | UBTF | 2.81E-07 | -0.183 |
| LW_ILN | SE | ENSSSCG00000017360 | UBTF | 1.85E-28 | 0.219 |
| LW_ILN | SE | ENSSSCG00000004901 | PIGN | 2.8E-27 | -0.142 |
| LW_ILN | SE | ENSSSCG00000004901 | PIGN | 0.00114 | -0.228 |
| LW_ILN | SE | ENSSSCG00000010604 | SH3PXD2A | 0.011639 | 0.412 |
| LW_ILN | SE | ENSSSCG00000008293 | DGUOK | 0.00063 | 0.16 |
| LW_ILN | SE | ENSSSCG00000025866 | PRX | 8.43E-22 | -0.393 |
| LW_ILN | SE | ENSSSCG00000010055 | GGT5 | 6.06E-14 | -0.282 |
| LW_ILN | SE | ENSSSCG00000017748 | NF1 | 2E-100 | -0.195 |
| LW_ILN | SE | ENSSSCG00000009151 | CYP2U1 | 1.6E-05 | -0.139 |
| LW_ILN | SE | ENSSSCG00000001822 | RCCD1 | 1.18E-05 | -0.202 |
| LW_ILN | SE | ENSSSCG00000006161 | IL7 | 0.007632 | -0.13 |
| LW_ILN | SE | ENSSSCG00000008185 | MITD1 | 1.11E-11 | -0.125 |
| LW_ILN | SE | ENSSSCG00000004516 | MBD1 | 2.59E-20 | -0.208 |
| LW_ILN | SE | ENSSSCG00000009426 | CCDC122 | 0.042944 | -0.186 |
| LW_ILN | SE | ENSSSCG00000030520 | NA | 1.41E-07 | -0.129 |
| LW_ILN | SE | ENSSSCG00000009583 | NA | 1.63E-18 | 0.215 |
| LW_ILN | SE | ENSSSCG00000007252 | DNMT3B | 0.002826 | -0.106 |
| LW_ILN | SE | ENSSSCG00000009745 | ADGRD1 | 7.39E-15 | 0.383 |
| LW_ILN | SE | ENSSSCG00000006337 | HSD17B7 | 4.81E-20 | 0.122 |
| LW_ILN | SE | ENSSSCG00000011393 | UBA7 | 4.02E-12 | -0.154 |
| LW_ILN | SE | ENSSSCG00000000968 | SBF1 | 7.21E-29 | -0.13 |
| LW_ILN | SE | ENSSSCG00000021075 | TDRD1 | 0.0253 | 0.213 |
| LW_ILN | SE | ENSSSCG00000012123 | RAB9A | 3E-05 | 0.12 |
| LW_ILN | SE | ENSSSCG00000012123 | RAB9A | 3.28E-06 | 0.153 |
| LW_ILN | SE | ENSSSCG00000022839 | NA | 4.33E-10 | 0.193 |
| LW_ILN | SE | ENSSSCG00000000953 | NA | 8.32E-06 | -0.165 |
| LW_ILN | SE | ENSSSCG00000005367 | TSTD2 | 2.44E-68 | -0.229 |
| LW_ILN | SE | ENSSSCG00000008392 | BCL11A | 3.62E-13 | 0.113 |
| LW_ILN | SE | ENSSSCG00000005717 | UCK1 | 6.89E-38 | -0.215 |
| LW_ILN | SE | ENSSSCG00000021714 | PRR3 | 2.01E-05 | 0.255 |
| LW_ILN | SE | ENSSSCG00000002368 | LTBP2 | 0.031991 | 0.209 |
| LW_ILN | SE | ENSSSCG00000011632 | NA | 0.002299 | -0.154 |
| LW_ILN | SE | ENSSSCG00000024315 | NA | 8.05E-14 | -0.111 |
| LW_ILN | SE | ENSSSCG00000010943 | NA | 0.01792 | 0.121 |
| LW_ILN | SE | ENSSSCG00000004701 | CATSPER2 | 2.33E-07 | 0.17 |
| LW_ILN | SE | ENSSSCG00000015948 | DYNC1I2 | 1.24E-68 | -0.169 |
| LW_ILN | SE | ENSSSCG00000015853 | NA | 1.37E-26 | -0.252 |
| LW_ILN | SE | ENSSSCG00000000648 | CLEC7A | 6.53E-07 | -0.168 |
| LW_ILN | SE | ENSSSCG00000017794 | EFCAB5 | 1.88E-14 | -0.468 |
| LW_ILN | SE | ENSSSCG00000011498 | SLC25A26 | 0.012794 | 0.169 |
| LW_ILN | SE | ENSSSCG00000004698 | SERINC4 | 2.08E-11 | 0.169 |
| LW_ILN | SE | ENSSSCG00000004166 | AHI1 | 0.001231 | -0.333 |
| LW_ILN | SE | ENSSSCG00000004832 | UBE3A | 1.43E-05 | -0.155 |
| LW_ILN | SE | ENSSSCG00000004832 | UBE3A | 0.000896 | -0.366 |
| LW_ILN | SE | ENSSSCG00000030211 | NBR1 | 9.67E-46 | -0.106 |
| LW_ILN | SE | ENSSSCG00000005987 | NA | 0.000405 | 0.118 |
| LW_ILN | SE | ENSSSCG00000014204 | DCP2 | 4.75E-11 | 0.423 |
| LW_ILN | SE | ENSSSCG00000006340 | UAP1 | 2.64E-13 | -0.131 |
| LW_ILN | SE | ENSSSCG00000007108 | KIZ | 7.31E-05 | 0.238 |
| LW_ILN | SE | ENSSSCG00000004709 | ZSCAN29 | 1.45E-26 | 0.116 |
| LW_ILN | SE | ENSSSCG00000000588 | PLEKHA5 | 2.35E-05 | -0.195 |
| LW_ILN | SE | ENSSSCG00000023419 | NA | 1.39E-05 | -0.201 |
| LW_ILN | SE | ENSSSCG00000010251 | SUPV3L1 | 0.002654 | -0.13 |
| LW_ILN | SE | ENSSSCG00000024254 | ALKBH7 | 9.97E-29 | -0.167 |
| LW_ILN | SE | ENSSSCG00000004725 | ZNF106 | 0.015255 | 0.135 |
| LW_ILN | SE | ENSSSCG00000004997 | PRPF39 | 0.00493 | -0.146 |
| LW_ILN | SE | ENSSSCG00000029219 | SH3BGR | 3.84E-24 | -0.481 |
| LW_ILN | SE | ENSSSCG00000017100 | MTRR | 0.0475 | 0.156 |
| LW_ILN | SE | ENSSSCG00000028686 | NA | 1.91E-07 | 0.167 |
| LW_ILN | SE | ENSSSCG00000015492 | DARS2 | 5.34E-29 | 0.325 |
| LW_ILN | SE | ENSSSCG00000025564 | GAS2L1 | 3.55E-12 | -0.122 |
| LW_ILN | SE | ENSSSCG00000024006 | NA | 2.85E-05 | 0.139 |
| LW_ILN | SE | ENSSSCG00000010212 | ANK3 | 0.026678 | -0.343 |
| LW_ILN | SE | ENSSSCG00000012241 | BCOR | 0.001514 | -0.194 |
| LW_ILN | SE | ENSSSCG00000011301 | ZDHHC3 | 5.53E-60 | -0.168 |
| LW_ILN | SE | ENSSSCG00000006912 | HFM1 | 1.14E-07 | -0.401 |
| LW_ILN | SE | ENSSSCG00000006912 | HFM1 | 5.46E-44 | 0.474 |
| LW_ILN | SE | ENSSSCG00000004969 | KIF23 | 0.002052 | -0.206 |
| LW_ILN | SE | ENSSSCG00000011553 | NA | 0.010771 | -0.119 |
| LW_ILN | SE | ENSSSCG00000023304 | SRRM2 | 8.08E-26 | -0.153 |
| LW_ILN | SE | ENSSSCG00000014817 | ARAP1 | 0.044583 | -0.17 |
| LW_ILN | SE | ENSSSCG00000013588 | MAP2K7 | 3.02E-11 | -0.144 |
| LW_ILN | SE | ENSSSCG00000010130 | GNB1L | 1.22E-14 | 0.148 |
| LW_ILN | SE | ENSSSCG00000010600 | CALHM2 | 2.64E-06 | 0.142 |
| LW_ILN | SE | ENSSSCG00000004123 | NA | 0.00697 | -0.294 |
| LW_ILN | SE | ENSSSCG00000011258 | EXOG | 0.013631 | 0.169 |
| LW_ILN | SE | ENSSSCG00000002788 | EXOC3L1 | 4.7E-15 | -0.156 |
| LW_ILN | SE | ENSSSCG00000010302 | USP54 | 1.28E-68 | 0.401 |
| LW_ILN | SE | ENSSSCG00000010302 | USP54 | 1.02E-10 | -0.266 |
| LW_ILN | SE | ENSSSCG00000003716 | SS18 | 3.52E-12 | 0.206 |
| LW_ILN | SE | ENSSSCG00000023903 | ATP5I | 2.97E-31 | -0.125 |
| LW_ILN | SE | ENSSSCG00000012396 | MED12 | 0.001972 | -0.122 |
| LW_ILN | SE | ENSSSCG00000011678 | XRN1 | 7.19E-07 | -0.11 |
| LW_ILN | SE | ENSSSCG00000001572 | FGD2 | 9.27E-05 | -0.333 |
| LW_ILN | SE | ENSSSCG00000001572 | FGD2 | 0.010364 | -0.15 |
| LW_ILN | SE | ENSSSCG00000011166 | ZMYND11 | 2.39E-15 | 0.157 |
| LW_ILN | SE | ENSSSCG00000029073 | CACNB4 | 8.03E-27 | -0.228 |
| LW_ILN | SE | ENSSSCG00000025401 | CDK10 | 6.35E-13 | -0.175 |
| LW_ILN | SE | ENSSSCG00000014081 | COL4A3BP | 4.6E-09 | 0.199 |
| LW_ILN | SE | ENSSSCG00000025856 | TMEM106A | 0.029192 | -0.129 |
| LW_ILN | SE | ENSSSCG00000017643 | SEPT4 | 0.006175 | -0.221 |
| LW_ILN | SE | ENSSSCG00000011772 | NA | 1.36E-13 | -0.385 |
| LW_ILN | SE | ENSSSCG00000030801 | GBP6 | 0.000315 | 0.108 |
| LW_ILN | SE | ENSSSCG00000030801 | GBP6 | 6.26E-08 | 0.345 |
| LW_ILN | SE | ENSSSCG00000021669 | NA | 6.04E-17 | -0.15 |
| LW_ILN | SE | ENSSSCG00000027528 | TMEM107 | 0.003859 | -0.132 |
| LW_ILN | SE | ENSSSCG00000021170 | FAR1 | 0.021367 | 0.138 |
| LW_ILN | SE | ENSSSCG00000001410 | BAG6 | 0.025497 | -0.216 |
| LW_ILN | SE | ENSSSCG00000029882 | NA | 0.00586 | 0.418 |
| LW_ILN | SE | ENSSSCG00000027130 | TNFRSF12A | 5.54E-08 | 0.15 |
| LW_ILN | SE | ENSSSCG00000002783 | SLC9A5 | 5.17E-21 | -0.321 |
| LW_ILN | SE | ENSSSCG00000024881 | NA | 1.01E-22 | -0.204 |
| LW_ILN | SE | ENSSSCG00000015882 | BAZ2B | 3.21E-18 | 0.19 |
| LW_ILN | SE | ENSSSCG00000011243 | GOLGA4 | 0.018977 | -0.107 |
| LW_ILN | SE | ENSSSCG00000010741 | EDRF1 | 1.33E-05 | 0.111 |
| LW_ILN | SE | ENSSSCG00000024736 | ACPT | 1.22E-06 | 0.172 |
| LW_ILN | SE | ENSSSCG00000007336 | NNAT | 8.31E-26 | -0.282 |
| LW_ILN | SE | ENSSSCG00000007336 | NNAT | 0.004926 | -0.303 |
| LW_ILN | SE | ENSSSCG00000009148 | LEF1 | 2.25E-27 | -0.136 |
| LW_Spleen | MXE | ENSSSCG00000011090 | NEBL | 8.68E-20 | 0.19 |
| LW_Spleen | MXE | ENSSSCG00000011477 | ACOX2 | 0.0419 | -0.113 |
| LW_Spleen | MXE | ENSSSCG00000011477 | ACOX2 | 0.009917 | 0.164 |
| LW_Spleen | MXE | ENSSSCG00000000519 | GLIPR1 | 0.039062 | 0.13 |
| LW_Spleen | MXE | ENSSSCG00000029198 | CUL5 | 0.001061 | 0.163 |
| LW_Spleen | MXE | ENSSSCG00000004087 | CCDC170 | 1.67E-16 | -0.252 |
| LW_Spleen | MXE | ENSSSCG00000004311 | NA | 0.004984 | 0.154 |
| LW_Spleen | MXE | ENSSSCG00000024736 | ACPT | 2.65E-08 | 0.143 |
| LW_Spleen | MXE | ENSSSCG00000009319 | PAN3 | 8.09E-08 | -0.115 |
| LW_Spleen | MXE | ENSSSCG00000004897 | ZCCHC2 | 2.92E-08 | -0.145 |
| LW_Spleen | MXE | ENSSSCG00000005998 | DEPTOR | 1.38E-10 | -0.12 |
| LW_Spleen | MXE | ENSSSCG00000021494 | NA | 0.000504 | 0.197 |
| LW_Spleen | MXE | ENSSSCG00000004929 | PARP16 | 8.39E-09 | -0.137 |
| LW_Spleen | MXE | ENSSSCG00000025323 | NA | 1.47E-27 | 0.102 |
| LW_Spleen | MXE | ENSSSCG00000021310 | NA | 6.15E-09 | -0.164 |
| LW_Spleen | MXE | ENSSSCG00000006346 | NA | 5.59E-18 | 0.1 |
| LW_Spleen | MXE | ENSSSCG00000022081 | PHLDB2 | 0.000623 | -0.105 |
| LW_Spleen | MXE | ENSSSCG00000030265 | NA | 8.93E-06 | -0.104 |
| LW_Spleen | MXE | ENSSSCG00000015482 | VAMP4 | 0.00918 | 0.14 |
| LW_Spleen | MXE | ENSSSCG00000014998 | AASDHPPT | 2.45E-12 | -0.106 |
| LW_Spleen | MXE | ENSSSCG00000005060 | KTN1 | 0.000928 | 0.146 |
| LW_Spleen | MXE | ENSSSCG00000008639 | NA | 3.73E-18 | 0.11 |
| LW_Spleen | MXE | ENSSSCG00000001700 | SLC29A1 | 3.26E-17 | -0.122 |
| LW_Spleen | MXE | ENSSSCG00000006903 | RPAP2 | 0.038502 | -0.169 |
| LW_Spleen | MXE | ENSSSCG00000013262 | AMBRA1 | 1.47E-06 | -0.106 |
| LW_Spleen | MXE | ENSSSCG00000007855 | NA | 0.017358 | 0.144 |
| LW_Spleen | MXE | ENSSSCG00000012676 | MBNL3 | 0.005885 | -0.306 |
| LW_Spleen | MXE | ENSSSCG00000004634 | NA | 7.15E-19 | -0.11 |
| LW_Spleen | MXE | ENSSSCG00000017509 | RPL19 | 6.92E-09 | 0.112 |
| LW_Spleen | MXE | ENSSSCG00000016005 | SESTD1 | 1.8E-43 | -0.234 |
| LW_Spleen | MXE | ENSSSCG00000015080 | CEP164 | 0.012582 | -0.117 |
| LW_Spleen | MXE | ENSSSCG00000015307 | CDK14 | 0.001361 | -0.158 |
| LW_Spleen | MXE | ENSSSCG00000009998 | GATSL3 | 1.05E-13 | -0.131 |
| LW_Spleen | MXE | ENSSSCG00000014015 | C5orf45 | 4.55E-16 | -0.146 |
| LW_Spleen | MXE | ENSSSCG00000007048 | CRLS1 | 1.54E-39 | 0.216 |
| LW_Spleen | MXE | ENSSSCG00000011700 | CP | 2.3E-11 | 0.182 |
| LW_Spleen | MXE | ENSSSCG00000016073 | NA | 0.037552 | -0.123 |
| LW_Spleen | MXE | ENSSSCG00000006098 | DPY19L4 | 4.31E-08 | 0.145 |
| LW_Spleen | MXE | ENSSSCG00000002712 | LDHD | 0.000275 | 0.165 |
| LW_Spleen | MXE | ENSSSCG00000015775 | DCTD | 0.001411 | 0.163 |
| LW_Spleen | MXE | ENSSSCG00000012914 | RAD9A | 0.000748 | 0.143 |
| LW_Spleen | MXE | ENSSSCG00000010011 | NA | 0.000422 | 0.125 |
| LW_Spleen | MXE | ENSSSCG00000011197 | OXNAD1 | 1.59E-13 | 0.139 |
| LW_Spleen | MXE | ENSSSCG00000016734 | CCM2 | 0.01604 | -0.155 |
| LW_Spleen | MXE | ENSSSCG00000009334 | HSPH1 | 7.87E-90 | -0.158 |
| LW_Spleen | MXE | ENSSSCG00000014581 | TUB | 2.5E-05 | 0.363 |
| LW_Spleen | MXE | ENSSSCG00000023269 | EXOC1 | 9.25E-33 | 0.118 |
| LW_Spleen | MXE | ENSSSCG00000030616 | KIAA1551 | 0.009311 | 0.199 |
| LW_Spleen | MXE | ENSSSCG00000030616 | KIAA1551 | 0.000315 | 0.211 |
| LW_Spleen | MXE | ENSSSCG00000028378 | PHF20 | 1.74E-07 | -0.137 |
| LW_Spleen | MXE | ENSSSCG00000028378 | PHF20 | 0.004587 | 0.132 |
| LW_Spleen | MXE | ENSSSCG00000009061 | NAA15 | 0.01234 | -0.278 |
| LW_Spleen | MXE | ENSSSCG00000024628 | ALKBH8 | 9.41E-05 | -0.106 |
| LW_Spleen | MXE | ENSSSCG00000021803 | NA | 9.84E-10 | 0.139 |
| LW_Spleen | MXE | ENSSSCG00000027438 | NA | 0.035217 | 0.282 |
| LW_Spleen | MXE | ENSSSCG00000001865 | SCAPER | 3.11E-17 | -0.123 |
| LW_Spleen | MXE | ENSSSCG00000012287 | TFE3 | 1.63E-10 | -0.127 |
| LW_Spleen | MXE | ENSSSCG00000015232 | ST3GAL4 | 3.76E-10 | -0.1 |
| LW_Spleen | MXE | ENSSSCG00000002799 | CNOT1 | 9.9E-23 | -0.101 |
| LW_Spleen | MXE | ENSSSCG00000015085 | IL10RA | 2.03E-33 | 0.112 |
| LW_Spleen | MXE | ENSSSCG00000027765 | FAM179B | 0.000631 | 0.104 |
| LW_Spleen | MXE | ENSSSCG00000027765 | FAM179B | 3.16E-22 | -0.118 |
| LW_Spleen | MXE | ENSSSCG00000015789 | SNX25 | 7.89E-25 | -0.177 |
| LW_Spleen | MXE | ENSSSCG00000016043 | OSGEPL1 | 0.000477 | 0.157 |
| LW_Spleen | MXE | ENSSSCG00000013990 | NA | 0.00028 | 0.16 |
| LW_Spleen | MXE | ENSSSCG00000009720 | DDX60 | 0.013605 | -0.116 |
| LW_Spleen | MXE | ENSSSCG00000013380 | NUCB2 | 0.005624 | 0.146 |
| LW_Spleen | MXE | ENSSSCG00000004120 | SHPRH | 3.65E-19 | -0.164 |
| LW_Spleen | MXE | ENSSSCG00000009259 | PAQR3 | 0.023591 | 0.211 |
| LW_Spleen | MXE | ENSSSCG00000000655 | KLRF1 | 0.048064 | 0.167 |
| LW_Spleen | MXE | ENSSSCG00000012880 | NA | 3.25E-21 | -0.121 |
| LW_Spleen | MXE | ENSSSCG00000014126 | MSH3 | 2.21E-36 | 0.157 |
| LW_Spleen | MXE | ENSSSCG00000008103 | NA | 2.7E-27 | 0.132 |
| LW_Spleen | MXE | ENSSSCG00000020763 | NA | 2.21E-05 | -0.141 |
| LW_Spleen | MXE | ENSSSCG00000009650 | DOCK5 | 2.92E-09 | -0.104 |
| LW_Spleen | MXE | ENSSSCG00000006936 | ODF2L | 0.030446 | 0.314 |
| LW_Spleen | MXE | ENSSSCG00000028293 | RCOR3 | 0.002912 | 0.108 |
| LW_Spleen | MXE | ENSSSCG00000017615 | NA | 9.24E-09 | -0.126 |
| LW_Spleen | MXE | ENSSSCG00000022508 | UIMC1 | 1.04E-28 | -0.147 |
| LW_Spleen | MXE | ENSSSCG00000010143 | MTR | 1.15E-10 | 0.115 |
| LW_Spleen | MXE | ENSSSCG00000007735 | RABGEF1 | 1.04E-10 | 0.122 |
| LW_Spleen | MXE | ENSSSCG00000029679 | TRAPPC1 | 2.06E-12 | 0.12 |
| LW_Spleen | MXE | ENSSSCG00000015586 | RPS6KC1 | 0.00018 | -0.108 |
| LW_Spleen | MXE | ENSSSCG00000010056 | GGT1 | 0.000116 | 0.201 |
| LW_Spleen | MXE | ENSSSCG00000014899 | PRCP | 2.62E-14 | -0.1 |
| LW_Spleen | MXE | ENSSSCG00000008099 | ZC3H8 | 6.16E-09 | 0.126 |
| LW_Spleen | MXE | ENSSSCG00000010381 | NA | 3.71E-16 | -0.142 |
| LW_Spleen | MXE | ENSSSCG00000009956 | ADRBK2 | 1.05E-28 | 0.14 |
| LW_Spleen | MXE | ENSSSCG00000012203 | TAB3 | 0.003782 | -0.241 |
| LW_Spleen | MXE | ENSSSCG00000012825 | IKBKG | 9.58E-09 | 0.123 |
| LW_Spleen | MXE | ENSSSCG00000010896 | ASPM | 1.12E-22 | -0.133 |
| LW_Spleen | MXE | ENSSSCG00000030229 | ABHD14A | 0.003028 | -0.14 |
| LW_Spleen | MXE | ENSSSCG00000030229 | ABHD14A | 0.033288 | -0.118 |
| LW_Spleen | MXE | ENSSSCG00000030255 | EIF4G1 | 6.65E-16 | 0.134 |
| LW_Spleen | MXE | ENSSSCG00000030255 | EIF4G1 | 0.002451 | 0.127 |
| LW_Spleen | MXE | ENSSSCG00000013113 | TMEM132A | 0.001127 | -0.156 |
| LW_Spleen | MXE | ENSSSCG00000016041 | NA | 5.49E-05 | 0.107 |
| LW_Spleen | MXE | ENSSSCG00000023565 | IKZF5 | 4.64E-06 | -0.169 |
| LW_Spleen | MXE | ENSSSCG00000014562 | NA | 0.00424 | 0.179 |
| LW_Spleen | MXE | ENSSSCG00000000068 | EP300 | 0.016015 | -0.104 |
| LW_Spleen | MXE | ENSSSCG00000002354 | COQ6 | 0.043457 | -0.228 |
| LW_Spleen | MXE | ENSSSCG00000007331 | RBL1 | 2.53E-25 | -0.137 |
| LW_Spleen | MXE | ENSSSCG00000015112 | HINFP | 7.23E-71 | 0.292 |
| LW_Spleen | MXE | ENSSSCG00000016864 | C5orf51 | 0.014592 | 0.138 |
| LW_Spleen | MXE | ENSSSCG00000009419 | GPALPP1 | 0.001105 | 0.168 |
| LW_Spleen | MXE | ENSSSCG00000011496 | ADAMTS9 | 0.041206 | -0.219 |
| LW_Spleen | MXE | ENSSSCG00000027767 | FAM35A | 0.019644 | 0.108 |
| LW_Spleen | MXE | ENSSSCG00000027767 | FAM35A | 4.54E-06 | 0.119 |
| LW_Spleen | MXE | ENSSSCG00000008515 | MEMO1 | 1.01E-06 | 0.107 |
| LW_Spleen | MXE | ENSSSCG00000013362 | UEVLD | 7.98E-16 | -0.241 |
| LW_Spleen | MXE | ENSSSCG00000014831 | PAAF1 | 5.8E-11 | -0.15 |
| LW_Spleen | MXE | ENSSSCG00000029212 | GDE1 | 1.45E-22 | 0.139 |
| LW_Spleen | MXE | ENSSSCG00000010620 | XPNPEP1 | 6.52E-15 | -0.158 |
| LW_Spleen | MXE | ENSSSCG00000014048 | HK3 | 9.83E-08 | 0.107 |
| LW_Spleen | MXE | ENSSSCG00000003101 | HIF3A | 3.01E-09 | -0.21 |
| LW_Spleen | MXE | ENSSSCG00000013882 | FAM129C | 0.040182 | 0.131 |
| LW_Spleen | MXE | ENSSSCG00000007133 | ACSS1 | 4.56E-68 | 0.186 |
| LW_Spleen | MXE | ENSSSCG00000028755 | TSSC1 | 3.16E-27 | 0.121 |
| LW_Spleen | MXE | ENSSSCG00000028755 | TSSC1 | 0.032284 | 0.117 |
| LW_Spleen | MXE | ENSSSCG00000016970 | NA | 1.37E-10 | -0.218 |
| LW_Spleen | MXE | ENSSSCG00000013297 | CD44 | 0.043912 | 0.309 |
| LW_Spleen | MXE | ENSSSCG00000009664 | PTK2B | 1.35E-35 | -0.136 |
| LW_Spleen | MXE | ENSSSCG00000013322 | IMMP1L | 8.79E-39 | 0.172 |
| LW_Spleen | MXE | ENSSSCG00000028674 | CR2 | 7.62E-28 | -0.222 |
| LW_Spleen | MXE | ENSSSCG00000001970 | HEATR5A | 4.44E-30 | -0.185 |
| LW_Spleen | MXE | ENSSSCG00000015327 | CASD1 | 1.54E-20 | 0.154 |
| LW_Spleen | MXE | ENSSSCG00000014909 | SYTL2 | 0.00143 | -0.329 |
| LW_Spleen | MXE | ENSSSCG00000028504 | RFC2 | 0.003344 | -0.1 |
| LW_Spleen | MXE | ENSSSCG00000001970 | HEATR5A | 5.13E-28 | 0.155 |
| LW_Spleen | MXE | ENSSSCG00000023611 | TNXB | 1.25E-06 | -0.117 |
| LW_Spleen | MXE | ENSSSCG00000009128 | AP1AR | 1.29E-11 | -0.107 |
| LW_Spleen | MXE | ENSSSCG00000001970 | HEATR5A | 0.008248 | 0.109 |
| LW_Spleen | MXE | ENSSSCG00000011079 | PIP4K2A | 2.56E-19 | -0.107 |
| LW_Spleen | MXE | ENSSSCG00000001822 | RCCD1 | 1.87E-13 | -0.187 |
| LW_Spleen | MXE | ENSSSCG00000029539 | RAB23 | 0.012788 | -0.166 |
| LW_Spleen | MXE | ENSSSCG00000025439 | NA | 1.28E-32 | -0.141 |
| LW_Spleen | MXE | ENSSSCG00000014101 | NA | 3.29E-19 | -0.109 |
| LW_Spleen | MXE | ENSSSCG00000003707 | NPC1 | 5.53E-07 | -0.113 |
| LW_Spleen | MXE | ENSSSCG00000017296 | ACE | 0.007045 | -0.121 |
| LW_Spleen | MXE | ENSSSCG00000003905 | RAD54L | 0.004066 | -0.115 |
| LW_Spleen | MXE | ENSSSCG00000008300 | STAMBP | 1.99E-14 | -0.141 |
| LW_Spleen | MXE | ENSSSCG00000014390 | FCHSD1 | 2.24E-05 | -0.211 |
| LW_Spleen | MXE | ENSSSCG00000016882 | PARP8 | 0.002044 | -0.156 |
| LW_Spleen | MXE | ENSSSCG00000011085 | MLLT10 | 0.017574 | -0.103 |
| LW_Spleen | MXE | ENSSSCG00000004657 | CEP152 | 1.5E-28 | -0.21 |
| LW_Spleen | MXE | ENSSSCG00000014902 | ANKRD42 | 5.76E-05 | 0.117 |
| LW_Spleen | MXE | ENSSSCG00000012884 | PPP6R3 | 0.003246 | 0.105 |
| LW_Spleen | MXE | ENSSSCG00000010396 | PARG | 8.39E-07 | -0.106 |
| LW_Spleen | MXE | ENSSSCG00000029989 | MED28 | 4.13E-11 | 0.148 |
| LW_Spleen | MXE | ENSSSCG00000001588 | DNAH8 | 0.008063 | 0.267 |
| LW_Spleen | MXE | ENSSSCG00000014800 | RNF121 | 3.19E-06 | 0.195 |
| LW_Spleen | MXE | ENSSSCG00000004382 | SEC63 | 8.43E-08 | 0.183 |
| LW_Spleen | MXE | ENSSSCG00000004114 | ADGB | 0.00066 | -0.282 |
| LW_Spleen | MXE | ENSSSCG00000004114 | ADGB | 0.030862 | 0.118 |
| LW_Spleen | MXE | ENSSSCG00000029708 | SLC25A38 | 0.004054 | 0.135 |
| LW_Spleen | MXE | ENSSSCG00000004934 | DPP8 | 7.76E-08 | -0.107 |
| LW_Spleen | MXE | ENSSSCG00000004934 | DPP8 | 2.32E-06 | -0.103 |
| LW_Spleen | MXE | ENSSSCG00000029745 | FAM193A | 1.48E-05 | -0.278 |
| LW_Spleen | MXE | ENSSSCG00000009492 | GPR180 | 1.94E-06 | -0.108 |
| LW_Spleen | MXE | ENSSSCG00000024359 | FRA10AC1 | 2.42E-09 | -0.641 |
| LW_Spleen | MXE | ENSSSCG00000011106 | CREM | 0.032955 | -0.211 |
| LW_Spleen | MXE | ENSSSCG00000013896 | MPV17L2 | 1.53E-16 | -0.126 |
| LW_Spleen | MXE | ENSSSCG00000014431 | AFAP1L1 | 7.09E-05 | -0.347 |
| LW_Spleen | MXE | ENSSSCG00000008845 | SRD5A3 | 3.37E-24 | 0.131 |
| LW_Spleen | MXE | ENSSSCG00000006350 | FCGR2B | 0.007582 | 0.142 |
| LW_Spleen | MXE | ENSSSCG00000005364 | TDRD7 | 1.05E-14 | -0.104 |
| LW_Spleen | MXE | ENSSSCG00000011877 | CD86 | 3.49E-24 | 0.101 |
| LW_Spleen | MXE | ENSSSCG00000006543 | ADAR | 0.000171 | 0.143 |
| LW_Spleen | MXE | ENSSSCG00000009069 | C4orf33 | 0.000181 | 0.148 |
| LW_Spleen | MXE | ENSSSCG00000013298 | PDHX | 0.000751 | -0.112 |
| LW_Spleen | MXE | ENSSSCG00000005232 | SMARCA2 | 3.4E-07 | -0.187 |
| LW_Spleen | MXE | ENSSSCG00000011258 | EXOG | 4.81E-06 | -0.121 |
| LW_Spleen | MXE | ENSSSCG00000009652 | KCTD9 | 0.000603 | -0.194 |
| LW_Spleen | MXE | ENSSSCG00000013630 | DNM2 | 2.75E-13 | 0.103 |
| LW_Spleen | MXE | ENSSSCG00000006897 | NA | 9.76E-13 | -0.131 |
| LW_Spleen | MXE | ENSSSCG00000006897 | NA | 0.0085 | -0.139 |
| LW_Spleen | MXE | ENSSSCG00000010294 | NA | 0.011025 | 0.14 |
| LW_Spleen | MXE | ENSSSCG00000027478 | PPCDC | 0.000193 | 0.142 |
| LW_Spleen | A3SS | ENSSSCG00000023014 | CD300LG | 0.018444 | 0.127 |
| LW_Spleen | A3SS | ENSSSCG00000010712 | RPS16 | 2.93E-05 | -0.199 |
| LW_Spleen | A3SS | ENSSSCG00000005503 | TLR4 | 1.78E-05 | -0.18 |
| LW_Spleen | A3SS | ENSSSCG00000005503 | TLR4 | 0.003279 | 0.192 |
| LW_Spleen | A3SS | ENSSSCG00000012576 | CHRDL1 | 0.000693 | -0.105 |
| LW_Spleen | A5SS | ENSSSCG00000005122 | TEK | 0.001754 | -0.106 |
| LW_Spleen | A5SS | ENSSSCG00000000806 | SCAF11 | 0.000196 | -0.124 |
| LW_Spleen | A5SS | ENSSSCG00000015310 | AKAP9 | 1.73E-05 | 0.369 |
| LW_Spleen | A5SS | ENSSSCG00000004535 | TCF4 | 1.31E-21 | 0.118 |
| LW_Spleen | RI | ENSSSCG00000001384 | VARS2 | 8.32E-19 | 0.195 |
| LW_Spleen | RI | ENSSSCG00000030801 | GBP6 | 0.04629 | -0.127 |
| LW_Spleen | RI | ENSSSCG00000004156 | IFNGR1 | 1.89E-16 | 0.334 |
| LW_Spleen | RI | ENSSSCG00000006452 | CD1D | 0.032086 | -0.114 |
| LW_Spleen | RI | ENSSSCG00000012173 | SAT1 | 1.24E-21 | -0.103 |
| LW_Spleen | RI | ENSSSCG00000000648 | CLEC7A | 0.00179 | -0.341 |
| LW_Spleen | RI | ENSSSCG00000007807 | CD19 | 1.38E-08 | 0.114 |
| LW_Spleen | RI | ENSSSCG00000002792 | HSF4 | 1.51E-08 | 0.1 |
| LW_Spleen | SE | ENSSSCG00000014163 | NA | 0.003757 | 0.105 |
| LW_Spleen | SE | ENSSSCG00000007309 | RBM39 | 1.98E-99 | -0.214 |
| LW_Spleen | SE | ENSSSCG00000016643 | TMEM168 | 0.002945 | -0.109 |
| LW_Spleen | SE | ENSSSCG00000013836 | WIZ | 0.004499 | -0.169 |
| LW_Spleen | SE | ENSSSCG00000013836 | WIZ | 0.044361 | 0.322 |
| LW_Spleen | SE | ENSSSCG00000005060 | KTN1 | 0.010177 | 0.168 |
| LW_Spleen | SE | ENSSSCG00000005060 | KTN1 | 0 | -0.174 |
| LW_Spleen | SE | ENSSSCG00000005060 | KTN1 | 0.036873 | -0.12 |
| LW_Spleen | SE | ENSSSCG00000021459 | NA | 5.39E-25 | 0.565 |
| LW_Spleen | SE | ENSSSCG00000013409 | NA | 1.81E-23 | -0.158 |
| LW_Spleen | SE | ENSSSCG00000011485 | PTPRG | 0.003903 | -0.182 |
| LW_Spleen | SE | ENSSSCG00000005288 | TLE4 | 0.001162 | 0.139 |
| LW_Spleen | SE | ENSSSCG00000010313 | NA | 1.18E-26 | -0.189 |
| LW_Spleen | SE | ENSSSCG00000007855 | NA | 0.004381 | -0.23 |
| LW_Spleen | SE | ENSSSCG00000006457 | FCRL3 | 0.00295 | -0.148 |
| LW_Spleen | SE | ENSSSCG00000006066 | RNF19A | 0.022442 | -0.256 |
| LW_Spleen | SE | ENSSSCG00000010987 | UBAP2 | 6.42E-10 | -0.422 |
| LW_Spleen | SE | ENSSSCG00000002755 | NFAT5 | 0.018003 | 0.204 |
| LW_Spleen | SE | ENSSSCG00000012653 | ZDHHC9 | 1.63E-05 | -0.111 |
| LW_Spleen | SE | ENSSSCG00000008383 | AHSA2 | 0.000744 | 0.137 |
| LW_Spleen | SE | ENSSSCG00000027252 | HCFC1R1 | 0.005402 | -0.22 |
| LW_Spleen | SE | ENSSSCG00000027252 | HCFC1R1 | 0.043944 | -0.353 |
| LW_Spleen | SE | ENSSSCG00000003396 | NA | 0.016307 | -0.155 |
| LW_Spleen | SE | ENSSSCG00000024872 | CPT1C | 2.79E-26 | -0.201 |
| LW_Spleen | SE | ENSSSCG00000005308 | RUSC2 | 2.26E-06 | -0.119 |
| LW_Spleen | SE | ENSSSCG00000012676 | MBNL3 | 9.12E-08 | -0.215 |
| LW_Spleen | SE | ENSSSCG00000006000 | TAF2 | 0.001608 | 0.166 |
| LW_Spleen | SE | ENSSSCG00000014015 | C5orf45 | 0.000822 | -0.346 |
| LW_Spleen | SE | ENSSSCG00000009744 | SFSWAP | 0.001576 | -0.105 |
| LW_Spleen | SE | ENSSSCG00000012676 | MBNL3 | 3.72E-35 | -0.615 |
| LW_Spleen | SE | ENSSSCG00000001731 | CENPQ | 0.019789 | 0.321 |
| LW_Spleen | SE | ENSSSCG00000022725 | FLOT1 | 2.04E-14 | -0.103 |
| LW_Spleen | SE | ENSSSCG00000006827 | CYB561D1 | 0.012195 | 0.108 |
| LW_Spleen | SE | ENSSSCG00000015670 | GTDC1 | 6.63E-06 | 0.271 |
| LW_Spleen | SE | ENSSSCG00000000728 | PARP11 | 0.037371 | -0.108 |
| LW_Spleen | SE | ENSSSCG00000013111 | CD6 | 0.000135 | -0.453 |
| LW_Spleen | SE | ENSSSCG00000015175 | VWA5A | 1.64E-06 | 0.133 |
| LW_Spleen | SE | ENSSSCG00000012063 | DSCR3 | 2.23E-12 | 0.147 |
| LW_Spleen | SE | ENSSSCG00000017476 | MSL1 | 3.30E-06 | 0.134 |
| LW_Spleen | SE | ENSSSCG00000003848 | LRP8 | 1.06E-05 | -0.234 |
| LW_Spleen | SE | ENSSSCG00000024780 | ZFAND1 | 1.72E-22 | -0.125 |
| LW_Spleen | SE | ENSSSCG00000029967 | PEX1 | 1.61E-23 | 0.204 |
| LW_Spleen | SE | ENSSSCG00000007780 | ZNF629 | 0.021322 | -0.121 |
| LW_Spleen | SE | ENSSSCG00000008881 | RAPGEF2 | 2.96E-18 | -0.115 |
| LW_Spleen | SE | ENSSSCG00000005043 | DDHD1 | 9.19E-20 | 0.157 |
| LW_Spleen | SE | ENSSSCG00000026473 | NA | 1.59E-18 | -0.179 |
| LW_Spleen | SE | ENSSSCG00000011353 | PFKFB4 | 6.56E-07 | -0.17 |
| LW_Spleen | SE | ENSSSCG00000007656 | PVRIG | 6.64E-19 | 0.128 |
| LW_Spleen | SE | ENSSSCG00000004216 | NA | 1.43E-05 | -0.11 |
| LW_Spleen | SE | ENSSSCG00000014868 | EMSY | 0.016183 | 0.114 |
| LW_Spleen | SE | ENSSSCG00000014868 | EMSY | 2.13E-21 | -0.239 |
| LW_Spleen | SE | ENSSSCG00000009430 | AKAP11 | 2.15E-83 | -0.222 |
| LW_Spleen | SE | ENSSSCG00000014376 | HARS2 | 1.11E-08 | -0.135 |
| LW_Spleen | SE | ENSSSCG00000013857 | EPS15L1 | 7.63E-06 | 0.308 |
| LW_Spleen | SE | ENSSSCG00000013857 | EPS15L1 | 0.041258 | 0.275 |
| LW_Spleen | SE | ENSSSCG00000003784 | LRRIQ3 | 0.019022 | -0.156 |
| LW_Spleen | SE | ENSSSCG00000015883 | MARCH7 | 0 | -0.268 |
| LW_Spleen | SE | ENSSSCG00000027229 | B3GNTL1 | 0.045845 | 0.392 |
| LW_Spleen | SE | ENSSSCG00000007568 | IQCE | 1.71E-18 | 0.25 |
| LW_Spleen | SE | ENSSSCG00000009334 | HSPH1 | 0 | -0.258 |
| LW_Spleen | SE | ENSSSCG00000003202 | NA | 2.79E-65 | -0.163 |
| LW_Spleen | SE | ENSSSCG00000008312 | DYSF | 6.24E-14 | 0.328 |
| LW_Spleen | SE | ENSSSCG00000006682 | POLR3GL | 0.002227 | 0.139 |
| LW_Spleen | SE | ENSSSCG00000010105 | SMPD4 | 0.000658 | 0.217 |
| LW_Spleen | SE | ENSSSCG00000015878 | NA | 8.46E-10 | 0.133 |
| LW_Spleen | SE | ENSSSCG00000004531 | C18orf54 | 1.10E-08 | 0.11 |
| LW_Spleen | SE | ENSSSCG00000011770 | DNAJC19 | 3.44E-13 | -0.11 |
| LW_Spleen | SE | ENSSSCG00000015720 | BIN1 | 6.81E-06 | -0.222 |
| LW_Spleen | SE | ENSSSCG00000001850 | ARPIN | 0.004184 | -0.123 |
| LW_Spleen | SE | ENSSSCG00000006165 | SEC31B | 1.03E-07 | 0.115 |
| LW_Spleen | SE | ENSSSCG00000006165 | SEC31B | 0.014828 | 0.114 |
| LW_Spleen | SE | ENSSSCG00000003177 | BCL2L12 | 0.014187 | 0.149 |
| LW_Spleen | SE | ENSSSCG00000016406 | ESYT2 | 2.10E-08 | 0.16 |
| LW_Spleen | SE | ENSSSCG00000004529 | POLI | 0.031636 | -0.106 |
| LW_Spleen | SE | ENSSSCG00000012262 | KDM6A | 1.10E-08 | 0.124 |
| LW_Spleen | SE | ENSSSCG00000012262 | KDM6A | 5.88E-06 | -0.101 |
| LW_Spleen | SE | ENSSSCG00000004718 | TTBK2 | 0.041582 | 0.133 |
| LW_Spleen | SE | ENSSSCG00000027765 | FAM179B | 0.045708 | -0.104 |
| LW_Spleen | SE | ENSSSCG00000010215 | NA | 0.021104 | -0.137 |
| LW_Spleen | SE | ENSSSCG00000012689 | ZNF75D | 0.001971 | 0.24 |
| LW_Spleen | SE | ENSSSCG00000012689 | ZNF75D | 0.000537 | -0.11 |
| LW_Spleen | SE | ENSSSCG00000012689 | ZNF75D | 0.000325 | 0.38 |
| LW_Spleen | SE | ENSSSCG00000012689 | ZNF75D | 0.026555 | 0.227 |
| LW_Spleen | SE | ENSSSCG00000012689 | ZNF75D | 0.014676 | 0.188 |
| LW_Spleen | SE | ENSSSCG00000016053 | NEMP2 | 0.002717 | 0.108 |
| LW_Spleen | SE | ENSSSCG00000029349 | NA | 0.039642 | -0.18 |
| LW_Spleen | SE | ENSSSCG00000027446 | NA | 5.15E-20 | -0.179 |
| LW_Spleen | SE | ENSSSCG00000022162 | RAB11FIP5 | 0.039278 | -0.278 |
| LW_Spleen | SE | ENSSSCG00000017177 | ST6GALNAC2 | 8.83E-12 | -0.209 |
| LW_Spleen | SE | ENSSSCG00000014394 | RNF14 | 1.02E-43 | -0.134 |
| LW_Spleen | SE | ENSSSCG00000008202 | CNNM4 | 1.40E-07 | -0.193 |
| LW_Spleen | SE | ENSSSCG00000009817 | P2RX7 | 0.000351 | 0.153 |
| LW_Spleen | SE | ENSSSCG00000001420 | EHMT2 | 6.65E-09 | -0.137 |
| LW_Spleen | SE | ENSSSCG00000014240 | CSNK1G3 | 0.002267 | -0.1 |
| LW_Spleen | SE | ENSSSCG00000013380 | NUCB2 | 0.001414 | 0.17 |
| LW_Spleen | SE | ENSSSCG00000029776 | FAM219B | 0.000635 | -0.246 |
| LW_Spleen | SE | ENSSSCG00000015022 | LAYN | 5.40E-18 | -0.138 |
| LW_Spleen | SE | ENSSSCG00000006202 | CSPP1 | 0.001109 | -0.134 |
| LW_Spleen | SE | ENSSSCG00000014267 | NA | 5.13E-17 | -0.178 |
| LW_Spleen | SE | ENSSSCG00000015814 | TACC1 | 0.000411 | 0.245 |
| LW_Spleen | SE | ENSSSCG00000009967 | PITPNB | 2.01E-21 | -0.118 |
| LW_Spleen | SE | ENSSSCG00000004466 | TTK | 0.004403 | 0.102 |
| LW_Spleen | SE | ENSSSCG00000011689 | PLOD2 | 5.62E-08 | -0.315 |
| LW_Spleen | SE | ENSSSCG00000029414 | NA | 2.29E-13 | -0.209 |
| LW_Spleen | SE | ENSSSCG00000011741 | GOLIM4 | 4.41E-66 | -0.194 |
| LW_Spleen | SE | ENSSSCG00000012174 | APOO | 1.87E-13 | -0.333 |
| LW_Spleen | SE | ENSSSCG00000027197 | DYRK1B | 0.002118 | -0.234 |
| LW_Spleen | SE | ENSSSCG00000017894 | WSCD1 | 0.037042 | -0.261 |
| LW_Spleen | SE | ENSSSCG00000016954 | ERBB2IP | 0.015836 | -0.12 |
| LW_Spleen | SE | ENSSSCG00000009759 | SCARB1 | 3.80E-09 | -0.109 |
| LW_Spleen | SE | ENSSSCG00000004729 | GANC | 6.52E-12 | -0.322 |
| LW_Spleen | SE | ENSSSCG00000004729 | GANC | 2.90E-14 | -0.367 |
| LW_Spleen | SE | ENSSSCG00000004729 | GANC | 1.40E-65 | -0.452 |
| LW_Spleen | SE | ENSSSCG00000015294 | CR1 | 0.022928 | 0.119 |
| LW_Spleen | SE | ENSSSCG00000007963 | ZNF263 | 1.41E-05 | 0.161 |
| LW_Spleen | SE | ENSSSCG00000001695 | VEGFA | 1.60E-10 | 0.157 |
| LW_Spleen | SE | ENSSSCG00000026596 | SYNRG | 4.61E-32 | 0.132 |
| LW_Spleen | SE | ENSSSCG00000013788 | NA | 4.61E-08 | -0.143 |
| LW_Spleen | SE | ENSSSCG00000009732 | CHFR | 1.21E-10 | 0.265 |
| LW_Spleen | SE | ENSSSCG00000009142 | SEC24B | 0.013195 | 0.147 |
| LW_Spleen | SE | ENSSSCG00000026218 | FIP1L1 | 4.94E-08 | -0.217 |
| LW_Spleen | SE | ENSSSCG00000003673 | CEP192 | 0.034032 | 0.129 |
| LW_Spleen | SE | ENSSSCG00000011317 | FYCO1 | 0.037152 | -0.108 |
| LW_Spleen | SE | ENSSSCG00000007896 | NA | 0.004991 | -0.532 |
| LW_Spleen | SE | ENSSSCG00000016300 | ATG16L1 | 0.000557 | 0.453 |
| LW_Spleen | SE | ENSSSCG00000001991 | DHRS1 | 1.32E-06 | -0.16 |
| LW_Spleen | SE | ENSSSCG00000011750 | PLD1 | 0.001478 | -0.147 |
| LW_Spleen | SE | ENSSSCG00000026210 | FCRL5 | 0.003007 | 0.125 |
| LW_Spleen | SE | ENSSSCG00000026210 | FCRL5 | 5.31E-10 | -0.118 |
| LW_Spleen | SE | ENSSSCG00000021184 | OSGIN2 | 9.71E-15 | -0.122 |
| LW_Spleen | SE | ENSSSCG00000015586 | RPS6KC1 | 0.002287 | 0.224 |
| LW_Spleen | SE | ENSSSCG00000007981 | NPRL3 | 2.74E-24 | -0.272 |
| LW_Spleen | SE | ENSSSCG00000014303 | JADE2 | 0.001614 | -0.256 |
| LW_Spleen | SE | ENSSSCG00000024274 | NA | 0.026566 | 0.334 |
| LW_Spleen | SE | ENSSSCG00000013174 | CTNND1 | 0.005303 | -0.113 |
| LW_Spleen | SE | ENSSSCG00000013174 | CTNND1 | 2.61E-19 | 0.106 |
| LW_Spleen | SE | ENSSSCG00000012434 | NA | 3.69E-06 | -0.103 |
| LW_Spleen | SE | ENSSSCG00000010134 | TANGO2 | 2.15E-17 | -0.145 |
| LW_Spleen | SE | ENSSSCG00000000681 | P3H3 | 8.31E-08 | -0.144 |
| LW_Spleen | SE | ENSSSCG00000006461 | ARHGEF11 | 0.006949 | 0.144 |
| LW_Spleen | SE | ENSSSCG00000015309 | MTERF1 | 1.55E-08 | 0.256 |
| LW_Spleen | SE | ENSSSCG00000022610 | ZNF317 | 0.02272 | 0.109 |
| LW_Spleen | SE | ENSSSCG00000029979 | NA | 0.016193 | -0.109 |
| LW_Spleen | SE | ENSSSCG00000030791 | DOCK11 | 5.87E-37 | -0.166 |
| LW_Spleen | SE | ENSSSCG00000002828 | LPCAT2 | 3.84E-17 | -0.635 |
| LW_Spleen | SE | ENSSSCG00000009711 | NA | 0.003218 | 0.125 |
| LW_Spleen | SE | ENSSSCG00000014159 | NA | 0.013577 | -0.137 |
| LW_Spleen | SE | ENSSSCG00000023336 | METTL21A | 5.95E-68 | 0.296 |
| LW_Spleen | SE | ENSSSCG00000026071 | NA | 4.95E-16 | 0.107 |
| LW_Spleen | SE | ENSSSCG00000024999 | PPIP5K2 | 6.01E-08 | -0.231 |
| LW_Spleen | SE | ENSSSCG00000005611 | ZNF79 | 4.57E-06 | 0.238 |
| LW_Spleen | SE | ENSSSCG00000015112 | HINFP | 7.49E-17 | -0.182 |
| LW_Spleen | SE | ENSSSCG00000004065 | TIAM2 | 1.03E-07 | 0.539 |
| LW_Spleen | SE | ENSSSCG00000016864 | C5orf51 | 1.15E-24 | -0.139 |
| LW_Spleen | SE | ENSSSCG00000008177 | NA | 0.000428 | 0.127 |
| LW_Spleen | SE | ENSSSCG00000002896 | NA | 0.027646 | 0.112 |
| LW_Spleen | SE | ENSSSCG00000002896 | NA | 0.022044 | -0.317 |
| LW_Spleen | SE | ENSSSCG00000029125 | FAM13B | 1.41E-11 | -0.208 |
| LW_Spleen | SE | ENSSSCG00000023975 | C15orf52 | 0.032867 | -0.152 |
| LW_Spleen | SE | ENSSSCG00000011826 | TMEM44 | 0.009002 | 0.567 |
| LW_Spleen | SE | ENSSSCG00000018049 | NA | 0.045234 | -0.558 |
| LW_Spleen | SE | ENSSSCG00000012076 | MX2 | 0.017233 | -0.1 |
| LW_Spleen | SE | ENSSSCG00000007094 | NA | 0.048771 | -0.215 |
| LW_Spleen | SE | ENSSSCG00000000702 | ZNF384 | 0.038624 | -0.232 |
| LW_Spleen | SE | ENSSSCG00000000002 | GTSE1 | 0.009654 | 0.244 |
| LW_Spleen | SE | ENSSSCG00000009010 | ARFIP1 | 1.50E-39 | 0.17 |
| LW_Spleen | SE | ENSSSCG00000026536 | DCUN1D2 | 0.005177 | 0.699 |
| LW_Spleen | SE | ENSSSCG00000007466 | SLC9A8 | 8.36E-08 | 0.169 |
| LW_Spleen | SE | ENSSSCG00000012328 | HUWE1 | 0.028555 | -0.13 |
| LW_Spleen | SE | ENSSSCG00000004134 | PEX3 | 0.046188 | -0.112 |
| LW_Spleen | SE | ENSSSCG00000016535 | CALD1 | 1.74E-70 | -0.139 |
| LW_Spleen | SE | ENSSSCG00000004249 | CEP85L | 0.037182 | -0.108 |
| LW_Spleen | SE | ENSSSCG00000011496 | ADAMTS9 | 9.81E-14 | -0.11 |
| LW_Spleen | SE | ENSSSCG00000004716 | NA | 5.10E-06 | 0.225 |
| LW_Spleen | SE | ENSSSCG00000024361 | TFB1M | 0.011615 | 0.115 |
| LW_Spleen | SE | ENSSSCG00000011849 | TNK2 | 0.009484 | -0.111 |
| LW_Spleen | SE | ENSSSCG00000027052 | PEX11A | 2.21E-24 | 0.288 |
| LW_Spleen | SE | ENSSSCG00000009815 | CAMKK2 | 1.10E-18 | 0.133 |
| LW_Spleen | SE | ENSSSCG00000012125 | OFD1 | 0.029924 | 0.107 |
| LW_Spleen | SE | ENSSSCG00000010850 | ENAH | 0.001856 | -0.383 |
| LW_Spleen | SE | ENSSSCG00000009356 | RFXAP | 0.012921 | 0.13 |
| LW_Spleen | SE | ENSSSCG00000009714 | NEK1 | 0.031933 | -0.233 |
| LW_Spleen | SE | ENSSSCG00000029841 | ZNF362 | 3.20E-12 | -0.309 |
| LW_Spleen | SE | ENSSSCG00000004538 | WDR7 | 0.003674 | 0.127 |
| LW_Spleen | SE | ENSSSCG00000000753 | WNK1 | 8.08E-25 | 0.123 |
| LW_Spleen | SE | ENSSSCG00000026746 | CEP70 | 0.00192 | 0.262 |
| LW_Spleen | SE | ENSSSCG00000026746 | CEP70 | 3.04E-11 | -0.343 |
| LW_Spleen | SE | ENSSSCG00000008941 | MOB1B | 0.000694 | -0.118 |
| LW_Spleen | SE | ENSSSCG00000004646 | NA | 0.002706 | -0.15 |
| LW_Spleen | SE | ENSSSCG00000010034 | DEPDC5 | 0.000772 | -0.154 |
| LW_Spleen | SE | ENSSSCG00000011182 | ASTE1 | 0.001544 | 0.144 |
| LW_Spleen | SE | ENSSSCG00000007516 | STX16 | 1.16E-12 | 0.209 |
| LW_Spleen | SE | ENSSSCG00000007516 | STX16 | 1.01E-15 | -0.199 |
| LW_Spleen | SE | ENSSSCG00000003021 | MEGF8 | 0.008794 | -0.122 |
| LW_Spleen | SE | ENSSSCG00000028096 | NA | 0.021666 | -0.215 |
| LW_Spleen | SE | ENSSSCG00000030017 | FOXRED1 | 5.09E-05 | -0.225 |
| LW_Spleen | SE | ENSSSCG00000016090 | SPATS2L | 3.80E-08 | 0.145 |
| LW_Spleen | SE | ENSSSCG00000016919 | SETD9 | 8.94E-38 | -0.303 |
| LW_Spleen | SE | ENSSSCG00000023204 | AXIN1 | 6.80E-29 | -0.191 |
| LW_Spleen | SE | ENSSSCG00000013882 | FAM129C | 2.17E-18 | -0.149 |
| LW_Spleen | SE | ENSSSCG00000001881 | MAN2C1 | 0.000157 | 0.202 |
| LW_Spleen | SE | ENSSSCG00000001881 | MAN2C1 | 0.001355 | -0.1 |
| LW_Spleen | SE | ENSSSCG00000000705 | CD27 | 0.03911 | -0.108 |
| LW_Spleen | SE | ENSSSCG00000005715 | NA | 1.31E-25 | 0.154 |
| LW_Spleen | SE | ENSSSCG00000027331 | COL6A3 | 2.38E-49 | 0.13 |
| LW_Spleen | SE | ENSSSCG00000027331 | COL6A3 | 1.27E-06 | 0.197 |
| LW_Spleen | SE | ENSSSCG00000015310 | AKAP9 | 0.001694 | -0.209 |
| LW_Spleen | SE | ENSSSCG00000020887 | NA | 2.29E-10 | 0.333 |
| LW_Spleen | SE | ENSSSCG00000012235 | RPGR | 7.04E-72 | -0.372 |
| LW_Spleen | SE | ENSSSCG00000013297 | CD44 | 1.02E-06 | -0.297 |
| LW_Spleen | SE | ENSSSCG00000021814 | ZMYM2 | 8.13E-05 | -0.109 |
| LW_Spleen | SE | ENSSSCG00000001970 | HEATR5A | 1.68E-26 | -0.109 |
| LW_Spleen | SE | ENSSSCG00000003360 | WRAP73 | 0 | -0.351 |
| LW_Spleen | SE | ENSSSCG00000009978 | AP1B1 | 0.001425 | -0.19 |
| LW_Spleen | SE | ENSSSCG00000013238 | SLC39A13 | 0.000535 | -0.129 |
| LW_Spleen | SE | ENSSSCG00000016174 | FN1 | 1.65E-05 | 0.158 |
| LW_Spleen | SE | ENSSSCG00000016174 | FN1 | 0 | -0.292 |
| LW_Spleen | SE | ENSSSCG00000027417 | LDLRAD4 | 4.15E-06 | -0.115 |
| LW_Spleen | SE | ENSSSCG00000005950 | PHF20L1 | 1.76E-08 | 0.106 |
| LW_Spleen | SE | ENSSSCG00000014060 | KIAA1191 | 4.37E-10 | -0.324 |
| LW_Spleen | SE | ENSSSCG00000013322 | IMMP1L | 1.48E-10 | -0.109 |
| LW_Spleen | SE | ENSSSCG00000013322 | IMMP1L | 2.26E-05 | -0.254 |
| LW_Spleen | SE | ENSSSCG00000013909 | CRLF1 | 0.000193 | -0.163 |
| LW_Spleen | SE | ENSSSCG00000014909 | SYTL2 | 8.66E-05 | 0.338 |
| LW_Spleen | SE | ENSSSCG00000017360 | UBTF | 0.048836 | 0.122 |
| LW_Spleen | SE | ENSSSCG00000017360 | UBTF | 0 | -0.175 |
| LW_Spleen | SE | ENSSSCG00000008293 | DGUOK | 0.026348 | 0.144 |
| LW_Spleen | SE | ENSSSCG00000010055 | GGT5 | 0.026136 | -0.12 |
| LW_Spleen | SE | ENSSSCG00000009151 | CYP2U1 | 1.31E-20 | -0.136 |
| LW_Spleen | SE | ENSSSCG00000009583 | NA | 0.004017 | 0.12 |
| LW_Spleen | SE | ENSSSCG00000002533 | WDR20 | 6.41E-12 | 0.101 |
| LW_Spleen | SE | ENSSSCG00000000968 | SBF1 | 3.34E-05 | -0.131 |
| LW_Spleen | SE | ENSSSCG00000016262 | SP140 | 0.046376 | -0.168 |
| LW_Spleen | SE | ENSSSCG00000002629 | FBXO9 | 2.09E-12 | -0.14 |
| LW_Spleen | SE | ENSSSCG00000000953 | NA | 2.53E-24 | -0.237 |
| LW_Spleen | SE | ENSSSCG00000011393 | UBA7 | 0.034612 | -0.257 |
| LW_Spleen | SE | ENSSSCG00000012178 | NA | 8.55E-58 | 0.139 |
| LW_Spleen | SE | ENSSSCG00000022611 | CAPRIN2 | 4.46E-15 | -0.162 |
| LW_Spleen | SE | ENSSSCG00000010531 | R3HCC1L | 0.00319 | 0.117 |
| LW_Spleen | SE | ENSSSCG00000016378 | PASK | 1.63E-08 | 0.104 |
| LW_Spleen | SE | ENSSSCG00000021075 | TDRD1 | 0.000788 | 0.141 |
| LW_Spleen | SE | ENSSSCG00000004478 | MYO6 | 0.016702 | -0.141 |
| LW_Spleen | SE | ENSSSCG00000000640 | NA | 2.32E-33 | -0.226 |
| LW_Spleen | SE | ENSSSCG00000005367 | TSTD2 | 1.39E-07 | -0.152 |
| LW_Spleen | SE | ENSSSCG00000024315 | NA | 6.01E-09 | -0.167 |
| LW_Spleen | SE | ENSSSCG00000021714 | PRR3 | 0.029915 | 0.172 |
| LW_Spleen | SE | ENSSSCG00000013314 | QSER1 | 0.041488 | -0.108 |
| LW_Spleen | SE | ENSSSCG00000011594 | NR2C2 | 3.72E-21 | 0.346 |
| LW_Spleen | SE | ENSSSCG00000012884 | PPP6R3 | 1.29E-31 | 0.258 |
| LW_Spleen | SE | ENSSSCG00000004701 | CATSPER2 | 0.033566 | 0.102 |
| LW_Spleen | SE | ENSSSCG00000004701 | CATSPER2 | 0.012888 | -0.166 |
| LW_Spleen | SE | ENSSSCG00000024518 | NA | 0.004485 | -0.243 |
| LW_Spleen | SE | ENSSSCG00000015948 | DYNC1I2 | 1.87E-24 | -0.134 |
| LW_Spleen | SE | ENSSSCG00000008392 | BCL11A | 0.000347 | 0.133 |
| LW_Spleen | SE | ENSSSCG00000028148 | DMD | 1.18E-28 | 0.149 |
| LW_Spleen | SE | ENSSSCG00000028148 | DMD | 1.06E-24 | 0.318 |
| LW_Spleen | SE | ENSSSCG00000016147 | PIKFYVE | 0.000127 | 0.123 |
| LW_Spleen | SE | ENSSSCG00000012713 | ATP11C | 2.38E-49 | 0.247 |
| LW_Spleen | SE | ENSSSCG00000001588 | DNAH8 | 0.039811 | -0.317 |
| LW_Spleen | SE | ENSSSCG00000008404 | MTIF2 | 3.69E-36 | -0.192 |
| LW_Spleen | SE | ENSSSCG00000030246 | NA | 0.000363 | 0.111 |
| LW_Spleen | SE | ENSSSCG00000015853 | NA | 5.80E-77 | -0.238 |
| LW_Spleen | SE | ENSSSCG00000010593 | CNNM2 | 0.000518 | -0.251 |
| LW_Spleen | SE | ENSSSCG00000004832 | UBE3A | 2.07E-07 | -0.142 |
| LW_Spleen | SE | ENSSSCG00000001639 | TRERF1 | 0.002641 | -0.394 |
| LW_Spleen | SE | ENSSSCG00000004077 | FBXO5 | 0.041371 | 0.14 |
| LW_Spleen | SE | ENSSSCG00000004077 | FBXO5 | 5.52E-07 | 0.179 |
| LW_Spleen | SE | ENSSSCG00000014156 | ARRDC3 | 9.97E-09 | 0.169 |
| LW_Spleen | SE | ENSSSCG00000000652 | CLEC12A | 0.000782 | 0.128 |
| LW_Spleen | SE | ENSSSCG00000012249 | CXorf38 | 5.31E-05 | -0.102 |
| LW_Spleen | SE | ENSSSCG00000014204 | DCP2 | 0.017752 | 0.206 |
| LW_Spleen | SE | ENSSSCG00000004592 | NA | 5.26E-09 | -0.111 |
| LW_Spleen | SE | ENSSSCG00000005518 | NA | 0.008966 | 0.112 |
| LW_Spleen | SE | ENSSSCG00000015099 | BCL9L | 1.71E-21 | -0.313 |
| LW_Spleen | SE | ENSSSCG00000004997 | PRPF39 | 0.004807 | -0.146 |
| LW_Spleen | SE | ENSSSCG00000012727 | FMR1 | 1.07E-07 | 0.125 |
| LW_Spleen | SE | ENSSSCG00000005602 | GAPVD1 | 0.00016 | -0.133 |
| LW_Spleen | SE | ENSSSCG00000014329 | CDC25C | 7.40E-10 | 0.143 |
| LW_Spleen | SE | ENSSSCG00000017204 | ITGB4 | 0.015855 | -0.444 |
| LW_Spleen | SE | ENSSSCG00000012241 | BCOR | 7.68E-46 | -0.166 |
| LW_Spleen | SE | ENSSSCG00000015129 | ARHGEF12 | 0.012755 | 0.386 |
| LW_Spleen | SE | ENSSSCG00000006912 | HFM1 | 1.68E-18 | 0.221 |
| LW_Spleen | SE | ENSSSCG00000006912 | HFM1 | 3.87E-21 | -0.322 |
| LW_Spleen | SE | ENSSSCG00000008845 | SRD5A3 | 1.03E-05 | 0.193 |
| LW_Spleen | SE | ENSSSCG00000008845 | SRD5A3 | 0.017078 | -0.121 |
| LW_Spleen | SE | ENSSSCG00000004969 | KIF23 | 1.50E-60 | -0.229 |
| LW_Spleen | SE | ENSSSCG00000011553 | NA | 0.001603 | 0.196 |
| LW_Spleen | SE | ENSSSCG00000020720 | MRVI1 | 0.018804 | -0.203 |
| LW_Spleen | SE | ENSSSCG00000023304 | SRRM2 | 0.000408 | -0.159 |
| LW_Spleen | SE | ENSSSCG00000012489 | TRMT2B | 0.006868 | 0.164 |
| LW_Spleen | SE | ENSSSCG00000000971 | NA | 6.51E-06 | -0.119 |
| LW_Spleen | SE | ENSSSCG00000000971 | NA | 1.58E-09 | -0.299 |
| LW_Spleen | SE | ENSSSCG00000010130 | GNB1L | 2.00E-14 | -0.334 |
| LW_Spleen | SE | ENSSSCG00000000541 | FAR2 | 0.017608 | 0.136 |
| LW_Spleen | SE | ENSSSCG00000006203 | COPS5 | 0.029754 | -0.136 |
| LW_Spleen | SE | ENSSSCG00000006203 | COPS5 | 3.84E-17 | -0.329 |
| LW_Spleen | SE | ENSSSCG00000007753 | C16orf58 | 1.69E-08 | 0.223 |
| LW_Spleen | SE | ENSSSCG00000004123 | NA | 1.24E-43 | -0.213 |
| LW_Spleen | SE | ENSSSCG00000003716 | SS18 | 2.04E-55 | 0.205 |
| LW_Spleen | SE | ENSSSCG00000004351 | USP45 | 1.70E-09 | -0.122 |
| LW_Spleen | SE | ENSSSCG00000012396 | MED12 | 0.020245 | -0.122 |
| LW_Spleen | SE | ENSSSCG00000013978 | ZNF672 | 0.034045 | -0.195 |
| LW_Spleen | SE | ENSSSCG00000025731 | ALKBH3 | 0.004491 | -0.294 |
| LW_Spleen | SE | ENSSSCG00000001572 | FGD2 | 0.000652 | -0.161 |
| LW_Spleen | SE | ENSSSCG00000010017 | SMTN | 0 | 0.176 |
| LW_Spleen | SE | ENSSSCG00000011090 | NEBL | 7.11E-15 | -0.111 |
| LW_Spleen | SE | ENSSSCG00000015244 | APLP2 | 2.05E-76 | 0.106 |
| LW_Spleen | SE | ENSSSCG00000030801 | GBP6 | 0 | 0.378 |
| LW_Spleen | SE | ENSSSCG00000001534 | ANKS1A | 0.011722 | 0.154 |
| LW_Spleen | SE | ENSSSCG00000021791 | SENP7 | 1.20E-42 | 0.205 |
| LW_Spleen | SE | ENSSSCG00000021669 | NA | 8.38E-27 | -0.175 |
| LW_Spleen | SE | ENSSSCG00000026517 | NA | 2.03E-12 | -0.421 |
| LW_Spleen | SE | ENSSSCG00000021170 | FAR1 | 0.000988 | 0.156 |
| LW_Spleen | SE | ENSSSCG00000000519 | GLIPR1 | 0.000188 | 0.166 |
| LW_Spleen | SE | ENSSSCG00000029882 | NA | 0.028118 | 0.452 |
| LW_Spleen | SE | ENSSSCG00000021702 | NA | 1.43E-10 | 0.248 |
| LW_Spleen | SE | ENSSSCG00000010351 | CCSER2 | 0.003872 | -0.134 |
| LW_Spleen | SE | ENSSSCG00000015275 | NA | 0.009266 | -0.228 |
| LW_Spleen | SE | ENSSSCG00000015882 | BAZ2B | 3.37E-12 | 0.135 |
| LW_Spleen | SE | ENSSSCG00000024881 | NA | 2.04E-06 | -0.219 |
| LW_Spleen | SE | ENSSSCG00000017400 | NAGLU | 0.029585 | -0.1 |
| LW_Spleen | SE | ENSSSCG00000012508 | ARMCX6 | 1.84E-24 | 0.291 |
| LW_Spleen | SE | ENSSSCG00000025792 | NA | 0.045769 | 0.141 |
| LW_Spleen | SE | ENSSSCG00000024736 | ACPT | 0.000219 | -0.261 |
| LW_Spleen | SE | ENSSSCG00000024736 | ACPT | 2.95E-25 | -0.178 |
| LW_Spleen | SE | ENSSSCG00000011743 | MECOM | 0.046235 | -0.15 |
| LW_Spleen | SE | ENSSSCG00000002266 | CHD2 | 0.007762 | -0.117 |
| LW_Spleen | SE | ENSSSCG00000012660 | SLC25A14 | 0.000816 | 0.333 |
| LW_Spleen | SE | ENSSSCG00000012660 | SLC25A14 | 0.013006 | 0.279 |
| LW_Spleen | SE | ENSSSCG00000009560 | NA | 5.91E-05 | -0.185 |
| LW_Spleen | SE | ENSSSCG00000027312 | PTCH1 | 0.038172 | 0.246 |
| LW_Spleen | SE | ENSSSCG00000020775 | NA | 1.33E-21 | -0.257 |
| LW_Spleen | SE | ENSSSCG00000021663 | NA | 0.020182 | -0.296 |
| LW_Spleen | SE | ENSSSCG00000012944 | PELI3 | 0.000973 | 0.344 |
| LW_Spleen | SE | ENSSSCG00000005914 | SHARPIN | 1.41E-21 | -0.186 |
| TC_ILN | A3SS | ENSSSCG00000006357 | FCER1G | 1.50E-20 | -0.169 |
| TC_ILN | A3SS | ENSSSCG00000023014 | CD300LG | 6.14E-06 | 0.348 |
| TC_ILN | A3SS | ENSSSCG00000023014 | CD300LG | 8.47E-07 | 0.281 |
| TC_ILN | A3SS | ENSSSCG00000005503 | TLR4 | 1.19E-07 | -0.115 |
| TC_ILN | A3SS | ENSSSCG00000012583 | ACSL4 | 5.62E-06 | -0.12 |
| TC_ILN | A3SS | ENSSSCG00000017022 | HMMR | 1.05E-18 | -0.166 |
| TC_ILN | A5SS | ENSSSCG00000015558 | SMG7 | 8.18E-51 | -0.112 |
| TC_ILN | A5SS | ENSSSCG00000004535 | TCF4 | 0.02707 | 0.109 |
| TC_ILN | MXE | ENSSSCG00000002382 | TMED10 | 7.61E-44 | 0.155 |
| TC_ILN | MXE | ENSSSCG00000011731 | SMC4 | 0.000479 | 0.134 |
| TC_ILN | MXE | ENSSSCG00000006363 | UFC1 | 0.001322 | 0.104 |
| TC_ILN | MXE | ENSSSCG00000017566 | ACSF2 | 1.29E-44 | -0.127 |
| TC_ILN | MXE | ENSSSCG00000005038 | PSMC6 | 1.96E-30 | 0.264 |
| TC_ILN | MXE | ENSSSCG00000006038 | ZFPM2 | 4.65E-91 | 0.197 |
| TC_ILN | MXE | ENSSSCG00000008559 | TMEM214 | 1.11E-07 | 0.226 |
| TC_ILN | MXE | ENSSSCG00000000511 | RAB21 | 0.047356 | 0.103 |
| TC_ILN | MXE | ENSSSCG00000011136 | FAM208B | 0.002165 | -0.109 |
| TC_ILN | MXE | ENSSSCG00000017072 | GALNT10 | 4.1E-08 | -0.126 |
| TC_ILN | MXE | ENSSSCG00000001009 | RIPK1 | 4.64E-39 | 0.173 |
| TC_ILN | MXE | ENSSSCG00000005621 | CDK9 | 0.000577 | 0.138 |
| TC_ILN | MXE | ENSSSCG00000011772 | NA | 1.92E-10 | -0.107 |
| TC_ILN | MXE | ENSSSCG00000016857 | DAB2 | 0.015249 | -0.141 |
| TC_ILN | MXE | ENSSSCG00000028855 | NA | 0.009244 | 0.119 |
| TC_ILN | MXE | ENSSSCG00000030413 | PIK3CA | 0.00108 | -0.127 |
| TC_ILN | MXE | ENSSSCG00000017100 | MTRR | 1.47E-38 | 0.176 |
| TC_ILN | MXE | ENSSSCG00000010380 | MAPK8 | 2.13E-14 | 0.121 |
| TC_ILN | MXE | ENSSSCG00000029882 | NA | 6.87E-05 | -0.382 |
| TC_ILN | MXE | ENSSSCG00000014969 | NA | 4.49E-05 | 0.163 |
| TC_ILN | MXE | ENSSSCG00000014558 | SIRT3 | 0.017157 | 0.259 |
| TC_ILN | MXE | ENSSSCG00000016088 | NA | 1.44E-13 | 0.109 |
| TC_ILN | MXE | ENSSSCG00000002015 | AP1G2 | 0.014033 | 0.137 |
| TC_ILN | MXE | ENSSSCG00000007951 | CREBBP | 1.8E-13 | -0.131 |
| TC_ILN | MXE | ENSSSCG00000012435 | MAGT1 | 0.000141 | 0.13 |
| TC_ILN | MXE | ENSSSCG00000015039 | BCO2 | 1.99E-07 | -0.196 |
| TC_ILN | MXE | ENSSSCG00000011243 | GOLGA4 | 8.16E-09 | 0.221 |
| TC_ILN | MXE | ENSSSCG00000010897 | ZBTB41 | 3.55E-50 | 0.212 |
| TC_ILN | MXE | ENSSSCG00000024736 | ACPT | 1.85E-09 | -0.159 |
| TC_ILN | MXE | ENSSSCG00000024736 | ACPT | 0.027789 | -0.12 |
| TC_ILN | MXE | ENSSSCG00000001725 | ADGRF5 | 0.029944 | -0.125 |
| TC_ILN | MXE | ENSSSCG00000017754 | NA | 0.005963 | 0.135 |
| TC_ILN | MXE | ENSSSCG00000002790 | TMEM208 | 0.000154 | 0.173 |
| TC_ILN | MXE | ENSSSCG00000002266 | CHD2 | 0.00014 | 0.121 |
| TC_ILN | MXE | ENSSSCG00000007187 | NSFL1C | 9.88E-10 | 0.205 |
| TC_ILN | MXE | ENSSSCG00000011336 | ELP6 | 0.044969 | -0.108 |
| TC_ILN | MXE | ENSSSCG00000017412 | RAB5C | 6.68E-15 | 0.14 |
| TC_ILN | MXE | ENSSSCG00000003153 | FTL | 2.8E-179 | 0.178 |
| TC_ILN | MXE | ENSSSCG00000017104 | NSUN2 | 9.37E-12 | 0.281 |
| TC_ILN | MXE | ENSSSCG00000009372 | MRPS31 | 8.84E-21 | 0.155 |
| TC_ILN | MXE | ENSSSCG00000004943 | MAP2K1 | 5.06E-10 | 0.109 |
| TC_ILN | MXE | ENSSSCG00000001878 | PTPN9 | 5.26E-33 | 0.157 |
| TC_ILN | MXE | ENSSSCG00000001974 | G2E3 | 0.000403 | -0.112 |
| TC_ILN | MXE | ENSSSCG00000008556 | KHK | 9.59E-08 | -0.159 |
| TC_ILN | MXE | ENSSSCG00000000964 | LMF2 | 2.24E-05 | 0.21 |
| TC_ILN | MXE | ENSSSCG00000012118 | TLR8 | 2.07E-12 | 0.158 |
| TC_ILN | MXE | ENSSSCG00000022081 | PHLDB2 | 2.38E-05 | -0.11 |
| TC_ILN | MXE | ENSSSCG00000010566 | NA | 1.1E-136 | 0.353 |
| TC_ILN | MXE | ENSSSCG00000030265 | NA | 0.032811 | 0.127 |
| TC_ILN | MXE | ENSSSCG00000022955 | POLR2B | 0.003766 | 0.132 |
| TC_ILN | MXE | ENSSSCG00000015570 | IVNS1ABP | 0.014916 | 0.123 |
| TC_ILN | MXE | ENSSSCG00000016128 | EEF1B2 | 4.34E-38 | -0.18 |
| TC_ILN | MXE | ENSSSCG00000010226 | JMJD1C | 0.039637 | 0.137 |
| TC_ILN | MXE | ENSSSCG00000029837 | NA | 0.025155 | -0.237 |
| TC_ILN | MXE | ENSSSCG00000014307 | H2AFY | 0.003068 | 0.121 |
| TC_ILN | MXE | ENSSSCG00000008877 | ETFDH | 2.76E-19 | -0.167 |
| TC_ILN | MXE | ENSSSCG00000023212 | PPP1R14A | 1.53E-12 | -0.297 |
| TC_ILN | MXE | ENSSSCG00000005000 | FANCM | 1.12E-10 | -0.17 |
| TC_ILN | MXE | ENSSSCG00000024023 | HNMT | 0.026964 | 0.121 |
| TC_ILN | MXE | ENSSSCG00000016194 | USP37 | 0.00335 | -0.177 |
| TC_ILN | MXE | ENSSSCG00000009400 | RCBTB2 | 3.3E-24 | 0.154 |
| TC_ILN | MXE | ENSSSCG00000002774 | FAM65A | 1.77E-17 | -0.147 |
| TC_ILN | MXE | ENSSSCG00000017509 | RPL19 | 9.45E-16 | -0.208 |
| TC_ILN | MXE | ENSSSCG00000017509 | RPL19 | 7.37E-05 | -0.125 |
| TC_ILN | MXE | ENSSSCG00000000553 | TM7SF3 | 0.022428 | 0.127 |
| TC_ILN | MXE | ENSSSCG00000014190 | NA | 3.92E-07 | 0.125 |
| TC_ILN | MXE | ENSSSCG00000005308 | RUSC2 | 0.027395 | -0.158 |
| TC_ILN | MXE | ENSSSCG00000011889 | GSK3B | 1.53E-07 | -0.113 |
| TC_ILN | MXE | ENSSSCG00000010136 | TRMT2A | 8.36E-05 | -0.118 |
| TC_ILN | MXE | ENSSSCG00000010528 | ZFYVE27 | 0.003637 | 0.145 |
| TC_ILN | MXE | ENSSSCG00000015080 | CEP164 | 2.79E-09 | 0.15 |
| TC_ILN | MXE | ENSSSCG00000024623 | USP25 | 0.000504 | 0.177 |
| TC_ILN | MXE | ENSSSCG00000007789 | NA | 0.016394 | 0.104 |
| TC_ILN | MXE | ENSSSCG00000016828 | DNAJC21 | 5.11E-06 | 0.161 |
| TC_ILN | MXE | ENSSSCG00000000167 | RIC8B | 0.010582 | -0.157 |
| TC_ILN | MXE | ENSSSCG00000002299 | RAD51B | 0.01485 | -0.135 |
| TC_ILN | MXE | ENSSSCG00000001512 | CUTA | 0.005536 | -0.217 |
| TC_ILN | MXE | ENSSSCG00000006098 | DPY19L4 | 0.000754 | 0.152 |
| TC_ILN | MXE | ENSSSCG00000022674 | DAZAP2 | 7.76E-07 | 0.152 |
| TC_ILN | MXE | ENSSSCG00000011197 | OXNAD1 | 0.000169 | -0.241 |
| TC_ILN | MXE | ENSSSCG00000029577 | NOP16 | 5.77E-35 | 0.262 |
| TC_ILN | MXE | ENSSSCG00000027046 | USP9Y | 2.22E-40 | 0.139 |
| TC_ILN | MXE | ENSSSCG00000008540 | PPP1CB | 2.69E-21 | 0.139 |
| TC_ILN | MXE | ENSSSCG00000024316 | COX5A | 0.013136 | 0.125 |
| TC_ILN | MXE | ENSSSCG00000024316 | COX5A | 0.041581 | -0.111 |
| TC_ILN | MXE | ENSSSCG00000029806 | NA | 0.00555 | 0.127 |
| TC_ILN | MXE | ENSSSCG00000015947 | DCAF17 | 0.021734 | -0.235 |
| TC_ILN | MXE | ENSSSCG00000025425 | FUK | 3.58E-09 | 0.145 |
| TC_ILN | MXE | ENSSSCG00000011833 | DLG1 | 1.62E-30 | -0.165 |
| TC_ILN | MXE | ENSSSCG00000023269 | EXOC1 | 0.030272 | -0.117 |
| TC_ILN | MXE | ENSSSCG00000013857 | EPS15L1 | 0.000513 | -0.148 |
| TC_ILN | MXE | ENSSSCG00000030616 | KIAA1551 | 0.000689 | 0.151 |
| TC_ILN | MXE | ENSSSCG00000006538 | FLAD1 | 3.58E-12 | -0.108 |
| TC_ILN | MXE | ENSSSCG00000009112 | NA | 1.57E-26 | 0.138 |
| TC_ILN | MXE | ENSSSCG00000011471 | FLNB | 0.018927 | -0.107 |
| TC_ILN | MXE | ENSSSCG00000012779 | ABCD1 | 0.02673 | 0.189 |
| TC_ILN | MXE | ENSSSCG00000009145 | OSTC | 0.000888 | 0.151 |
| TC_ILN | MXE | ENSSSCG00000022742 | PRDX6 | 1.4E-81 | 0.186 |
| TC_ILN | MXE | ENSSSCG00000010250 | VPS26A | 8.9E-19 | 0.11 |
| TC_ILN | MXE | ENSSSCG00000010706 | SAMD4B | 0.006269 | 0.166 |
| TC_ILN | MXE | ENSSSCG00000006165 | SEC31B | 9.06E-05 | 0.231 |
| TC_ILN | MXE | ENSSSCG00000004936 | VWA9 | 0.000635 | -0.111 |
| TC_ILN | MXE | ENSSSCG00000004498 | HAUS1 | 1.1E-05 | 0.108 |
| TC_ILN | MXE | ENSSSCG00000015866 | FMNL2 | 5.69E-16 | -0.158 |
| TC_ILN | MXE | ENSSSCG00000027765 | FAM179B | 0.000129 | 0.114 |
| TC_ILN | MXE | ENSSSCG00000027765 | FAM179B | 0.042503 | 0.139 |
| TC_ILN | MXE | ENSSSCG00000030222 | ANAPC7 | 1.62E-16 | -0.106 |
| TC_ILN | MXE | ENSSSCG00000030222 | ANAPC7 | 4.71E-11 | 0.246 |
| TC_ILN | MXE | ENSSSCG00000009548 | ANKRD10 | 1.6E-05 | 0.173 |
| TC_ILN | MXE | ENSSSCG00000010070 | SMARCB1 | 2.04E-65 | -0.141 |
| TC_ILN | MXE | ENSSSCG00000009551 | NA | 1.54E-22 | 0.159 |
| TC_ILN | MXE | ENSSSCG00000015721 | ERCC3 | 7.3E-76 | 0.187 |
| TC_ILN | MXE | ENSSSCG00000002440 | CCDC88C | 0.007836 | 0.131 |
| TC_ILN | MXE | ENSSSCG00000003830 | MYSM1 | 6.71E-06 | -0.137 |
| TC_ILN | MXE | ENSSSCG00000003872 | EPS15 | 9E-169 | 0.19 |
| TC_ILN | MXE | ENSSSCG00000003872 | EPS15 | 3.93E-30 | 0.101 |
| TC_ILN | MXE | ENSSSCG00000024172 | PAFAH2 | 5.36E-18 | -0.119 |
| TC_ILN | MXE | ENSSSCG00000014915 | NA | 6.43E-14 | 0.12 |
| TC_ILN | MXE | ENSSSCG00000024201 | FAM208A | 9.22E-27 | 0.279 |
| TC_ILN | MXE | ENSSSCG00000009890 | MAPKAPK5 | 4.18E-25 | -0.243 |
| TC_ILN | MXE | ENSSSCG00000029249 | NA | 8.57E-25 | 0.307 |
| TC_ILN | MXE | ENSSSCG00000005689 | FNBP1 | 6.74E-06 | 0.136 |
| TC_ILN | MXE | ENSSSCG00000008406 | CCDC88A | 0.034509 | -0.118 |
| TC_ILN | MXE | ENSSSCG00000007342 | RPRD1B | 1.35E-37 | 0.147 |
| TC_ILN | MXE | ENSSSCG00000017102 | PAPD7 | 3.52E-06 | 0.114 |
| TC_ILN | MXE | ENSSSCG00000009720 | DDX60 | 0.003785 | -0.238 |
| TC_ILN | MXE | ENSSSCG00000013380 | NUCB2 | 1.11E-08 | 0.217 |
| TC_ILN | MXE | ENSSSCG00000010652 | FAM160B1 | 9.16E-17 | 0.116 |
| TC_ILN | MXE | ENSSSCG00000008247 | SUCLG1 | 9.29E-28 | 0.133 |
| TC_ILN | MXE | ENSSSCG00000017755 | NOS2 | 1.51E-08 | -0.2 |
| TC_ILN | MXE | ENSSSCG00000000723 | C12orf4 | 0.001815 | 0.264 |
| TC_ILN | MXE | ENSSSCG00000002740 | AP1G1 | 7.54E-51 | 0.164 |
| TC_ILN | MXE | ENSSSCG00000015843 | UBXN8 | 2.88E-08 | 0.17 |
| TC_ILN | MXE | ENSSSCG00000015843 | UBXN8 | 1.13E-07 | 0.141 |
| TC_ILN | MXE | ENSSSCG00000014126 | MSH3 | 7.21E-36 | -0.195 |
| TC_ILN | MXE | ENSSSCG00000002429 | FOXN3 | 7.06E-26 | -0.156 |
| TC_ILN | MXE | ENSSSCG00000011804 | RFC4 | 5.73E-05 | 0.24 |
| TC_ILN | MXE | ENSSSCG00000003042 | NA | 0.000181 | 0.13 |
| TC_ILN | MXE | ENSSSCG00000009886 | TRAFD1 | 0.000104 | -0.163 |
| TC_ILN | MXE | ENSSSCG00000012486 | CSTF2 | 0.026307 | 0.122 |
| TC_ILN | MXE | ENSSSCG00000016750 | YKT6 | 0.006195 | 0.182 |
| TC_ILN | MXE | ENSSSCG00000004941 | DIS3L | 0.009085 | 0.149 |
| TC_ILN | MXE | ENSSSCG00000028293 | RCOR3 | 0.001838 | -0.189 |
| TC_ILN | MXE | ENSSSCG00000009732 | CHFR | 2.58E-08 | -0.102 |
| TC_ILN | MXE | ENSSSCG00000013572 | PNPLA6 | 5.47E-18 | 0.127 |
| TC_ILN | MXE | ENSSSCG00000011367 | ARIH2 | 2.58E-73 | 0.181 |
| TC_ILN | MXE | ENSSSCG00000011367 | ARIH2 | 0.013148 | 0.162 |
| TC_ILN | MXE | ENSSSCG00000014082 | POLK | 0.010454 | -0.103 |
| TC_ILN | MXE | ENSSSCG00000014249 | MARCH3 | 0.000207 | -0.138 |
| TC_ILN | MXE | ENSSSCG00000006274 | NA | 4.91E-24 | -0.136 |
| TC_ILN | MXE | ENSSSCG00000006274 | NA | 3.03E-10 | 0.106 |
| TC_ILN | MXE | ENSSSCG00000029760 | CIPC | 0.000148 | 0.224 |
| TC_ILN | MXE | ENSSSCG00000009658 | BNIP3L | 1.02E-07 | 0.176 |
| TC_ILN | MXE | ENSSSCG00000016954 | ERBB2IP | 6.04E-24 | 0.113 |
| TC_ILN | MXE | ENSSSCG00000004582 | NA | 5.51E-12 | 0.132 |
| TC_ILN | MXE | ENSSSCG00000001991 | DHRS1 | 0.02943 | -0.168 |
| TC_ILN | MXE | ENSSSCG00000023171 | NA | 0.000631 | -0.142 |
| TC_ILN | MXE | ENSSSCG00000008222 | NA | 0.000254 | 0.143 |
| TC_ILN | MXE | ENSSSCG00000008222 | NA | 0.004566 | -0.11 |
| TC_ILN | MXE | ENSSSCG00000000656 | CLEC2B | 0.010477 | 0.112 |
| TC_ILN | MXE | ENSSSCG00000008614 | SMC6 | 7.62E-51 | -0.104 |
| TC_ILN | MXE | ENSSSCG00000008614 | SMC6 | 1.29E-75 | 0.206 |
| TC_ILN | MXE | ENSSSCG00000027049 | SNAPIN | 1.19E-19 | -0.263 |
| TC_ILN | MXE | ENSSSCG00000000477 | HELB | 3.21E-25 | 0.208 |
| TC_ILN | MXE | ENSSSCG00000016743 | OGDH | 5.74E-18 | 0.108 |
| TC_ILN | MXE | ENSSSCG00000010319 | SAMD8 | 0.010986 | 0.116 |
| TC_ILN | MXE | ENSSSCG00000022830 | KANSL1L | 0.028622 | 0.122 |
| TC_ILN | MXE | ENSSSCG00000000555 | NA | 3.99E-74 | -0.148 |
| TC_ILN | MXE | ENSSSCG00000014343 | NA | 1.24E-33 | 0.149 |
| TC_ILN | MXE | ENSSSCG00000009834 | ATXN2 | 0.002004 | 0.171 |
| TC_ILN | MXE | ENSSSCG00000022508 | UIMC1 | 9.71E-23 | -0.13 |
| TC_ILN | MXE | ENSSSCG00000012825 | IKBKG | 7.08E-50 | -0.205 |
| TC_ILN | MXE | ENSSSCG00000022508 | UIMC1 | 4.76E-12 | -0.154 |
| TC_ILN | MXE | ENSSSCG00000003085 | CLPTM1 | 1.28E-06 | 0.153 |
| TC_ILN | MXE | ENSSSCG00000017378 | DHX8 | 0.020336 | -0.132 |
| TC_ILN | MXE | ENSSSCG00000010180 | TTC13 | 8.86E-07 | -0.18 |
| TC_ILN | MXE | ENSSSCG00000007066 | SLX4IP | 0.000465 | -0.13 |
| TC_ILN | MXE | ENSSSCG00000014887 | NA | 4.07E-15 | 0.303 |
| TC_ILN | MXE | ENSSSCG00000025140 | ZNF292 | 8.69E-05 | 0.182 |
| TC_ILN | MXE | ENSSSCG00000011317 | FYCO1 | 5.14E-29 | -0.135 |
| TC_ILN | MXE | ENSSSCG00000027139 | FYTTD1 | 2.31E-08 | 0.108 |
| TC_ILN | MXE | ENSSSCG00000030255 | EIF4G1 | 4.83E-06 | 0.123 |
| TC_ILN | MXE | ENSSSCG00000015700 | TMEM163 | 0.035648 | 0.101 |
| TC_ILN | MXE | ENSSSCG00000021590 | RPL23 | 8.9E-19 | -0.109 |
| TC_ILN | MXE | ENSSSCG00000021590 | RPL23 | 2.1E-34 | 0.14 |
| TC_ILN | MXE | ENSSSCG00000009818 | NA | 0.001633 | 0.333 |
| TC_ILN | MXE | ENSSSCG00000030791 | DOCK11 | 5.23E-05 | -0.103 |
| TC_ILN | MXE | ENSSSCG00000001549 | NA | 0.005413 | 0.121 |
| TC_ILN | MXE | ENSSSCG00000007440 | CD40 | 0.013927 | 0.101 |
| TC_ILN | MXE | ENSSSCG00000007440 | CD40 | 8.6E-10 | -0.15 |
| TC_ILN | MXE | ENSSSCG00000003405 | PEX14 | 0.005445 | -0.178 |
| TC_ILN | MXE | ENSSSCG00000007979 | LUC7L | 5.22E-12 | 0.287 |
| TC_ILN | MXE | ENSSSCG00000024673 | NME1 | 1.27E-50 | 0.125 |
| TC_ILN | MXE | ENSSSCG00000009524 | TMTC4 | 0.001732 | 0.165 |
| TC_ILN | MXE | ENSSSCG00000016772 | NA | 3.06E-28 | -0.116 |
| TC_ILN | MXE | ENSSSCG00000015090 | MPZL3 | 2.17E-18 | 0.159 |
| TC_ILN | MXE | ENSSSCG00000004457 | DOPEY1 | 2.21E-12 | -0.394 |
| TC_ILN | MXE | ENSSSCG00000003670 | RLF | 0.008031 | 0.111 |
| TC_ILN | MXE | ENSSSCG00000003670 | RLF | 0.029866 | 0.105 |
| TC_ILN | MXE | ENSSSCG00000027984 | ZWINT | 1.02E-16 | 0.148 |
| TC_ILN | MXE | ENSSSCG00000009244 | SEC31A | 0.031643 | -0.116 |
| TC_ILN | MXE | ENSSSCG00000001703 | NFKBIE | 8.36E-29 | 0.122 |
| TC_ILN | MXE | ENSSSCG00000013766 | IL27RA | 0.033665 | 0.111 |
| TC_ILN | MXE | ENSSSCG00000016864 | C5orf51 | 7.93E-05 | 0.213 |
| TC_ILN | MXE | ENSSSCG00000011880 | EAF2 | 5.64E-51 | 0.194 |
| TC_ILN | MXE | ENSSSCG00000013889 | RPL18A | 3.94E-30 | -0.157 |
| TC_ILN | MXE | ENSSSCG00000014074 | GFM2 | 5.58E-05 | -0.109 |
| TC_ILN | MXE | ENSSSCG00000013373 | SERGEF | 2.68E-08 | 0.268 |
| TC_ILN | MXE | ENSSSCG00000009612 | DOK2 | 0.000269 | 0.2 |
| TC_ILN | MXE | ENSSSCG00000010822 | BPNT1 | 0.006221 | 0.138 |
| TC_ILN | MXE | ENSSSCG00000010822 | BPNT1 | 1.16E-05 | 0.1 |
| TC_ILN | MXE | ENSSSCG00000007589 | NA | 0.000444 | -0.279 |
| TC_ILN | MXE | ENSSSCG00000010551 | BLOC1S2 | 1.62E-07 | 0.132 |
| TC_ILN | MXE | ENSSSCG00000003642 | INPP5B | 3.5E-10 | 0.121 |
| TC_ILN | MXE | ENSSSCG00000000288 | HNRNPA1 | 0.003401 | 0.115 |
| TC_ILN | MXE | ENSSSCG00000001456 | SLA-DQA | 8.9E-222 | -0.1 |
| TC_ILN | MXE | ENSSSCG00000001456 | SLA-DQA | 5.93E-62 | 0.262 |
| TC_ILN | MXE | ENSSSCG00000017301 | TLK2 | 7.53E-07 | -0.145 |
| TC_ILN | MXE | ENSSSCG00000007513 | RAB22A | 0.001406 | 0.175 |
| TC_ILN | MXE | ENSSSCG00000000902 | NR2C1 | 7.71E-27 | -0.158 |
| TC_ILN | MXE | ENSSSCG00000005056 | DLGAP5 | 1.84E-15 | 0.248 |
| TC_ILN | MXE | ENSSSCG00000027767 | FAM35A | 2.4E-30 | -0.186 |
| TC_ILN | MXE | ENSSSCG00000015104 | TRAPPC4 | 2.59E-12 | 0.126 |
| TC_ILN | MXE | ENSSSCG00000000902 | NR2C1 | 1.66E-63 | 0.223 |
| TC_ILN | MXE | ENSSSCG00000015104 | TRAPPC4 | 5.6E-50 | 0.161 |
| TC_ILN | MXE | ENSSSCG00000014014 | TBC1D9B | 7.2E-06 | 0.113 |
| TC_ILN | MXE | ENSSSCG00000007331 | RBL1 | 9.9E-05 | -0.1 |
| TC_ILN | MXE | ENSSSCG00000004745 | RPAP1 | 7.68E-05 | -0.106 |
| TC_ILN | MXE | ENSSSCG00000015793 | UFSP2 | 3.83E-12 | 0.253 |
| TC_ILN | MXE | ENSSSCG00000004893 | VPS4B | 1.23E-26 | -0.163 |
| TC_ILN | MXE | ENSSSCG00000002426 | ZC3H14 | 1.54E-61 | 0.228 |
| TC_ILN | MXE | ENSSSCG00000002426 | ZC3H14 | 1.29E-31 | -0.199 |
| TC_ILN | MXE | ENSSSCG00000003017 | TGFB1 | 6.16E-38 | -0.123 |
| TC_ILN | MXE | ENSSSCG00000017502 | PGAP3 | 4.37E-14 | -0.13 |
| TC_ILN | MXE | ENSSSCG00000013115 | CD5 | 1.26E-12 | -0.147 |
| TC_ILN | MXE | ENSSSCG00000005725 | TTF1 | 3.85E-10 | 0.129 |
| TC_ILN | MXE | ENSSSCG00000014831 | PAAF1 | 1.9E-17 | -0.272 |
| TC_ILN | MXE | ENSSSCG00000004002 | RPS5 | 7.7E-191 | 0.168 |
| TC_ILN | MXE | ENSSSCG00000011010 | C9orf72 | 2.85E-11 | 0.203 |
| TC_ILN | MXE | ENSSSCG00000023306 | ELMO2 | 2.8E-11 | 0.149 |
| TC_ILN | MXE | ENSSSCG00000027723 | NA | 1.79E-15 | 0.16 |
| TC_ILN | MXE | ENSSSCG00000002495 | SYNE3 | 0.011201 | 0.144 |
| TC_ILN | MXE | ENSSSCG00000010620 | XPNPEP1 | 0.001943 | -0.22 |
| TC_ILN | MXE | ENSSSCG00000000295 | NA | 0.010727 | -0.106 |
| TC_ILN | MXE | ENSSSCG00000015861 | WDR33 | 5.31E-26 | -0.135 |
| TC_ILN | MXE | ENSSSCG00000028563 | HNRNPR | 0.000207 | 0.136 |
| TC_ILN | MXE | ENSSSCG00000000683 | CDCA3 | 6.04E-23 | 0.233 |
| TC_ILN | MXE | ENSSSCG00000017668 | VMP1 | 2.6E-15 | 0.13 |
| TC_ILN | MXE | ENSSSCG00000011895 | PLA1A | 0.010065 | -0.168 |
| TC_ILN | MXE | ENSSSCG00000010391 | ERCC6 | 3.25E-08 | -0.122 |
| TC_ILN | MXE | ENSSSCG00000024230 | MYL9 | 4.22E-12 | 0.185 |
| TC_ILN | MXE | ENSSSCG00000018027 | TVP23B | 0.004088 | 0.158 |
| TC_ILN | MXE | ENSSSCG00000006382 | SLAMF6 | 0.03581 | 0.168 |
| TC_ILN | MXE | ENSSSCG00000006382 | SLAMF6 | 0.001811 | 0.161 |
| TC_ILN | MXE | ENSSSCG00000030269 | B4GALNT2 | 1.61E-18 | 0.143 |
| TC_ILN | MXE | ENSSSCG00000014147 | CCNH | 0.000868 | 0.107 |
| TC_ILN | MXE | ENSSSCG00000014292 | HSPA4 | 8.26E-11 | 0.161 |
| TC_ILN | MXE | ENSSSCG00000010628 | SHOC2 | 6.22E-11 | 0.103 |
| TC_ILN | MXE | ENSSSCG00000013426 | MUM1 | 1.81E-29 | 0.196 |
| TC_ILN | MXE | ENSSSCG00000009021 | NA | 4.73E-10 | -0.146 |
| TC_ILN | MXE | ENSSSCG00000015310 | AKAP9 | 4.78E-13 | 0.104 |
| TC_ILN | MXE | ENSSSCG00000015310 | AKAP9 | 1.3E-74 | 0.301 |
| TC_ILN | MXE | ENSSSCG00000008316 | NAGK | 5.01E-33 | 0.174 |
| TC_ILN | MXE | ENSSSCG00000015286 | DSTYK | 1.05E-06 | -0.146 |
| TC_ILN | MXE | ENSSSCG00000028755 | TSSC1 | 2.65E-15 | -0.12 |
| TC_ILN | MXE | ENSSSCG00000028755 | TSSC1 | 3.85E-05 | 0.114 |
| TC_ILN | MXE | ENSSSCG00000028755 | TSSC1 | 3.72E-15 | 0.129 |
| TC_ILN | MXE | ENSSSCG00000028755 | TSSC1 | 8.02E-48 | -0.163 |
| TC_ILN | MXE | ENSSSCG00000003360 | WRAP73 | 0.002158 | 0.136 |
| TC_ILN | MXE | ENSSSCG00000002026 | EFS | 2.81E-10 | 0.238 |
| TC_ILN | MXE | ENSSSCG00000014829 | MRPL48 | 2.58E-22 | -0.166 |
| TC_ILN | MXE | ENSSSCG00000007895 | ZC3H7A | 3.29E-05 | -0.18 |
| TC_ILN | MXE | ENSSSCG00000008010 | CHTF18 | 0.011633 | 0.123 |
| TC_ILN | MXE | ENSSSCG00000008722 | NA | 2.99E-34 | -0.129 |
| TC_ILN | MXE | ENSSSCG00000001398 | SLA-6 | 0.033693 | 0.114 |
| TC_ILN | MXE | ENSSSCG00000028674 | CR2 | 1.48E-27 | 0.206 |
| TC_ILN | MXE | ENSSSCG00000026925 | EMC4 | 6.66E-85 | 0.227 |
| TC_ILN | MXE | ENSSSCG00000002660 | MTHFSD | 1.64E-08 | 0.2 |
| TC_ILN | MXE | ENSSSCG00000007684 | PRKRIP1 | 0.003612 | 0.185 |
| TC_ILN | MXE | ENSSSCG00000004411 | ZBTB24 | 0.00088 | 0.136 |
| TC_ILN | MXE | ENSSSCG00000000657 | CLEC2D | 9.5E-154 | -0.115 |
| TC_ILN | MXE | ENSSSCG00000010497 | ENTPD1 | 3.19E-61 | 0.17 |
| TC_ILN | MXE | ENSSSCG00000013906 | KXD1 | 4.77E-16 | 0.167 |
| TC_ILN | MXE | ENSSSCG00000000033 | TSPO | 6.17E-31 | 0.112 |
| TC_ILN | MXE | ENSSSCG00000022317 | SLC38A10 | 2.47E-43 | -0.14 |
| TC_ILN | MXE | ENSSSCG00000008125 | NA | 2.28E-40 | 0.168 |
| TC_ILN | MXE | ENSSSCG00000014148 | TMEM161B | 8.32E-09 | 0.133 |
| TC_ILN | MXE | ENSSSCG00000013585 | EVI5L | 0.011234 | 0.116 |
| TC_ILN | MXE | ENSSSCG00000011620 | NA | 1.55E-29 | 0.183 |
| TC_ILN | MXE | ENSSSCG00000027658 | NA | 0.018058 | 0.124 |
| TC_ILN | MXE | ENSSSCG00000006337 | HSD17B7 | 1.64E-06 | 0.115 |
| TC_ILN | MXE | ENSSSCG00000021819 | NA | 2.07E-07 | -0.135 |
| TC_ILN | MXE | ENSSSCG00000011393 | UBA7 | 0.000223 | -0.103 |
| TC_ILN | MXE | ENSSSCG00000011393 | UBA7 | 3.85E-07 | 0.132 |
| TC_ILN | MXE | ENSSSCG00000011393 | UBA7 | 0.033038 | 0.1 |
| TC_ILN | MXE | ENSSSCG00000003691 | MYL12B | 6.7E-22 | 0.198 |
| TC_ILN | MXE | ENSSSCG00000011857 | LMLN | 9.52E-45 | 0.657 |
| TC_ILN | MXE | ENSSSCG00000000663 | KLRG1 | 7.86E-27 | -0.145 |
| TC_ILN | MXE | ENSSSCG00000001884 | COMMD4 | 0.041382 | 0.125 |
| TC_ILN | MXE | ENSSSCG00000011901 | TMEM39A | 0.037803 | 0.133 |
| TC_ILN | MXE | ENSSSCG00000009071 | JADE1 | 0.000134 | 0.117 |
| TC_ILN | MXE | ENSSSCG00000009833 | SH2B3 | 3.3E-52 | 0.26 |
| TC_ILN | MXE | ENSSSCG00000011085 | MLLT10 | 4.1E-176 | 0.272 |
| TC_ILN | MXE | ENSSSCG00000016095 | NA | 1.56E-64 | 0.167 |
| TC_ILN | MXE | ENSSSCG00000012149 | RBBP7 | 1.04E-14 | 0.15 |
| TC_ILN | MXE | ENSSSCG00000014902 | ANKRD42 | 0.013373 | -0.117 |
| TC_ILN | MXE | ENSSSCG00000009963 | TPST2 | 0.011924 | 0.126 |
| TC_ILN | MXE | ENSSSCG00000012149 | RBBP7 | 3.78E-05 | 0.105 |
| TC_ILN | MXE | ENSSSCG00000002368 | LTBP2 | 0.005046 | -0.115 |
| TC_ILN | MXE | ENSSSCG00000015480 | PRRC2C | 8.64E-05 | -0.101 |
| TC_ILN | MXE | ENSSSCG00000030174 | AP3S2 | 6.84E-09 | 0.106 |
| TC_ILN | MXE | ENSSSCG00000024825 | RPL6 | 1.4E-103 | -0.133 |
| TC_ILN | MXE | ENSSSCG00000028517 | SC5D | 9.2E-08 | 0.135 |
| TC_ILN | MXE | ENSSSCG00000004077 | FBXO5 | 2.61E-16 | -0.155 |
| TC_ILN | MXE | ENSSSCG00000012132 | ASB9 | 0.048049 | -0.13 |
| TC_ILN | MXE | ENSSSCG00000014800 | RNF121 | 5.39E-10 | 0.202 |
| TC_ILN | MXE | ENSSSCG00000023247 | OPTN | 2.33E-15 | 0.107 |
| TC_ILN | MXE | ENSSSCG00000010024 | RNF185 | 0.002872 | 0.29 |
| TC_ILN | MXE | ENSSSCG00000029697 | NA | 2.97E-11 | -0.13 |
| TC_ILN | MXE | ENSSSCG00000012742 | MTM1 | 4.36E-25 | 0.154 |
| TC_ILN | MXE | ENSSSCG00000008404 | MTIF2 | 1.76E-09 | 0.101 |
| TC_ILN | MXE | ENSSSCG00000004934 | DPP8 | 0.00037 | -0.125 |
| TC_ILN | MXE | ENSSSCG00000010401 | NA | 2.24E-09 | -0.12 |
| TC_ILN | MXE | ENSSSCG00000015115 | CBL | 0.006429 | -0.117 |
| TC_ILN | MXE | ENSSSCG00000012727 | FMR1 | 2.97E-15 | -0.252 |
| TC_ILN | MXE | ENSSSCG00000021746 | MRPL35 | 1.14E-25 | 0.173 |
| TC_ILN | MXE | ENSSSCG00000000900 | VEZT | 1.96E-05 | 0.116 |
| TC_ILN | MXE | ENSSSCG00000017764 | KIAA0100 | 2.72E-39 | -0.142 |
| TC_ILN | MXE | ENSSSCG00000006529 | SLC50A1 | 0.040325 | -0.112 |
| TC_ILN | MXE | ENSSSCG00000006529 | SLC50A1 | 0.008688 | 0.116 |
| TC_ILN | MXE | ENSSSCG00000006529 | SLC50A1 | 1.72E-05 | 0.141 |
| TC_ILN | MXE | ENSSSCG00000013896 | MPV17L2 | 8.08E-15 | 0.186 |
| TC_ILN | MXE | ENSSSCG00000012850 | DEAF1 | 2.4E-74 | -0.26 |
| TC_ILN | MXE | ENSSSCG00000006388 | NA | 1.45E-31 | 0.118 |
| TC_ILN | MXE | ENSSSCG00000020720 | MRVI1 | 0.022802 | -0.309 |
| TC_ILN | MXE | ENSSSCG00000000838 | SLC41A2 | 6.57E-12 | -0.141 |
| TC_ILN | MXE | ENSSSCG00000010258 | AIFM2 | 0.031232 | 0.13 |
| TC_ILN | MXE | ENSSSCG00000012489 | TRMT2B | 9.11E-08 | -0.309 |
| TC_ILN | MXE | ENSSSCG00000012489 | TRMT2B | 5.93E-14 | -0.127 |
| TC_ILN | MXE | ENSSSCG00000006283 | SCYL3 | 1.48E-24 | -0.207 |
| TC_ILN | MXE | ENSSSCG00000006203 | COPS5 | 0.003465 | 0.119 |
| TC_ILN | MXE | ENSSSCG00000002628 | ICK | 0.012469 | -0.157 |
| TC_ILN | MXE | ENSSSCG00000003629 | AGO4 | 1.01E-17 | 0.115 |
| TC_ILN | MXE | ENSSSCG00000029518 | RIOK3 | 4.91E-65 | 0.159 |
| TC_ILN | MXE | ENSSSCG00000025417 | BBS2 | 6.46E-06 | -0.254 |
| TC_ILN | MXE | ENSSSCG00000027017 | NR1D2 | 0.00157 | 0.133 |
| TC_ILN | MXE | ENSSSCG00000009240 | PLAC8 | 2.4E-171 | 0.195 |
| TC_ILN | MXE | ENSSSCG00000016784 | ANKH | 0.028681 | 0.151 |
| TC_ILN | MXE | ENSSSCG00000011382 | USP4 | 1.24E-35 | -0.146 |
| TC_ILN | MXE | ENSSSCG00000006897 | NA | 7.23E-09 | -0.166 |
| TC_ILN | MXE | ENSSSCG00000015961 | CDCA7 | 0.000283 | -0.117 |
| TC_ILN | MXE | ENSSSCG00000003862 | NA | 0.012144 | 0.133 |
| TC_ILN | MXE | ENSSSCG00000017264 | AMZ2 | 1.03E-07 | -0.107 |
| TC_ILN | MXE | ENSSSCG00000001572 | FGD2 | 0.002474 | 0.118 |
| TC_ILN | RI | ENSSSCG00000001384 | VARS2 | 6.73E-17 | -0.106 |
| TC_ILN | RI | ENSSSCG00000013242 | ACP2 | 0.008 | -0.146 |
| TC_ILN | RI | ENSSSCG00000022107 | CHTOP | 0 | -0.27 |
| TC_ILN | RI | ENSSSCG00000006452 | CD1D | 0 | -0.361 |
| TC_ILN | RI | ENSSSCG00000025736 | RECQL4 | 2.83E-07 | -0.318 |
| TC_ILN | RI | ENSSSCG00000003350 | MIB2 | 6.83E-34 | -0.204 |
| TC_ILN | RI | ENSSSCG00000012173 | SAT1 | 0 | -0.291 |
| TC_ILN | RI | ENSSSCG00000001424 | SKIV2L | 3.56E-13 | -0.123 |
| TC_ILN | RI | ENSSSCG00000011393 | UBA7 | 3.39E-08 | -0.13 |
| TC_ILN | RI | ENSSSCG00000008217 | CD8A | 0.014356 | -0.144 |
| TC_ILN | SE | ENSSSCG00000000964 | LMF2 | 1.70E-06 | 0.127 |
| TC_ILN | SE | ENSSSCG00000012118 | TLR8 | 2.16E-18 | -0.175 |
| TC_ILN | SE | ENSSSCG00000010566 | NA | 1.28E-20 | 0.145 |
| TC_ILN | SE | ENSSSCG00000013836 | WIZ | 1.61E-07 | -0.114 |
| TC_ILN | SE | ENSSSCG00000021459 | NA | 2.45E-23 | 0.397 |
| TC_ILN | SE | ENSSSCG00000005060 | KTN1 | 0.032268 | -0.1 |
| TC_ILN | SE | ENSSSCG00000011385 | NA | 0.016893 | 0.205 |
| TC_ILN | SE | ENSSSCG00000006457 | FCRL3 | 1.52E-08 | -0.246 |
| TC_ILN | SE | ENSSSCG00000006457 | FCRL3 | 2.85E-29 | 0.116 |
| TC_ILN | SE | ENSSSCG00000010794 | PALB2 | 0.000541 | -0.179 |
| TC_ILN | SE | ENSSSCG00000027738 | LRRFIP2 | 1.73E-50 | -0.138 |
| TC_ILN | SE | ENSSSCG00000014307 | H2AFY | 5.50E-10 | -0.131 |
| TC_ILN | SE | ENSSSCG00000008937 | AMBN | 0.040176 | 0.204 |
| TC_ILN | SE | ENSSSCG00000010987 | UBAP2 | 0.004022 | 0.228 |
| TC_ILN | SE | ENSSSCG00000002755 | NFAT5 | 0.006669 | -0.135 |
| TC_ILN | SE | ENSSSCG00000001033 | SLC35B3 | 2.38E-13 | -0.326 |
| TC_ILN | SE | ENSSSCG00000000061 | PMM1 | 0.011041 | -0.163 |
| TC_ILN | SE | ENSSSCG00000007200 | FAM110A | 0.008402 | 0.1 |
| TC_ILN | SE | ENSSSCG00000006184 | TERF1 | 1.28E-05 | -0.387 |
| TC_ILN | SE | ENSSSCG00000000003 | TTC38 | 3.65E-05 | -0.1 |
| TC_ILN | SE | ENSSSCG00000016194 | USP37 | 0.0363 | -0.117 |
| TC_ILN | SE | ENSSSCG00000027252 | HCFC1R1 | 2.03E-08 | 0.122 |
| TC_ILN | SE | ENSSSCG00000027252 | HCFC1R1 | 2.63E-07 | -0.181 |
| TC_ILN | SE | ENSSSCG00000016005 | SESTD1 | 0.001852 | -0.141 |
| TC_ILN | SE | ENSSSCG00000006000 | TAF2 | 9.21E-07 | 0.116 |
| TC_ILN | SE | ENSSSCG00000022401 | AGTRAP | 3.76E-18 | -0.115 |
| TC_ILN | SE | ENSSSCG00000000167 | RIC8B | 0.000615 | -0.22 |
| TC_ILN | SE | ENSSSCG00000009744 | SFSWAP | 1.84E-35 | -0.147 |
| TC_ILN | SE | ENSSSCG00000009123 | CAMK2D | 0.00625 | 0.211 |
| TC_ILN | SE | ENSSSCG00000026305 | PDCD1LG2 | 4.24E-10 | -0.118 |
| TC_ILN | SE | ENSSSCG00000000215 | SMARCD1 | 2.93E-09 | -0.114 |
| TC_ILN | SE | ENSSSCG00000000728 | PARP11 | 0.000735 | -0.167 |
| TC_ILN | SE | ENSSSCG00000007521 | NELFCD | 0.003906 | -0.103 |
| TC_ILN | SE | ENSSSCG00000014391 | ARAP3 | 0.012597 | -0.251 |
| TC_ILN | SE | ENSSSCG00000011049 | DCLRE1C | 0.002321 | -0.295 |
| TC_ILN | SE | ENSSSCG00000010030 | EIF4ENIF1 | 0.0242 | 0.117 |
| TC_ILN | SE | ENSSSCG00000014349 | TMEM173 | 0.004652 | 0.234 |
| TC_ILN | SE | ENSSSCG00000014349 | TMEM173 | 0.02945 | 0.142 |
| TC_ILN | SE | ENSSSCG00000004332 | BACH2 | 2.00E-27 | -0.145 |
| TC_ILN | SE | ENSSSCG00000005826 | PHPT1 | 5.14E-26 | -0.224 |
| TC_ILN | SE | ENSSSCG00000008881 | RAPGEF2 | 0.000537 | -0.519 |
| TC_ILN | SE | ENSSSCG00000011684 | U2SURP | 0.014279 | -0.132 |
| TC_ILN | SE | ENSSSCG00000004408 | SMPD2 | 7.38E-25 | 0.113 |
| TC_ILN | SE | ENSSSCG00000014868 | EMSY | 7.11E-34 | -0.351 |
| TC_ILN | SE | ENSSSCG00000025028 | XIAP | 0.003903 | -0.101 |
| TC_ILN | SE | ENSSSCG00000011075 | KIAA1217 | 0.005894 | -0.175 |
| TC_ILN | SE | ENSSSCG00000011075 | KIAA1217 | 9.21E-05 | -0.569 |
| TC_ILN | SE | ENSSSCG00000011347 | NME6 | 0.014483 | 0.108 |
| TC_ILN | SE | ENSSSCG00000023269 | EXOC1 | 0.006369 | 0.186 |
| TC_ILN | SE | ENSSSCG00000013857 | EPS15L1 | 0.00128 | -0.164 |
| TC_ILN | SE | ENSSSCG00000009334 | HSPH1 | 0 | -0.139 |
| TC_ILN | SE | ENSSSCG00000023820 | NA | 0.006521 | -0.164 |
| TC_ILN | SE | ENSSSCG00000015883 | MARCH7 | 0 | -0.247 |
| TC_ILN | SE | ENSSSCG00000007568 | IQCE | 3.13E-18 | -0.194 |
| TC_ILN | SE | ENSSSCG00000024628 | ALKBH8 | 0.000254 | 0.13 |
| TC_ILN | SE | ENSSSCG00000003202 | NA | 3.59E-11 | -0.434 |
| TC_ILN | SE | ENSSSCG00000003468 | PLEKHM2 | 0 | -0.25 |
| TC_ILN | SE | ENSSSCG00000006491 | PAQR6 | 0.036655 | -0.1 |
| TC_ILN | SE | ENSSSCG00000004531 | C18orf54 | 6.64E-19 | -0.258 |
| TC_ILN | SE | ENSSSCG00000005623 | ST6GALNAC6 | 6.76E-23 | 0.255 |
| TC_ILN | SE | ENSSSCG00000015720 | BIN1 | 7.56E-08 | -0.122 |
| TC_ILN | SE | ENSSSCG00000005225 | NA | 2.63E-35 | -0.129 |
| TC_ILN | SE | ENSSSCG00000006165 | SEC31B | 0.000217 | 0.254 |
| TC_ILN | SE | ENSSSCG00000016406 | ESYT2 | 0.00019 | -0.166 |
| TC_ILN | SE | ENSSSCG00000010953 | ISCA1 | 1.22E-05 | -0.126 |
| TC_ILN | SE | ENSSSCG00000004529 | POLI | 7.41E-08 | -0.135 |
| TC_ILN | SE | ENSSSCG00000012262 | KDM6A | 1.50E-06 | -0.131 |
| TC_ILN | SE | ENSSSCG00000001471 | BRD2 | 6.58E-05 | -0.116 |
| TC_ILN | SE | ENSSSCG00000027765 | FAM179B | 7.94E-10 | -0.103 |
| TC_ILN | SE | ENSSSCG00000017550 | SLC35B1 | 6.24E-09 | -0.169 |
| TC_ILN | SE | ENSSSCG00000012689 | ZNF75D | 5.18E-07 | 0.147 |
| TC_ILN | SE | ENSSSCG00000012689 | ZNF75D | 6.53E-06 | -0.146 |
| TC_ILN | SE | ENSSSCG00000012689 | ZNF75D | 2.24E-05 | 0.146 |
| TC_ILN | SE | ENSSSCG00000012689 | ZNF75D | 3.10E-14 | 0.123 |
| TC_ILN | SE | ENSSSCG00000016053 | NEMP2 | 0.000489 | 0.159 |
| TC_ILN | SE | ENSSSCG00000027446 | NA | 1.28E-07 | -0.193 |
| TC_ILN | SE | ENSSSCG00000007930 | MGRN1 | 0.003575 | -0.179 |
| TC_ILN | SE | ENSSSCG00000004653 | SECISBP2L | 4.05E-25 | -0.104 |
| TC_ILN | SE | ENSSSCG00000026429 | ZFP14 | 4.09E-08 | -0.282 |
| TC_ILN | SE | ENSSSCG00000001420 | EHMT2 | 0.030573 | -0.135 |
| TC_ILN | SE | ENSSSCG00000002235 | LPCAT4 | 0.022819 | -0.116 |
| TC_ILN | SE | ENSSSCG00000013723 | DHPS | 0.010301 | -0.224 |
| TC_ILN | SE | ENSSSCG00000015022 | LAYN | 4.21E-09 | -0.166 |
| TC_ILN | SE | ENSSSCG00000006202 | CSPP1 | 0.001437 | -0.242 |
| TC_ILN | SE | ENSSSCG00000016395 | RIF1 | 4.77E-89 | -0.253 |
| TC_ILN | SE | ENSSSCG00000011689 | PLOD2 | 0.012263 | -0.142 |
| TC_ILN | SE | ENSSSCG00000011741 | GOLIM4 | 0.000834 | -0.161 |
| TC_ILN | SE | ENSSSCG00000009673 | NUGGC | 4.04E-14 | 0.127 |
| TC_ILN | SE | ENSSSCG00000027197 | DYRK1B | 0.002436 | -0.206 |
| TC_ILN | SE | ENSSSCG00000014184 | PAM | 1.70E-30 | -0.19 |
| TC_ILN | SE | ENSSSCG00000007248 | ASXL1 | 0 | -0.289 |
| TC_ILN | SE | ENSSSCG00000016520 | CREB3L2 | 2.81E-11 | -0.109 |
| TC_ILN | SE | ENSSSCG00000002429 | FOXN3 | 0.002266 | -0.143 |
| TC_ILN | SE | ENSSSCG00000009759 | SCARB1 | 5.51E-26 | 0.153 |
| TC_ILN | SE | ENSSSCG00000023296 | CENPE | 7.40E-29 | -0.131 |
| TC_ILN | SE | ENSSSCG00000003042 | NA | 6.86E-10 | -0.124 |
| TC_ILN | SE | ENSSSCG00000015999 | FKBP7 | 1.71E-17 | -0.163 |
| TC_ILN | SE | ENSSSCG00000008040 | TSC2 | 1.56E-09 | 0.21 |
| TC_ILN | SE | ENSSSCG00000009823 | VPS29 | 0.001164 | -0.19 |
| TC_ILN | SE | ENSSSCG00000004303 | NA | 3.64E-09 | -0.229 |
| TC_ILN | SE | ENSSSCG00000009142 | SEC24B | 4.47E-07 | 0.107 |
| TC_ILN | SE | ENSSSCG00000009230 | WDFY3 | 5.20E-12 | -0.149 |
| TC_ILN | SE | ENSSSCG00000016002 | CCDC141 | 4.46E-14 | -0.186 |
| TC_ILN | SE | ENSSSCG00000026218 | FIP1L1 | 2.18E-18 | -0.13 |
| TC_ILN | SE | ENSSSCG00000011950 | NXPE3 | 0.001722 | 0.175 |
| TC_ILN | SE | ENSSSCG00000023099 | NA | 5.89E-15 | 0.102 |
| TC_ILN | SE | ENSSSCG00000010519 | NA | 0.017596 | -0.127 |
| TC_ILN | SE | ENSSSCG00000007473 | ADNP | 2.43E-33 | -0.239 |
| TC_ILN | SE | ENSSSCG00000009238 | COQ2 | 3.21E-45 | -0.471 |
| TC_ILN | SE | ENSSSCG00000027857 | DMXL1 | 0.007919 | -0.31 |
| TC_ILN | SE | ENSSSCG00000007981 | NPRL3 | 0.004277 | -0.104 |
| TC_ILN | SE | ENSSSCG00000024274 | NA | 0.027903 | -0.135 |
| TC_ILN | SE | ENSSSCG00000014303 | JADE2 | 0.000738 | -0.289 |
| TC_ILN | SE | ENSSSCG00000027432 | NSUN3 | 0.001478 | -0.188 |
| TC_ILN | SE | ENSSSCG00000006461 | ARHGEF11 | 0.044384 | -0.121 |
| TC_ILN | SE | ENSSSCG00000022610 | ZNF317 | 0.004828 | 0.2 |
| TC_ILN | SE | ENSSSCG00000008736 | CPEB2 | 9.50E-19 | 0.2 |
| TC_ILN | SE | ENSSSCG00000015368 | HDAC9 | 0.000124 | -0.116 |
| TC_ILN | SE | ENSSSCG00000001887 | SCAMP5 | 0.006973 | -0.16 |
| TC_ILN | SE | ENSSSCG00000014370 | TMCO6 | 2.49E-33 | 0.229 |
| TC_ILN | SE | ENSSSCG00000014141 | RFESD | 0.003051 | 0.104 |
| TC_ILN | SE | ENSSSCG00000011235 | UBP1 | 0 | -0.213 |
| TC_ILN | SE | ENSSSCG00000001242 | GABBR1 | 1.61E-18 | -0.157 |
| TC_ILN | SE | ENSSSCG00000007924 | NA | 0.000295 | -0.174 |
| TC_ILN | SE | ENSSSCG00000023336 | METTL21A | 3.83E-11 | 0.121 |
| TC_ILN | SE | ENSSSCG00000009783 | OGFOD2 | 3.43E-11 | 0.215 |
| TC_ILN | SE | ENSSSCG00000010886 | NA | 5.30E-05 | 0.129 |
| TC_ILN | SE | ENSSSCG00000004457 | DOPEY1 | 2.31E-06 | 0.161 |
| TC_ILN | SE | ENSSSCG00000008262 | SEMA4F | 0.029737 | -0.195 |
| TC_ILN | SE | ENSSSCG00000009244 | SEC31A | 0.000329 | 0.256 |
| TC_ILN | SE | ENSSSCG00000011776 | YEATS2 | 0.00055 | -0.102 |
| TC_ILN | SE | ENSSSCG00000017743 | CRLF3 | 8.67E-17 | -0.38 |
| TC_ILN | SE | ENSSSCG00000017795 | NSRP1 | 0.032112 | -0.132 |
| TC_ILN | SE | ENSSSCG00000005465 | SUSD1 | 5.73E-07 | -0.471 |
| TC_ILN | SE | ENSSSCG00000025492 | NUDT13 | 4.23E-11 | 0.138 |
| TC_ILN | SE | ENSSSCG00000020858 | NA | 0.000132 | -0.223 |
| TC_ILN | SE | ENSSSCG00000017277 | PECAM1 | 0.007349 | -0.114 |
| TC_ILN | SE | ENSSSCG00000007331 | RBL1 | 0.005984 | -0.1 |
| TC_ILN | SE | ENSSSCG00000012656 | ELF4 | 0.004715 | -0.108 |
| TC_ILN | SE | ENSSSCG00000029125 | FAM13B | 3.46E-08 | -0.166 |
| TC_ILN | SE | ENSSSCG00000002896 | NA | 2.26E-06 | -0.11 |
| TC_ILN | SE | ENSSSCG00000000702 | ZNF384 | 0.000468 | -0.175 |
| TC_ILN | SE | ENSSSCG00000010276 | UNC5B | 8.33E-06 | 0.284 |
| TC_ILN | SE | ENSSSCG00000003859 | ZCCHC11 | 0.00272 | -0.481 |
| TC_ILN | SE | ENSSSCG00000003859 | ZCCHC11 | 0.000732 | -0.163 |
| TC_ILN | SE | ENSSSCG00000007466 | SLC9A8 | 3.97E-11 | -0.112 |
| TC_ILN | SE | ENSSSCG00000007466 | SLC9A8 | 4.32E-20 | 0.34 |
| TC_ILN | SE | ENSSSCG00000012328 | HUWE1 | 0.0024 | -0.134 |
| TC_ILN | SE | ENSSSCG00000004716 | NA | 0.0011 | 0.235 |
| TC_ILN | SE | ENSSSCG00000002426 | ZC3H14 | 2.25E-09 | -0.2 |
| TC_ILN | SE | ENSSSCG00000002426 | ZC3H14 | 0.011749 | -0.257 |
| TC_ILN | SE | ENSSSCG00000002426 | ZC3H14 | 2.42E-15 | -0.388 |
| TC_ILN | SE | ENSSSCG00000003017 | TGFB1 | 0.005896 | -0.108 |
| TC_ILN | SE | ENSSSCG00000021439 | CCDC66 | 2.11E-17 | -0.121 |
| TC_ILN | SE | ENSSSCG00000021439 | CCDC66 | 2.26E-16 | -0.11 |
| TC_ILN | SE | ENSSSCG00000021899 | PIEZO2 | 0.025736 | 0.317 |
| TC_ILN | SE | ENSSSCG00000011550 | CAMK1 | 1.01E-06 | -0.229 |
| TC_ILN | SE | ENSSSCG00000013894 | IL12RB1 | 6.22E-06 | 0.167 |
| TC_ILN | SE | ENSSSCG00000000753 | WNK1 | 0.000108 | 0.18 |
| TC_ILN | SE | ENSSSCG00000005725 | TTF1 | 0.000823 | 0.11 |
| TC_ILN | SE | ENSSSCG00000005725 | TTF1 | 1.38E-09 | 0.106 |
| TC_ILN | SE | ENSSSCG00000005725 | TTF1 | 7.59E-12 | 0.293 |
| TC_ILN | SE | ENSSSCG00000014831 | PAAF1 | 6.67E-11 | 0.216 |
| TC_ILN | SE | ENSSSCG00000014831 | PAAF1 | 0.046502 | 0.172 |
| TC_ILN | SE | ENSSSCG00000004417 | NA | 0.005276 | 0.203 |
| TC_ILN | SE | ENSSSCG00000011849 | TNK2 | 0.036041 | -0.444 |
| TC_ILN | SE | ENSSSCG00000014048 | HK3 | 2.27E-85 | -0.24 |
| TC_ILN | SE | ENSSSCG00000010831 | DUSP10 | 1.88E-06 | -0.126 |
| TC_ILN | SE | ENSSSCG00000014048 | HK3 | 1.38E-21 | -0.149 |
| TC_ILN | SE | ENSSSCG00000012910 | RPS6KB2 | 1.05E-14 | -0.185 |
| TC_ILN | SE | ENSSSCG00000011182 | ASTE1 | 3.72E-32 | 0.162 |
| TC_ILN | SE | ENSSSCG00000010742 | UROS | 0.000261 | -0.11 |
| TC_ILN | SE | ENSSSCG00000015815 | FGFR1 | 0.01647 | 0.194 |
| TC_ILN | SE | ENSSSCG00000010698 | FGFR2 | 0.007457 | 0.331 |
| TC_ILN | SE | ENSSSCG00000008697 | HTT | 2.10E-34 | -0.242 |
| TC_ILN | SE | ENSSSCG00000000705 | CD27 | 5.37E-41 | -0.106 |
| TC_ILN | SE | ENSSSCG00000000705 | CD27 | 0.030428 | -0.425 |
| TC_ILN | SE | ENSSSCG00000009901 | SIRT4 | 1.62E-27 | -0.206 |
| TC_ILN | SE | ENSSSCG00000005191 | MPDZ | 1.14E-58 | -0.41 |
| TC_ILN | SE | ENSSSCG00000004163 | BCLAF1 | 1.93E-07 | -0.105 |
| TC_ILN | SE | ENSSSCG00000027331 | COL6A3 | 2.65E-30 | 0.114 |
| TC_ILN | SE | ENSSSCG00000015310 | AKAP9 | 0.008317 | -0.238 |
| TC_ILN | SE | ENSSSCG00000021814 | ZMYM2 | 0.001088 | -0.134 |
| TC_ILN | SE | ENSSSCG00000025854 | MMGT1 | 5.83E-26 | -0.109 |
| TC_ILN | SE | ENSSSCG00000013755 | CCDC130 | 1.01E-21 | -0.534 |
| TC_ILN | SE | ENSSSCG00000009978 | AP1B1 | 1.21E-11 | -0.272 |
| TC_ILN | SE | ENSSSCG00000016174 | FN1 | 2.42E-13 | -0.224 |
| TC_ILN | SE | ENSSSCG00000014060 | KIAA1191 | 4.69E-21 | -0.393 |
| TC_ILN | SE | ENSSSCG00000013322 | IMMP1L | 1.15E-24 | -0.252 |
| TC_ILN | SE | ENSSSCG00000013322 | IMMP1L | 5.16E-23 | -0.191 |
| TC_ILN | SE | ENSSSCG00000013322 | IMMP1L | 0.019936 | -0.145 |
| TC_ILN | SE | ENSSSCG00000011314 | LZTFL1 | 0.000166 | -0.158 |
| TC_ILN | SE | ENSSSCG00000028674 | CR2 | 0 | 0.25 |
| TC_ILN | SE | ENSSSCG00000028674 | CR2 | 9.05E-06 | 0.123 |
| TC_ILN | SE | ENSSSCG00000014909 | SYTL2 | 0.044544 | -0.157 |
| TC_ILN | SE | ENSSSCG00000017360 | UBTF | 8.49E-16 | -0.137 |
| TC_ILN | SE | ENSSSCG00000017360 | UBTF | 1.10E-59 | 0.156 |
| TC_ILN | SE | ENSSSCG00000008293 | DGUOK | 0.016758 | 0.295 |
| TC_ILN | SE | ENSSSCG00000017748 | NF1 | 0.00133 | -0.196 |
| TC_ILN | SE | ENSSSCG00000009583 | NA | 0.015315 | 0.141 |
| TC_ILN | SE | ENSSSCG00000021706 | KIF21B | 3.46E-07 | 0.206 |
| TC_ILN | SE | ENSSSCG00000004516 | MBD1 | 1.75E-08 | -0.127 |
| TC_ILN | SE | ENSSSCG00000006650 | GOLPH3L | 2.25E-40 | -0.124 |
| TC_ILN | SE | ENSSSCG00000030520 | NA | 4.61E-67 | -0.215 |
| TC_ILN | SE | ENSSSCG00000000968 | SBF1 | 6.15E-18 | -0.168 |
| TC_ILN | SE | ENSSSCG00000000953 | NA | 3.62E-14 | -0.351 |
| TC_ILN | SE | ENSSSCG00000007252 | DNMT3B | 0.002482 | -0.178 |
| TC_ILN | SE | ENSSSCG00000009015 | NA | 6.20E-09 | 0.196 |
| TC_ILN | SE | ENSSSCG00000021819 | NA | 9.72E-12 | -0.103 |
| TC_ILN | SE | ENSSSCG00000017694 | ACACA | 1.22E-05 | -0.11 |
| TC_ILN | SE | ENSSSCG00000010483 | PLCE1 | 9.62E-16 | -0.152 |
| TC_ILN | SE | ENSSSCG00000012123 | RAB9A | 0.017515 | -0.161 |
| TC_ILN | SE | ENSSSCG00000012123 | RAB9A | 0.049968 | -0.268 |
| TC_ILN | SE | ENSSSCG00000010476 | NA | 0.025576 | -0.371 |
| TC_ILN | SE | ENSSSCG00000005367 | TSTD2 | 0.021483 | -0.177 |
| TC_ILN | SE | ENSSSCG00000016095 | NA | 0.000256 | 0.158 |
| TC_ILN | SE | ENSSSCG00000000688 | LAG3 | 1.03E-88 | 0.26 |
| TC_ILN | SE | ENSSSCG00000015588 | ANGEL2 | 6.40E-10 | -0.159 |
| TC_ILN | SE | ENSSSCG00000013314 | QSER1 | 3.62E-07 | -0.178 |
| TC_ILN | SE | ENSSSCG00000013314 | QSER1 | 1.65E-25 | -0.15 |
| TC_ILN | SE | ENSSSCG00000005286 | CEP78 | 0.000153 | 0.369 |
| TC_ILN | SE | ENSSSCG00000013083 | CPSF7 | 1.43E-16 | -0.327 |
| TC_ILN | SE | ENSSSCG00000002368 | LTBP2 | 4.72E-09 | 0.285 |
| TC_ILN | SE | ENSSSCG00000008600 | PUM2 | 0.000261 | 0.132 |
| TC_ILN | SE | ENSSSCG00000025305 | NA | 1.51E-05 | -0.389 |
| TC_ILN | SE | ENSSSCG00000015853 | NA | 1.24E-05 | -0.146 |
| TC_ILN | SE | ENSSSCG00000000648 | CLEC7A | 0.001901 | 0.112 |
| TC_ILN | SE | ENSSSCG00000013649 | ICAM3 | 2.99E-10 | -0.114 |
| TC_ILN | SE | ENSSSCG00000000042 | NA | 4.60E-05 | -0.166 |
| TC_ILN | SE | ENSSSCG00000012703 | RBMX | 0.000345 | -0.302 |
| TC_ILN | SE | ENSSSCG00000004166 | AHI1 | 1.25E-10 | -0.13 |
| TC_ILN | SE | ENSSSCG00000004832 | UBE3A | 6.20E-37 | 0.286 |
| TC_ILN | SE | ENSSSCG00000021363 | CHD3 | 1.35E-27 | -0.174 |
| TC_ILN | SE | ENSSSCG00000012132 | ASB9 | 0.002001 | 0.312 |
| TC_ILN | SE | ENSSSCG00000024674 | ABL2 | 5.13E-08 | -0.127 |
| TC_ILN | SE | ENSSSCG00000014387 | DIAPH1 | 0.019835 | -0.186 |
| TC_ILN | SE | ENSSSCG00000006340 | UAP1 | 2.70E-49 | -0.239 |
| TC_ILN | SE | ENSSSCG00000029219 | SH3BGR | 0.001256 | 0.184 |
| TC_ILN | SE | ENSSSCG00000026264 | HSPB11 | 0.003175 | -0.112 |
| TC_ILN | SE | ENSSSCG00000023419 | NA | 8.50E-14 | 0.153 |
| TC_ILN | SE | ENSSSCG00000010401 | NA | 0.012648 | -0.108 |
| TC_ILN | SE | ENSSSCG00000005602 | GAPVD1 | 1.09E-73 | -0.253 |
| TC_ILN | SE | ENSSSCG00000015564 | TSEN15 | 9.35E-28 | -0.278 |
| TC_ILN | SE | ENSSSCG00000010952 | ZCCHC6 | 0.00633 | -0.178 |
| TC_ILN | SE | ENSSSCG00000010952 | ZCCHC6 | 2.99E-08 | -0.131 |
| TC_ILN | SE | ENSSSCG00000024006 | NA | 0.000653 | -0.119 |
| TC_ILN | SE | ENSSSCG00000012241 | BCOR | 1.06E-05 | -0.192 |
| TC_ILN | SE | ENSSSCG00000006252 | NA | 0.005578 | -0.114 |
| TC_ILN | SE | ENSSSCG00000006529 | SLC50A1 | 0.004982 | 0.247 |
| TC_ILN | SE | ENSSSCG00000006529 | SLC50A1 | 2.57E-19 | 0.11 |
| TC_ILN | SE | ENSSSCG00000006529 | SLC50A1 | 3.11E-31 | 0.18 |
| TC_ILN | SE | ENSSSCG00000027374 | C1orf50 | 1.99E-06 | 0.122 |
| TC_ILN | SE | ENSSSCG00000008845 | SRD5A3 | 0.029548 | 0.331 |
| TC_ILN | SE | ENSSSCG00000008845 | SRD5A3 | 0.000301 | -0.124 |
| TC_ILN | SE | ENSSSCG00000017100 | MTRR | 7.16E-09 | 0.323 |
| TC_ILN | SE | ENSSSCG00000012889 | CHKA | 1.50E-06 | -0.151 |
| TC_ILN | SE | ENSSSCG00000020720 | MRVI1 | 0.012636 | -0.354 |
| TC_ILN | SE | ENSSSCG00000023304 | SRRM2 | 1.52E-48 | -0.179 |
| TC_ILN | SE | ENSSSCG00000027594 | USP16 | 3.75E-06 | -0.144 |
| TC_ILN | SE | ENSSSCG00000004620 | MYO5A | 1.84E-24 | -0.275 |
| TC_ILN | SE | ENSSSCG00000014817 | ARAP1 | 0.005342 | -0.183 |
| TC_ILN | SE | ENSSSCG00000013588 | MAP2K7 | 3.13E-06 | -0.175 |
| TC_ILN | SE | ENSSSCG00000000971 | NA | 0.006915 | -0.155 |
| TC_ILN | SE | ENSSSCG00000006203 | COPS5 | 4.61E-15 | -0.153 |
| TC_ILN | SE | ENSSSCG00000014028 | CLK4 | 6.66E-14 | 0.114 |
| TC_ILN | SE | ENSSSCG00000010302 | USP54 | 0.014723 | 0.116 |
| TC_ILN | SE | ENSSSCG00000000267 | C12orf10 | 0.015028 | 0.125 |
| TC_ILN | SE | ENSSSCG00000015945 | METTL8 | 1.08E-05 | -0.142 |
| TC_ILN | SE | ENSSSCG00000012396 | MED12 | 6.72E-24 | -0.1 |
| TC_ILN | SE | ENSSSCG00000006897 | NA | 0.006104 | -0.15 |
| TC_ILN | SE | ENSSSCG00000006897 | NA | 3.82E-05 | -0.382 |
| TC_ILN | SE | ENSSSCG00000008503 | FEZ2 | 0.000307 | 0.111 |
| TC_ILN | SE | ENSSSCG00000014046 | ZNF346 | 0.020034 | -0.103 |
| TC_ILN | SE | ENSSSCG00000025731 | ALKBH3 | 1.24E-21 | -0.296 |
| TC_ILN | SE | ENSSSCG00000025731 | ALKBH3 | 0.044109 | -0.364 |
| TC_ILN | SE | ENSSSCG00000025731 | ALKBH3 | 4.28E-05 | -0.138 |
| TC_ILN | SE | ENSSSCG00000001572 | FGD2 | 1.34E-12 | -0.132 |
| TC_ILN | SE | ENSSSCG00000001572 | FGD2 | 0.000692 | -0.159 |
| TC_ILN | SE | ENSSSCG00000001572 | FGD2 | 1.63E-05 | -0.149 |
| TC_ILN | SE | ENSSSCG00000011166 | ZMYND11 | 1.68E-31 | 0.154 |
| TC_ILN | SE | ENSSSCG00000009787 | CCDC62 | 2.07E-33 | -0.364 |
| TC_ILN | SE | ENSSSCG00000022958 | ZC3H4 | 0.000206 | -0.131 |
| TC_ILN | SE | ENSSSCG00000015284 | MDM4 | 0.004614 | -0.213 |
| TC_ILN | SE | ENSSSCG00000012327 | HSD17B10 | 1.51E-05 | -0.194 |
| TC_ILN | SE | ENSSSCG00000010003 | NA | 1.50E-09 | 0.237 |
| TC_ILN | SE | ENSSSCG00000017643 | SEPT4 | 0.023147 | -0.17 |
| TC_ILN | SE | ENSSSCG00000001410 | BAG6 | 7.10E-22 | -0.11 |
| TC_ILN | SE | ENSSSCG00000021791 | SENP7 | 0.000472 | 0.166 |
| TC_ILN | SE | ENSSSCG00000021669 | NA | 1.31E-19 | -0.137 |
| TC_ILN | SE | ENSSSCG00000000519 | GLIPR1 | 8.44E-31 | -0.145 |
| TC_ILN | SE | ENSSSCG00000010351 | CCSER2 | 2.44E-25 | -0.302 |
| TC_ILN | SE | ENSSSCG00000011284 | NKTR | 0.043624 | -0.151 |
| TC_ILN | SE | ENSSSCG00000027130 | TNFRSF12A | 1.13E-05 | -0.216 |
| TC_ILN | SE | ENSSSCG00000006880 | NA | 0.038674 | 0.458 |
| TC_ILN | SE | ENSSSCG00000009620 | BMP1 | 2.49E-07 | -0.185 |
| TC_ILN | SE | ENSSSCG00000015882 | BAZ2B | 0.00105 | 0.304 |
| TC_ILN | SE | ENSSSCG00000009157 | TET2 | 0.049536 | -0.107 |
| TC_ILN | SE | ENSSSCG00000023703 | NA | 0.000156 | -0.28 |
| TC_ILN | SE | ENSSSCG00000012411 | PHKA1 | 8.56E-05 | -0.477 |
| TC_ILN | SE | ENSSSCG00000017400 | NAGLU | 0.003016 | -0.132 |
| TC_ILN | SE | ENSSSCG00000007146 | SIGLEC1 | 7.27E-11 | -0.113 |
| TC_ILN | SE | ENSSSCG00000022178 | NA | 8.87E-57 | 0.134 |
| TC_ILN | SE | ENSSSCG00000009113 | METTL14 | 0.035908 | 0.125 |
| TC_ILN | SE | ENSSSCG00000012202 | GK | 0.002749 | -0.232 |
| TC_ILN | SE | ENSSSCG00000010133 | ARVCF | 0.008027 | -0.339 |
| TC_ILN | SE | ENSSSCG00000006500 | UBQLN4 | 0.044791 | 0.11 |
| TC_ILN | SE | ENSSSCG00000024736 | ACPT | 2.89E-06 | -0.193 |
| TC_ILN | SE | ENSSSCG00000024736 | ACPT | 0.004952 | 0.15 |
| TC_ILN | SE | ENSSSCG00000024736 | ACPT | 9.96E-15 | -0.35 |
| TC_ILN | SE | ENSSSCG00000024736 | ACPT | 4.56E-13 | -0.198 |
| TC_ILN | SE | ENSSSCG00000021761 | UBAP1 | 0.008715 | 0.37 |
| TC_ILN | SE | ENSSSCG00000007336 | NNAT | 0.030195 | -0.192 |
| TC_ILN | SE | ENSSSCG00000009148 | LEF1 | 6.05E-06 | -0.183 |
| TC_ILN | SE | ENSSSCG00000009290 | NA | 0.005154 | 0.118 |
| TC_ILN | SE | ENSSSCG00000011538 | LMCD1 | 5.80E-09 | -0.116 |
| TC_ILN | SE | ENSSSCG00000010426 | PCDH15 | 6.40E-39 | -0.652 |
| TC_ILN | SE | ENSSSCG00000009138 | CFI | 0.000368 | -0.143 |
| TC_ILN | SE | ENSSSCG00000007007 | IDO1 | 6.85E-10 | 0.147 |
| TC_ILN | SE | ENSSSCG00000012944 | PELI3 | 3.34E-27 | -0.604 |
| TC_ILN | SE | ENSSSCG00000003564 | GPATCH3 | 9.87E-13 | 0.135 |
| TC_ILN | SE | ENSSSCG00000023857 | ARHGEF40 | 2.03E-32 | -0.161 |
| TC_ILN | SE | ENSSSCG00000013984 | SNAP47 | 1.17E-38 | 0.154 |
| TC_ILN | SE | ENSSSCG00000001929 | PARP6 | 1.27E-10 | -0.263 |
| TC_ILN | SE | ENSSSCG00000008556 | KHK | 4.10E-39 | 0.275 |
| TC_ILN | SE | ENSSSCG00000007309 | RBM39 | 0 | -0.194 |
| TC_Spleen | MXE | ENSSSCG00000024736 | ACPT | 1.61E-20 | 0.149 |
| TC_Spleen | MXE | ENSSSCG00000005855 | EXD3 | 4.77E-12 | -0.16 |
| TC_Spleen | MXE | ENSSSCG00000009319 | PAN3 | 4.67E-06 | -0.123 |
| TC_Spleen | MXE | ENSSSCG00000029082 | KMT2E | 6.43E-13 | -0.198 |
| TC_Spleen | MXE | ENSSSCG00000011672 | RASA2 | 0.010623 | 0.208 |
| TC_Spleen | MXE | ENSSSCG00000009924 | NA | 1.7E-10 | 0.118 |
| TC_Spleen | MXE | ENSSSCG00000002502 | ATG2B | 9.41E-14 | -0.205 |
| TC_Spleen | MXE | ENSSSCG00000011575 | ATG7 | 0.002345 | 0.169 |
| TC_Spleen | MXE | ENSSSCG00000025579 | NA | 0.015703 | 0.131 |
| TC_Spleen | MXE | ENSSSCG00000012118 | TLR8 | 2.94E-16 | -0.142 |
| TC_Spleen | MXE | ENSSSCG00000030265 | NA | 6.34E-14 | -0.102 |
| TC_Spleen | MXE | ENSSSCG00000005060 | KTN1 | 0.010956 | 0.148 |
| TC_Spleen | MXE | ENSSSCG00000029183 | SEC11A | 0.001392 | -0.109 |
| TC_Spleen | MXE | ENSSSCG00000014307 | H2AFY | 1.15E-16 | 0.127 |
| TC_Spleen | MXE | ENSSSCG00000009172 | NA | 7.35E-17 | 0.11 |
| TC_Spleen | MXE | ENSSSCG00000015820 | WHSC1L1 | 2.93E-07 | 0.16 |
| TC_Spleen | MXE | ENSSSCG00000014998 | AASDHPPT | 0.005606 | 0.113 |
| TC_Spleen | MXE | ENSSSCG00000014276 | SLC22A5 | 0.000146 | -0.159 |
| TC_Spleen | MXE | ENSSSCG00000015107 | VPS11 | 3.97E-05 | -0.15 |
| TC_Spleen | MXE | ENSSSCG00000007048 | CRLS1 | 0.022211 | -0.126 |
| TC_Spleen | MXE | ENSSSCG00000000728 | PARP11 | 6.27E-21 | 0.196 |
| TC_Spleen | MXE | ENSSSCG00000012122 | TCEANC | 1.92E-21 | 0.199 |
| TC_Spleen | MXE | ENSSSCG00000027264 | NA | 0.0002 | 0.12 |
| TC_Spleen | MXE | ENSSSCG00000014349 | TMEM173 | 2.44E-43 | -0.175 |
| TC_Spleen | MXE | ENSSSCG00000014349 | TMEM173 | 4E-146 | -0.259 |
| TC_Spleen | MXE | ENSSSCG00000014349 | TMEM173 | 7.71E-05 | -0.118 |
| TC_Spleen | MXE | ENSSSCG00000010011 | NA | 1.25E-05 | 0.102 |
| TC_Spleen | MXE | ENSSSCG00000010011 | NA | 2.75E-09 | 0.143 |
| TC_Spleen | MXE | ENSSSCG00000014239 | CEP120 | 2.3E-05 | -0.11 |
| TC_Spleen | MXE | ENSSSCG00000008448 | PREPL | 0.005144 | 0.192 |
| TC_Spleen | MXE | ENSSSCG00000007282 | PIGU | 9.45E-13 | -0.139 |
| TC_Spleen | MXE | ENSSSCG00000014868 | EMSY | 7.21E-19 | 0.18 |
| TC_Spleen | MXE | ENSSSCG00000013857 | EPS15L1 | 0.012349 | 0.341 |
| TC_Spleen | MXE | ENSSSCG00000013857 | EPS15L1 | 3.92E-10 | -0.161 |
| TC_Spleen | MXE | ENSSSCG00000025606 | NA | 7.5E-06 | 0.103 |
| TC_Spleen | MXE | ENSSSCG00000028109 | SNTB2 | 1.87E-15 | 0.114 |
| TC_Spleen | MXE | ENSSSCG00000028378 | PHF20 | 8.66E-06 | -0.141 |
| TC_Spleen | MXE | ENSSSCG00000012779 | ABCD1 | 1.3E-16 | 0.205 |
| TC_Spleen | MXE | ENSSSCG00000015878 | NA | 5.85E-22 | 0.162 |
| TC_Spleen | MXE | ENSSSCG00000010316 | KAT6B | 0.001857 | -0.238 |
| TC_Spleen | MXE | ENSSSCG00000008581 | NCOA1 | 2.52E-08 | 0.141 |
| TC_Spleen | MXE | ENSSSCG00000006165 | SEC31B | 2.16E-12 | -0.194 |
| TC_Spleen | MXE | ENSSSCG00000027765 | FAM179B | 3.11E-13 | -0.124 |
| TC_Spleen | MXE | ENSSSCG00000008221 | KDM3A | 0.0175 | 0.125 |
| TC_Spleen | MXE | ENSSSCG00000028905 | TNIK | 0.012897 | -0.112 |
| TC_Spleen | MXE | ENSSSCG00000026940 | CASP10 | 9.05E-12 | 0.152 |
| TC_Spleen | MXE | ENSSSCG00000009407 | ESD | 1E-27 | -0.105 |
| TC_Spleen | MXE | ENSSSCG00000016954 | ERBB2IP | 1.35E-24 | 0.144 |
| TC_Spleen | MXE | ENSSSCG00000009890 | MAPKAPK5 | 6.79E-15 | -0.162 |
| TC_Spleen | MXE | ENSSSCG00000016954 | ERBB2IP | 2.75E-55 | -0.177 |
| TC_Spleen | MXE | ENSSSCG00000015789 | SNX25 | 0.000546 | 0.17 |
| TC_Spleen | MXE | ENSSSCG00000015789 | SNX25 | 1.83E-09 | -0.148 |
| TC_Spleen | MXE | ENSSSCG00000015789 | SNX25 | 1.35E-13 | -0.264 |
| TC_Spleen | MXE | ENSSSCG00000015843 | UBXN8 | 8.59E-10 | 0.149 |
| TC_Spleen | MXE | ENSSSCG00000015843 | UBXN8 | 2.38E-21 | 0.112 |
| TC_Spleen | MXE | ENSSSCG00000008919 | EPHA5 | 1.45E-07 | -0.156 |
| TC_Spleen | MXE | ENSSSCG00000009759 | SCARB1 | 9.89E-06 | 0.135 |
| TC_Spleen | MXE | ENSSSCG00000015294 | CR1 | 0.000433 | 0.134 |
| TC_Spleen | MXE | ENSSSCG00000001757 | WDR61 | 3.01E-28 | -0.125 |
| TC_Spleen | MXE | ENSSSCG00000003863 | NA | 4.18E-13 | -0.173 |
| TC_Spleen | MXE | ENSSSCG00000017790 | NA | 2E-07 | 0.106 |
| TC_Spleen | MXE | ENSSSCG00000009732 | CHFR | 2.05E-59 | -0.233 |
| TC_Spleen | MXE | ENSSSCG00000021383 | MB21D1 | 4.39E-10 | 0.11 |
| TC_Spleen | MXE | ENSSSCG00000011645 | CEP63 | 1.87E-10 | -0.176 |
| TC_Spleen | MXE | ENSSSCG00000007458 | NCOA3 | 1.87E-21 | -0.115 |
| TC_Spleen | MXE | ENSSSCG00000006350 | FCGR2B | 0.000664 | -0.13 |
| TC_Spleen | MXE | ENSSSCG00000009658 | BNIP3L | 1.01E-37 | 0.113 |
| TC_Spleen | MXE | ENSSSCG00000000477 | HELB | 0.000234 | 0.119 |
| TC_Spleen | MXE | ENSSSCG00000010319 | SAMD8 | 0.009154 | 0.151 |
| TC_Spleen | MXE | ENSSSCG00000007514 | VAPB | 1.89E-56 | 0.169 |
| TC_Spleen | MXE | ENSSSCG00000020926 | SDHB | 1.47E-17 | 0.114 |
| TC_Spleen | MXE | ENSSSCG00000009834 | ATXN2 | 2.91E-09 | 0.159 |
| TC_Spleen | MXE | ENSSSCG00000012758 | NSDHL | 1.51E-29 | 0.132 |
| TC_Spleen | MXE | ENSSSCG00000006350 | FCGR2B | 0.012237 | -0.344 |
| TC_Spleen | MXE | ENSSSCG00000003708 | ANKRD29 | 1.31E-14 | 0.22 |
| TC_Spleen | MXE | ENSSSCG00000017278 | TEX2 | 0.001292 | 0.101 |
| TC_Spleen | MXE | ENSSSCG00000028117 | NA | 3.52E-11 | 0.114 |
| TC_Spleen | MXE | ENSSSCG00000025893 | ZBTB17 | 0.002557 | -0.143 |
| TC_Spleen | MXE | ENSSSCG00000002828 | LPCAT2 | 7.53E-33 | -0.17 |
| TC_Spleen | MXE | ENSSSCG00000003204 | VRK3 | 0.032813 | 0.173 |
| TC_Spleen | MXE | ENSSSCG00000015112 | HINFP | 1.39E-06 | 0.111 |
| TC_Spleen | MXE | ENSSSCG00000009623 | SLC39A14 | 0.003552 | 0.104 |
| TC_Spleen | MXE | ENSSSCG00000025272 | HEMK1 | 1.07E-05 | -0.137 |
| TC_Spleen | MXE | ENSSSCG00000002461 | BTBD7 | 5.76E-05 | -0.136 |
| TC_Spleen | MXE | ENSSSCG00000014191 | NA | 2.59E-12 | 0.238 |
| TC_Spleen | MXE | ENSSSCG00000007879 | NA | 7.25E-13 | 0.176 |
| TC_Spleen | MXE | ENSSSCG00000008515 | MEMO1 | 2.78E-20 | 0.153 |
| TC_Spleen | MXE | ENSSSCG00000022032 | NA | 0.038818 | -0.104 |
| TC_Spleen | MXE | ENSSSCG00000022032 | NA | 0.001127 | -0.141 |
| TC_Spleen | MXE | ENSSSCG00000011623 | TPRA1 | 7.74E-29 | -0.153 |
| TC_Spleen | MXE | ENSSSCG00000004432 | HDAC2 | 1.84E-05 | 0.112 |
| TC_Spleen | MXE | ENSSSCG00000015034 | SDHD | 2.05E-16 | -0.124 |
| TC_Spleen | MXE | ENSSSCG00000003976 | NFYC | 6.88E-09 | -0.133 |
| TC_Spleen | MXE | ENSSSCG00000027049 | SNAPIN | 2.49E-70 | -0.24 |
| TC_Spleen | MXE | ENSSSCG00000013426 | MUM1 | 4.56E-18 | -0.142 |
| TC_Spleen | MXE | ENSSSCG00000016496 | ADCK2 | 2.83E-06 | 0.102 |
| TC_Spleen | MXE | ENSSSCG00000008792 | N4BP2 | 1.25E-05 | -0.107 |
| TC_Spleen | MXE | ENSSSCG00000009664 | PTK2B | 0.041525 | -0.115 |
| TC_Spleen | MXE | ENSSSCG00000014060 | KIAA1191 | 0.006177 | 0.155 |
| TC_Spleen | MXE | ENSSSCG00000002436 | NA | 0.008038 | -0.11 |
| TC_Spleen | MXE | ENSSSCG00000003792 | LRRC40 | 0.001681 | -0.221 |
| TC_Spleen | MXE | ENSSSCG00000004411 | ZBTB24 | 1.66E-11 | -0.123 |
| TC_Spleen | MXE | ENSSSCG00000000657 | CLEC2D | 0 | -0.1 |
| TC_Spleen | MXE | ENSSSCG00000000657 | CLEC2D | 0 | -0.149 |
| TC_Spleen | MXE | ENSSSCG00000000657 | CLEC2D | 3.08E-09 | -0.147 |
| TC_Spleen | MXE | ENSSSCG00000008398 | PPP4R3B | 0.004846 | 0.153 |
| TC_Spleen | MXE | ENSSSCG00000013585 | EVI5L | 6.07E-09 | -0.157 |
| TC_Spleen | MXE | ENSSSCG00000006136 | RMDN1 | 0.017651 | -0.14 |
| TC_Spleen | MXE | ENSSSCG00000011878 | CASR | 3.93E-21 | -0.254 |
| TC_Spleen | MXE | ENSSSCG00000025117 | ATN1 | 1.02E-09 | 0.246 |
| TC_Spleen | MXE | ENSSSCG00000011393 | UBA7 | 0.025483 | 0.112 |
| TC_Spleen | MXE | ENSSSCG00000000955 | TBC1D22A | 0.045607 | -0.135 |
| TC_Spleen | MXE | ENSSSCG00000013124 | PATL1 | 2.24E-06 | -0.1 |
| TC_Spleen | MXE | ENSSSCG00000004388 | ARMC2 | 7.07E-07 | 0.211 |
| TC_Spleen | MXE | ENSSSCG00000000640 | NA | 1.8E-30 | 0.149 |
| TC_Spleen | MXE | ENSSSCG00000011282 | SEC22C | 6.72E-08 | -0.15 |
| TC_Spleen | MXE | ENSSSCG00000028771 | MEF2A | 6.1E-07 | 0.109 |
| TC_Spleen | MXE | ENSSSCG00000014902 | ANKRD42 | 2.83E-10 | 0.161 |
| TC_Spleen | MXE | ENSSSCG00000003438 | VPS13D | 6.61E-14 | -0.116 |
| TC_Spleen | MXE | ENSSSCG00000005062 | TMEM260 | 6.14E-15 | -0.132 |
| TC_Spleen | MXE | ENSSSCG00000005062 | TMEM260 | 1.37E-15 | 0.184 |
| TC_Spleen | MXE | ENSSSCG00000005466 | PTBP3 | 1.87E-19 | 0.101 |
| TC_Spleen | MXE | ENSSSCG00000005208 | RIC1 | 0.000184 | 0.104 |
| TC_Spleen | MXE | ENSSSCG00000002792 | HSF4 | 0.045582 | -0.262 |
| TC_Spleen | MXE | ENSSSCG00000014800 | RNF121 | 0.000726 | 0.139 |
| TC_Spleen | MXE | ENSSSCG00000015099 | BCL9L | 0.026478 | 0.234 |
| TC_Spleen | MXE | ENSSSCG00000015492 | DARS2 | 3.85E-05 | -0.354 |
| TC_Spleen | MXE | ENSSSCG00000011033 | VIM | 7.64E-05 | 0.111 |
| TC_Spleen | MXE | ENSSSCG00000015588 | ANGEL2 | 1.23E-08 | 0.111 |
| TC_Spleen | MXE | ENSSSCG00000008404 | MTIF2 | 0.046944 | 0.114 |
| TC_Spleen | MXE | ENSSSCG00000006388 | NA | 4.38E-10 | 0.109 |
| TC_Spleen | MXE | ENSSSCG00000006864 | CDC14A | 1.18E-08 | -0.17 |
| TC_Spleen | MXE | ENSSSCG00000006864 | CDC14A | 1.1E-05 | -0.135 |
| TC_Spleen | MXE | ENSSSCG00000004620 | MYO5A | 0.00026 | 0.12 |
| TC_Spleen | MXE | ENSSSCG00000012489 | TRMT2B | 5.47E-24 | 0.277 |
| TC_Spleen | MXE | ENSSSCG00000004592 | NA | 1.67E-58 | 0.116 |
| TC_Spleen | MXE | ENSSSCG00000002628 | ICK | 1.89E-15 | -0.185 |
| TC_Spleen | MXE | ENSSSCG00000006619 | SNX27 | 1.39E-08 | -0.118 |
| TC_Spleen | MXE | ENSSSCG00000029492 | TMEM218 | 0.000841 | 0.165 |
| TC_Spleen | MXE | ENSSSCG00000017298 | TANC2 | 0.037557 | -0.127 |
| TC_Spleen | MXE | ENSSSCG00000003683 | NDUFV2 | 0.028415 | -0.101 |
| TC_Spleen | MXE | ENSSSCG00000001384 | VARS2 | 5.67E-05 | 0.139 |
| TC_Spleen | MXE | ENSSSCG00000008041 | PKD1 | 0.00106 | 0.212 |
| TC_Spleen | MXE | ENSSSCG00000025856 | TMEM106A | 0.027616 | 0.14 |
| TC_Spleen | MXE | ENSSSCG00000005599 | RABEPK | 0.000668 | -0.114 |
| TC_Spleen | MXE | ENSSSCG00000009335 | B3GLCT | 4.5E-18 | 0.132 |
| TC_Spleen | MXE | ENSSSCG00000002374 | DLST | 0.002691 | -0.108 |
| TC_Spleen | A5SS | ENSSSCG00000007606 | TRRAP | 0.012816 | -0.209 |
| TC_Spleen | A5SS | ENSSSCG00000012576 | CHRDL1 | 0.028175 | -0.17 |
| TC_Spleen | A5SS | ENSSSCG00000015815 | FGFR1 | 1.52E-66 | 0.328 |
| TC_Spleen | RI | ENSSSCG00000000361 | CD63 | 1.35E-11 | -0.354 |
| TC_Spleen | RI | ENSSSCG00000030801 | GBP6 | 0.001211 | -0.103 |
| TC_Spleen | RI | ENSSSCG00000024604 | ATAT1 | 0.002189 | -0.123 |
| TC_Spleen | RI | ENSSSCG00000001407 | NCR3 | 0.001654 | -0.556 |
| TC_Spleen | SE | ENSSSCG00000016194 | USP37 | 1.46E-13 | -0.107 |
| TC_Spleen | SE | ENSSSCG00000016194 | USP37 | 1.64E-20 | -0.145 |
| TC_Spleen | SE | ENSSSCG00000006090 | MTERF3 | 0.000125 | -0.204 |
| TC_Spleen | SE | ENSSSCG00000007269 | PXMP4 | 8.80E-06 | -0.101 |
| TC_Spleen | SE | ENSSSCG00000010528 | ZFYVE27 | 3.22E-22 | -0.116 |
| TC_Spleen | SE | ENSSSCG00000022401 | AGTRAP | 5.02E-28 | -0.114 |
| TC_Spleen | SE | ENSSSCG00000025114 | NA | 3.26E-14 | 0.275 |
| TC_Spleen | SE | ENSSSCG00000014015 | C5orf45 | 0.005402 | -0.339 |
| TC_Spleen | SE | ENSSSCG00000009123 | CAMK2D | 3.38E-05 | 0.149 |
| TC_Spleen | SE | ENSSSCG00000026305 | PDCD1LG2 | 2.79E-28 | -0.194 |
| TC_Spleen | SE | ENSSSCG00000000728 | PARP11 | 1.11E-11 | 0.182 |
| TC_Spleen | SE | ENSSSCG00000007521 | NELFCD | 0.004288 | -0.293 |
| TC_Spleen | SE | ENSSSCG00000012122 | TCEANC | 8.28E-20 | 0.196 |
| TC_Spleen | SE | ENSSSCG00000012122 | TCEANC | 2.18E-22 | 0.206 |
| TC_Spleen | SE | ENSSSCG00000014349 | TMEM173 | 1.25E-10 | 0.276 |
| TC_Spleen | SE | ENSSSCG00000014349 | TMEM173 | 5.70E-12 | 0.201 |
| TC_Spleen | SE | ENSSSCG00000004558 | CSNK1G1 | 3.04E-05 | 0.103 |
| TC_Spleen | SE | ENSSSCG00000027489 | TMCO4 | 0.000107 | -0.137 |
| TC_Spleen | SE | ENSSSCG00000023557 | CCRL2 | 0.000888 | -0.107 |
| TC_Spleen | SE | ENSSSCG00000029806 | NA | 5.69E-26 | -0.101 |
| TC_Spleen | SE | ENSSSCG00000014868 | EMSY | 0.003404 | 0.195 |
| TC_Spleen | SE | ENSSSCG00000014868 | EMSY | 0.000373 | -0.12 |
| TC_Spleen | SE | ENSSSCG00000001858 | CIB1 | 5.41E-07 | -0.123 |
| TC_Spleen | SE | ENSSSCG00000011833 | DLG1 | 5.33E-14 | -0.229 |
| TC_Spleen | SE | ENSSSCG00000013857 | EPS15L1 | 0.002965 | 0.341 |
| TC_Spleen | SE | ENSSSCG00000013857 | EPS15L1 | 0.014559 | -0.125 |
| TC_Spleen | SE | ENSSSCG00000013775 | ADGRE5 | 4.21E-06 | 0.148 |
| TC_Spleen | SE | ENSSSCG00000005926 | PARP10 | 0.033215 | 0.102 |
| TC_Spleen | SE | ENSSSCG00000015883 | MARCH7 | 5.45E-36 | -0.261 |
| TC_Spleen | SE | ENSSSCG00000024628 | ALKBH8 | 0.015098 | -0.158 |
| TC_Spleen | SE | ENSSSCG00000009334 | HSPH1 | 1.08E-11 | -0.189 |
| TC_Spleen | SE | ENSSSCG00000015878 | NA | 3.19E-05 | 0.407 |
| TC_Spleen | SE | ENSSSCG00000013569 | PEX11G | 0.010471 | -0.1 |
| TC_Spleen | SE | ENSSSCG00000010316 | KAT6B | 1.28E-09 | -0.286 |
| TC_Spleen | SE | ENSSSCG00000003177 | BCL2L12 | 0.01641 | -0.154 |
| TC_Spleen | SE | ENSSSCG00000004498 | HAUS1 | 1.10E-14 | 0.125 |
| TC_Spleen | SE | ENSSSCG00000012262 | KDM6A | 4.59E-08 | -0.284 |
| TC_Spleen | SE | ENSSSCG00000012262 | KDM6A | 1.31E-16 | -0.159 |
| TC_Spleen | SE | ENSSSCG00000004718 | TTBK2 | 0.011079 | -0.12 |
| TC_Spleen | SE | ENSSSCG00000003460 | CASP9 | 0.00017 | 0.108 |
| TC_Spleen | SE | ENSSSCG00000008644 | KIDINS220 | 1.44E-22 | -0.179 |
| TC_Spleen | SE | ENSSSCG00000012689 | ZNF75D | 0.016742 | 0.26 |
| TC_Spleen | SE | ENSSSCG00000012689 | ZNF75D | 0.001013 | 0.236 |
| TC_Spleen | SE | ENSSSCG00000012689 | ZNF75D | 0.040509 | 0.157 |
| TC_Spleen | SE | ENSSSCG00000012689 | ZNF75D | 1.38E-05 | 0.114 |
| TC_Spleen | SE | ENSSSCG00000023929 | TXNRD3 | 7.27E-10 | -0.164 |
| TC_Spleen | SE | ENSSSCG00000007930 | MGRN1 | 3.60E-08 | -0.167 |
| TC_Spleen | SE | ENSSSCG00000011532 | SUMF1 | 0.000673 | -0.199 |
| TC_Spleen | SE | ENSSSCG00000024481 | NA | 0.00055 | -0.351 |
| TC_Spleen | SE | ENSSSCG00000017177 | ST6GALNAC2 | 3.28E-69 | -0.191 |
| TC_Spleen | SE | ENSSSCG00000026429 | ZFP14 | 8.98E-12 | -0.133 |
| TC_Spleen | SE | ENSSSCG00000004647 | DTWD1 | 1.86E-16 | -0.201 |
| TC_Spleen | SE | ENSSSCG00000023307 | FBXW11 | 1.48E-07 | -0.153 |
| TC_Spleen | SE | ENSSSCG00000029776 | FAM219B | 0.041843 | -0.219 |
| TC_Spleen | SE | ENSSSCG00000011689 | PLOD2 | 0.003836 | -0.204 |
| TC_Spleen | SE | ENSSSCG00000017894 | WSCD1 | 1.16E-08 | -0.208 |
| TC_Spleen | SE | ENSSSCG00000007248 | ASXL1 | 1.68E-16 | -0.159 |
| TC_Spleen | SE | ENSSSCG00000004729 | GANC | 0.003971 | -0.252 |
| TC_Spleen | SE | ENSSSCG00000015294 | CR1 | 2.23E-18 | -0.127 |
| TC_Spleen | SE | ENSSSCG00000004674 | SHF | 8.65E-06 | -0.175 |
| TC_Spleen | SE | ENSSSCG00000009090 | KIAA1109 | 1.40E-12 | -0.154 |
| TC_Spleen | SE | ENSSSCG00000016954 | ERBB2IP | 3.23E-05 | -0.161 |
| TC_Spleen | SE | ENSSSCG00000000887 | NA | 0.016632 | -0.145 |
| TC_Spleen | SE | ENSSSCG00000000887 | NA | 5.77E-05 | -0.102 |
| TC_Spleen | SE | ENSSSCG00000013788 | NA | 7.29E-46 | -0.156 |
| TC_Spleen | SE | ENSSSCG00000028293 | RCOR3 | 0.001802 | -0.149 |
| TC_Spleen | SE | ENSSSCG00000005173 | NA | 5.64E-26 | -0.128 |
| TC_Spleen | SE | ENSSSCG00000009142 | SEC24B | 0.009674 | 0.164 |
| TC_Spleen | SE | ENSSSCG00000026218 | FIP1L1 | 0.000124 | -0.123 |
| TC_Spleen | SE | ENSSSCG00000007721 | GTF2I | 0.029024 | -0.188 |
| TC_Spleen | SE | ENSSSCG00000008196 | FAM178B | 0.003399 | 0.176 |
| TC_Spleen | SE | ENSSSCG00000011950 | NXPE3 | 2.94E-05 | 0.231 |
| TC_Spleen | SE | ENSSSCG00000001991 | DHRS1 | 1.23E-29 | -0.164 |
| TC_Spleen | SE | ENSSSCG00000027157 | NA | 0.009726 | -0.296 |
| TC_Spleen | SE | ENSSSCG00000010519 | NA | 4.46E-49 | -0.356 |
| TC_Spleen | SE | ENSSSCG00000012586 | ALG13 | 9.08E-28 | 0.241 |
| TC_Spleen | SE | ENSSSCG00000001960 | EAPP | 7.63E-07 | -0.105 |
| TC_Spleen | SE | ENSSSCG00000024274 | NA | 0.044417 | -0.179 |
| TC_Spleen | SE | ENSSSCG00000002681 | HSDL1 | 0.045084 | -0.141 |
| TC_Spleen | SE | ENSSSCG00000022754 | SLC39A3 | 1.98E-26 | 0.159 |
| TC_Spleen | SE | ENSSSCG00000014149 | MEF2C | 5.40E-07 | -0.131 |
| TC_Spleen | SE | ENSSSCG00000016981 | CPEB4 | 0.013863 | -0.191 |
| TC_Spleen | SE | ENSSSCG00000007999 | NA | 3.80E-39 | -0.276 |
| TC_Spleen | SE | ENSSSCG00000024002 | NA | 0.000459 | -0.111 |
| TC_Spleen | SE | ENSSSCG00000012758 | NSDHL | 1.44E-05 | -0.149 |
| TC_Spleen | SE | ENSSSCG00000030229 | ABHD14A | 5.82E-05 | -0.12 |
| TC_Spleen | SE | ENSSSCG00000017278 | TEX2 | 4.09E-10 | 0.101 |
| TC_Spleen | SE | ENSSSCG00000015309 | MTERF1 | 0.047473 | 0.244 |
| TC_Spleen | SE | ENSSSCG00000024285 | DLG4 | 4.48E-16 | 0.324 |
| TC_Spleen | SE | ENSSSCG00000003712 | OSBPL1A | 6.08E-11 | -0.425 |
| TC_Spleen | SE | ENSSSCG00000007814 | NA | 0.030066 | -0.124 |
| TC_Spleen | SE | ENSSSCG00000014946 | NA | 0.016258 | 0.164 |
| TC_Spleen | SE | ENSSSCG00000013620 | PLPPR2 | 0.00012 | -0.301 |
| TC_Spleen | SE | ENSSSCG00000030791 | DOCK11 | 0.000222 | -0.218 |
| TC_Spleen | SE | ENSSSCG00000016041 | NA | 0.021671 | -0.178 |
| TC_Spleen | SE | ENSSSCG00000014889 | NDUFC2 | 1.62E-16 | 0.185 |
| TC_Spleen | SE | ENSSSCG00000016853 | RICTOR | 0.021018 | -0.103 |
| TC_Spleen | SE | ENSSSCG00000004281 | KCNQ5 | 8.03E-19 | 0.209 |
| TC_Spleen | SE | ENSSSCG00000004306 | ORC3 | 2.34E-09 | -0.211 |
| TC_Spleen | SE | ENSSSCG00000016339 | NA | 8.74E-06 | -0.118 |
| TC_Spleen | SE | ENSSSCG00000009244 | SEC31A | 0.003842 | 0.151 |
| TC_Spleen | SE | ENSSSCG00000011776 | YEATS2 | 0.010636 | -0.159 |
| TC_Spleen | SE | ENSSSCG00000020858 | NA | 0.008228 | 0.295 |
| TC_Spleen | SE | ENSSSCG00000025126 | LGI4 | 1.10E-08 | 0.352 |
| TC_Spleen | SE | ENSSSCG00000022906 | CH242-204P3.4 | 3.22E-05 | -0.213 |
| TC_Spleen | SE | ENSSSCG00000029125 | FAM13B | 0.023195 | -0.187 |
| TC_Spleen | SE | ENSSSCG00000029125 | FAM13B | 0.02724 | 0.133 |
| TC_Spleen | SE | ENSSSCG00000022262 | NA | 1.78E-22 | -0.113 |
| TC_Spleen | SE | ENSSSCG00000018049 | NA | 6.44E-38 | -0.183 |
| TC_Spleen | SE | ENSSSCG00000010700 | NSMCE4A | 2.27E-07 | -0.102 |
| TC_Spleen | SE | ENSSSCG00000009345 | NA | 0.000364 | -0.178 |
| TC_Spleen | SE | ENSSSCG00000007466 | SLC9A8 | 0.000229 | 0.194 |
| TC_Spleen | SE | ENSSSCG00000011379 | KLHDC8B | 5.86E-14 | -0.125 |
| TC_Spleen | SE | ENSSSCG00000015104 | TRAPPC4 | 1.25E-08 | 0.317 |
| TC_Spleen | SE | ENSSSCG00000004364 | HACE1 | 7.50E-09 | 0.247 |
| TC_Spleen | SE | ENSSSCG00000001800 | TM6SF1 | 0.0071 | -0.187 |
| TC_Spleen | SE | ENSSSCG00000008055 | C16orf59 | 4.88E-07 | 0.145 |
| TC_Spleen | SE | ENSSSCG00000002330 | PCNX | 0.016696 | 0.224 |
| TC_Spleen | SE | ENSSSCG00000016737 | PPIA | 0.000709 | -0.537 |
| TC_Spleen | SE | ENSSSCG00000011550 | CAMK1 | 0.000162 | -0.315 |
| TC_Spleen | SE | ENSSSCG00000011849 | TNK2 | 0.009039 | -0.312 |
| TC_Spleen | SE | ENSSSCG00000028461 | CH242-240D14.1 | 4.07E-06 | 0.424 |
| TC_Spleen | SE | ENSSSCG00000002844 | PHKB | 1.42E-05 | -0.256 |
| TC_Spleen | SE | ENSSSCG00000010034 | DEPDC5 | 4.97E-07 | 0.155 |
| TC_Spleen | SE | ENSSSCG00000014048 | HK3 | 0.001081 | -0.149 |
| TC_Spleen | SE | ENSSSCG00000028458 | METTL5 | 0.004189 | -0.116 |
| TC_Spleen | SE | ENSSSCG00000002741 | NA | 1.76E-09 | 0.19 |
| TC_Spleen | SE | ENSSSCG00000010698 | FGFR2 | 0.018613 | 0.444 |
| TC_Spleen | SE | ENSSSCG00000029130 | ELANE | 2.90E-12 | -0.291 |
| TC_Spleen | SE | ENSSSCG00000023408 | SAMD4A | 0.017632 | 0.122 |
| TC_Spleen | SE | ENSSSCG00000011045 | FAM188A | 0.00047 | 0.117 |
| TC_Spleen | SE | ENSSSCG00000009761 | NA | 5.29E-09 | -0.185 |
| TC_Spleen | SE | ENSSSCG00000010542 | CUTC | 1.96E-07 | -0.522 |
| TC_Spleen | SE | ENSSSCG00000013426 | MUM1 | 9.93E-18 | -0.143 |
| TC_Spleen | SE | ENSSSCG00000009901 | SIRT4 | 8.17E-48 | -0.431 |
| TC_Spleen | SE | ENSSSCG00000004163 | BCLAF1 | 0.03268 | -0.12 |
| TC_Spleen | SE | ENSSSCG00000009713 | CLCN3 | 0.005353 | -0.1 |
| TC_Spleen | SE | ENSSSCG00000027331 | COL6A3 | 1.48E-08 | 0.156 |
| TC_Spleen | SE | ENSSSCG00000010774 | ZNF511 | 1.22E-20 | -0.116 |
| TC_Spleen | SE | ENSSSCG00000012597 | PLS3 | 1.00E-06 | -0.11 |
| TC_Spleen | SE | ENSSSCG00000016174 | FN1 | 0.000712 | 0.115 |
| TC_Spleen | SE | ENSSSCG00000016174 | FN1 | 1.48E-16 | -0.111 |
| TC_Spleen | SE | ENSSSCG00000027417 | LDLRAD4 | 6.71E-10 | 0.108 |
| TC_Spleen | SE | ENSSSCG00000014060 | KIAA1191 | 0.002157 | 0.324 |
| TC_Spleen | SE | ENSSSCG00000013322 | IMMP1L | 0.002265 | 0.105 |
| TC_Spleen | SE | ENSSSCG00000028674 | CR2 | 6.71E-05 | 0.13 |
| TC_Spleen | SE | ENSSSCG00000015327 | CASD1 | 1.02E-05 | -0.12 |
| TC_Spleen | SE | ENSSSCG00000007237 | NA | 1.00E-06 | -0.155 |
| TC_Spleen | SE | ENSSSCG00000008640 | IAH1 | 4.05E-37 | -0.12 |
| TC_Spleen | SE | ENSSSCG00000026110 | SRPK2 | 0.001401 | -0.713 |
| TC_Spleen | SE | ENSSSCG00000017360 | UBTF | 5.23E-07 | -0.106 |
| TC_Spleen | SE | ENSSSCG00000017360 | UBTF | 0.041555 | 0.113 |
| TC_Spleen | SE | ENSSSCG00000008293 | DGUOK | 2.29E-09 | -0.147 |
| TC_Spleen | SE | ENSSSCG00000010055 | GGT5 | 6.82E-09 | -0.207 |
| TC_Spleen | SE | ENSSSCG00000017748 | NF1 | 8.26E-08 | -0.131 |
| TC_Spleen | SE | ENSSSCG00000001454 | SLA-DRB2 | 1.73E-07 | 0.123 |
| TC_Spleen | SE | ENSSSCG00000009151 | CYP2U1 | 0.013439 | -0.13 |
| TC_Spleen | SE | ENSSSCG00000008807 | GUF1 | 4.85E-05 | 0.156 |
| TC_Spleen | SE | ENSSSCG00000005205 | KIAA2026 | 0.005766 | -0.13 |
| TC_Spleen | SE | ENSSSCG00000000021 | ARHGAP8 | 0.00023 | -0.148 |
| TC_Spleen | SE | ENSSSCG00000000968 | SBF1 | 0.008246 | -0.119 |
| TC_Spleen | SE | ENSSSCG00000030520 | NA | 1.39E-05 | -0.119 |
| TC_Spleen | SE | ENSSSCG00000006194 | NCOA2 | 6.71E-10 | 0.123 |
| TC_Spleen | SE | ENSSSCG00000016262 | SP140 | 0.026152 | -0.104 |
| TC_Spleen | SE | ENSSSCG00000000953 | NA | 7.22E-07 | 0.229 |
| TC_Spleen | SE | ENSSSCG00000017694 | ACACA | 2.30E-07 | -0.456 |
| TC_Spleen | SE | ENSSSCG00000021675 | EMR2 | 5.34E-08 | 0.196 |
| TC_Spleen | SE | ENSSSCG00000012123 | RAB9A | 7.88E-18 | 0.212 |
| TC_Spleen | SE | ENSSSCG00000009350 | NA | 2.33E-17 | -0.118 |
| TC_Spleen | SE | ENSSSCG00000015692 | R3HDM1 | 2.00E-09 | -0.122 |
| TC_Spleen | SE | ENSSSCG00000015692 | R3HDM1 | 1.00E-07 | 0.111 |
| TC_Spleen | SE | ENSSSCG00000024315 | NA | 2.40E-05 | -0.109 |
| TC_Spleen | SE | ENSSSCG00000000640 | NA | 2.93E-86 | -0.209 |
| TC_Spleen | SE | ENSSSCG00000005367 | TSTD2 | 0.028164 | -0.217 |
| TC_Spleen | SE | ENSSSCG00000016095 | NA | 3.04E-32 | 0.24 |
| TC_Spleen | SE | ENSSSCG00000021714 | PRR3 | 0.00011 | -0.227 |
| TC_Spleen | SE | ENSSSCG00000011559 | IL17RC | 0.000362 | 0.109 |
| TC_Spleen | SE | ENSSSCG00000005286 | CEP78 | 0.001613 | -0.228 |
| TC_Spleen | SE | ENSSSCG00000000366 | DNAJC14 | 5.04E-13 | -0.103 |
| TC_Spleen | SE | ENSSSCG00000011594 | NR2C2 | 0.00095 | 0.377 |
| TC_Spleen | SE | ENSSSCG00000010539 | SLC25A28 | 0.010505 | -0.102 |
| TC_Spleen | SE | ENSSSCG00000024518 | NA | 0.000813 | -0.383 |
| TC_Spleen | SE | ENSSSCG00000015948 | DYNC1I2 | 2.70E-48 | -0.171 |
| TC_Spleen | SE | ENSSSCG00000016147 | PIKFYVE | 4.08E-08 | 0.14 |
| TC_Spleen | SE | ENSSSCG00000013633 | CARM1 | 6.27E-12 | -0.155 |
| TC_Spleen | SE | ENSSSCG00000015853 | NA | 5.76E-20 | -0.137 |
| TC_Spleen | SE | ENSSSCG00000013100 | NA | 0.039915 | -0.108 |
| TC_Spleen | SE | ENSSSCG00000000648 | CLEC7A | 2.49E-09 | -0.408 |
| TC_Spleen | SE | ENSSSCG00000000648 | CLEC7A | 0.038476 | 0.128 |
| TC_Spleen | SE | ENSSSCG00000015266 | FMO4 | 1.29E-31 | -0.114 |
| TC_Spleen | SE | ENSSSCG00000000042 | NA | 5.82E-98 | -0.205 |
| TC_Spleen | SE | ENSSSCG00000008404 | MTIF2 | 5.22E-07 | -0.208 |
| TC_Spleen | SE | ENSSSCG00000010024 | RNF185 | 3.55E-05 | -0.432 |
| TC_Spleen | SE | ENSSSCG00000005518 | NA | 0.004066 | -0.233 |
| TC_Spleen | SE | ENSSSCG00000029219 | SH3BGR | 1.59E-09 | -0.202 |
| TC_Spleen | SE | ENSSSCG00000012727 | FMR1 | 0.001234 | -0.178 |
| TC_Spleen | SE | ENSSSCG00000015492 | DARS2 | 4.14E-37 | 0.188 |
| TC_Spleen | SE | ENSSSCG00000010952 | ZCCHC6 | 0.033242 | -0.163 |
| TC_Spleen | SE | ENSSSCG00000017100 | MTRR | 3.95E-22 | 0.318 |
| TC_Spleen | SE | ENSSSCG00000012767 | HAUS7 | 0.000876 | 0.199 |
| TC_Spleen | SE | ENSSSCG00000026360 | CLGN | 0.036934 | 0.262 |
| TC_Spleen | SE | ENSSSCG00000023926 | NA | 6.18E-07 | -0.102 |
| TC_Spleen | SE | ENSSSCG00000006912 | HFM1 | 0.001346 | -0.221 |
| TC_Spleen | SE | ENSSSCG00000008845 | SRD5A3 | 0.025249 | 0.288 |
| TC_Spleen | SE | ENSSSCG00000008845 | SRD5A3 | 3.15E-12 | 0.194 |
| TC_Spleen | SE | ENSSSCG00000008845 | SRD5A3 | 3.72E-13 | 0.214 |
| TC_Spleen | SE | ENSSSCG00000013495 | ZFR2 | 0.037496 | -0.315 |
| TC_Spleen | SE | ENSSSCG00000011553 | NA | 2.98E-07 | -0.189 |
| TC_Spleen | SE | ENSSSCG00000020720 | MRVI1 | 2.66E-17 | -0.151 |
| TC_Spleen | SE | ENSSSCG00000023304 | SRRM2 | 0.001051 | -0.179 |
| TC_Spleen | SE | ENSSSCG00000012889 | CHKA | 0.001002 | -0.194 |
| TC_Spleen | SE | ENSSSCG00000004620 | MYO5A | 4.28E-10 | -0.696 |
| TC_Spleen | SE | ENSSSCG00000014817 | ARAP1 | 0.000179 | -0.274 |
| TC_Spleen | SE | ENSSSCG00000008395 | VRK2 | 0.010129 | -0.102 |
| TC_Spleen | SE | ENSSSCG00000009377 | THSD1 | 0.003588 | -0.297 |
| TC_Spleen | SE | ENSSSCG00000012489 | TRMT2B | 1.26E-12 | -0.222 |
| TC_Spleen | SE | ENSSSCG00000030182 | DEDD2 | 0.010926 | 0.111 |
| TC_Spleen | SE | ENSSSCG00000010130 | GNB1L | 0.002011 | 0.102 |
| TC_Spleen | SE | ENSSSCG00000000541 | FAR2 | 0.014423 | -0.136 |
| TC_Spleen | SE | ENSSSCG00000014291 | AFF4 | 3.11E-05 | -0.112 |
| TC_Spleen | SE | ENSSSCG00000003401 | KIF1B | 7.13E-08 | 0.207 |
| TC_Spleen | SE | ENSSSCG00000012148 | SYAP1 | 0.011131 | -0.17 |
| TC_Spleen | SE | ENSSSCG00000015945 | METTL8 | 2.64E-08 | -0.215 |
| TC_Spleen | SE | ENSSSCG00000015945 | METTL8 | 1.48E-11 | 0.402 |
| TC_Spleen | SE | ENSSSCG00000025731 | ALKBH3 | 0.016069 | 0.169 |
| TC_Spleen | SE | ENSSSCG00000001572 | FGD2 | 1.11E-31 | -0.232 |
| TC_Spleen | SE | ENSSSCG00000001572 | FGD2 | 0.035228 | -0.1 |
| TC_Spleen | SE | ENSSSCG00000001572 | FGD2 | 4.77E-15 | -0.218 |
| TC_Spleen | SE | ENSSSCG00000001572 | FGD2 | 6.16E-24 | -0.225 |
| TC_Spleen | SE | ENSSSCG00000025401 | CDK10 | 0.002638 | -0.135 |
| TC_Spleen | SE | ENSSSCG00000020967 | NA | 1.20E-50 | -0.106 |
| TC_Spleen | SE | ENSSSCG00000027557 | CDC14B | 0.006499 | -0.182 |
| TC_Spleen | SE | ENSSSCG00000008624 | NA | 6.50E-26 | -0.249 |
| TC_Spleen | SE | ENSSSCG00000026697 | UHRF1BP1L | 0.005251 | -0.166 |
| TC_Spleen | SE | ENSSSCG00000009787 | CCDC62 | 0.002669 | 0.197 |
| TC_Spleen | SE | ENSSSCG00000004704 | PPIP5K1 | 2.15E-19 | 0.413 |
| TC_Spleen | SE | ENSSSCG00000025856 | TMEM106A | 0.028785 | -0.134 |
| TC_Spleen | SE | ENSSSCG00000017643 | SEPT4 | 4.17E-18 | -0.108 |
| TC_Spleen | SE | ENSSSCG00000004146 | REPS1 | 3.21E-06 | -0.134 |
| TC_Spleen | SE | ENSSSCG00000021791 | SENP7 | 3.91E-06 | 0.141 |
| TC_Spleen | SE | ENSSSCG00000021791 | SENP7 | 0.040259 | 0.182 |
| TC_Spleen | SE | ENSSSCG00000001410 | BAG6 | 1.25E-13 | -0.143 |
| TC_Spleen | SE | ENSSSCG00000003531 | KDM1A | 9.56E-09 | 0.11 |
| TC_Spleen | SE | ENSSSCG00000013773 | ADGRL1 | 6.80E-10 | 0.594 |
| TC_Spleen | SE | ENSSSCG00000014970 | MTMR2 | 0.000318 | -0.118 |
| TC_Spleen | SE | ENSSSCG00000005604 | PBX3 | 0.002089 | -0.105 |
| TC_Spleen | SE | ENSSSCG00000009528 | NA | 0.007689 | -0.215 |
| TC_Spleen | SE | ENSSSCG00000015882 | BAZ2B | 1.27E-58 | -0.4 |
| TC_Spleen | SE | ENSSSCG00000005643 | NA | 2.75E-23 | -0.11 |
| TC_Spleen | SE | ENSSSCG00000013498 | EBI3 | 7.03E-43 | 0.129 |
| TC_Spleen | SE | ENSSSCG00000023703 | NA | 0.034766 | 0.381 |
| TC_Spleen | SE | ENSSSCG00000007146 | SIGLEC1 | 1.34E-07 | -0.166 |
| TC_Spleen | SE | ENSSSCG00000012619 | SEPT6 | 0.009058 | 0.554 |
| TC_Spleen | SE | ENSSSCG00000025792 | NA | 0.025078 | -0.121 |
| TC_Spleen | SE | ENSSSCG00000006354 | TOMM40L | 0.017788 | -0.102 |
| TC_Spleen | SE | ENSSSCG00000004986 | TRAPPC6B | 0.010344 | 0.104 |
| TC_Spleen | SE | ENSSSCG00000003100 | CCDC61 | 0.006549 | -0.108 |
| TC_Spleen | SE | ENSSSCG00000024736 | ACPT | 0.000151 | -0.237 |
| TC_Spleen | SE | ENSSSCG00000024736 | ACPT | 0.00096 | -0.14 |
| TC_Spleen | SE | ENSSSCG00000024736 | ACPT | 3.43E-07 | -0.153 |
| TC_Spleen | SE | ENSSSCG00000002266 | CHD2 | 5.59E-26 | -0.127 |
| TC_Spleen | SE | ENSSSCG00000028724 | NA | 4.36E-10 | -0.212 |
| TC_Spleen | SE | ENSSSCG00000014802 | NUMA1 | 2.00E-106 | -0.149 |
| TC_Spleen | SE | ENSSSCG00000012944 | PELI3 | 4.83E-29 | -0.495 |
| TC_Spleen | SE | ENSSSCG00000026809 | TFDP2 | 1.50E-33 | -0.145 |
| TC_Spleen | SE | ENSSSCG00000007309 | RBM39 | 0.001484 | -0.183 |
| TC_Spleen | SE | ENSSSCG00000012118 | TLR8 | 1.00E-33 | -0.15 |
| TC_Spleen | SE | ENSSSCG00000012118 | TLR8 | 1.11E-34 | -0.18 |
| TC_Spleen | SE | ENSSSCG00000005060 | KTN1 | 5.48E-16 | 0.217 |
| TC_Spleen | SE | ENSSSCG00000001700 | SLC29A1 | 2.79E-37 | -0.108 |
| TC_Spleen | SE | ENSSSCG00000011218 | SLC4A7 | 9.18E-05 | 0.103 |
| TC_Spleen | SE | ENSSSCG00000005288 | TLE4 | 2.64E-11 | -0.189 |
| TC_Spleen | SE | ENSSSCG00000006457 | FCRL3 | 5.29E-26 | -0.127 |
| TC_Spleen | SE | ENSSSCG00000010794 | PALB2 | 1.84E-40 | -0.207 |
| TC_Spleen | SE | ENSSSCG00000001474 | RXRB | 0.032088 | 0.127 |
| TC_Spleen | SE | ENSSSCG00000002755 | NFAT5 | 0.000974 | -0.253 |
| TC_Spleen | SE | ENSSSCG00000003768 | NEXN | 2.87E-23 | -0.544 |
| TC_Spleen | SE | ENSSSCG00000008877 | ETFDH | 0.043133 | -0.101 |
| TC_Spleen | SE | ENSSSCG00000000891 | NA | 0.037814 | -0.106 |
| TC_Spleen | SE | ENSSSCG00000006184 | TERF1 | 0.012828 | -0.261 |
