# Supplementary material for: Global Analysis of Alternative Splicing Difference in Peripheral Immune Organs between Tongcheng Pigs and Large White Pigs Artificially Infected with PRRSV In Vivo
Source: Biomed Res Int. 2020 Jan 30;2020:4045204. doi: 10.1155/2020/4045204 (PMC7011390; doi:10.1155/2020/4045204)
Supplement: Supplementary Materials — Table S1: PCR Primers used in the validation of alternative splicing transcripts. Table S2: differential ASE Statistics upon PRRSV infection in different groups. Table S3: information of differential ASEs upon PRRSV infection. Table S4: detailed information of enriched GO terms belonging to biological process by ASE genes. Table S5: description of KEGG pathways enrichment by ASE genes. Table S6: expression levels of splicing factors in the ILN and spleen of TC pigs and LW pigs upon PRRSV infection. Figure S1: (a) CASP10.SPLICING.fasta; (b) SIKE1.SPLICING.fasta. [file 4045204.f1.zip › TableS4.docx]

| **Table S4 Significantly enriched biological process GO terms of differential ASE genes in ILN and spleen between LW pigs and TC pigs upon PRRSV infection** | | | | | |
| --- | --- | --- | --- | --- | --- |
| Group | Category | Term | Count | % | P-value |
| LW_ILN | GOTERM_BP_DIRECT | GO:0006974~cellular response to DNA damage stimulus | 8 | 1.632653 | 0.003213 |
| LW_ILN | GOTERM_BP_DIRECT | GO:0000281~mitotic cytokinesis | 5 | 1.020408 | 0.003793 |
| LW_ILN | GOTERM_BP_DIRECT | GO:0045773~positive regulation of axon extension | 4 | 0.816327 | 0.004811 |
| LW_ILN | GOTERM_BP_DIRECT | GO:0046777~protein autophosphorylation | 9 | 1.836735 | 0.005167 |
| LW_ILN | GOTERM_BP_DIRECT | GO:0030521~androgen receptor signaling pathway | 4 | 0.816327 | 0.007614 |
| LW_ILN | GOTERM_BP_DIRECT | GO:0010606~positive regulation of cytoplasmic mRNA processing body assembly | 3 | 0.612245 | 0.008449 |
| LW_ILN | GOTERM_BP_DIRECT | GO:0034612~response to tumor necrosis factor | 3 | 0.612245 | 0.022287 |
| LW_ILN | GOTERM_BP_DIRECT | GO:0006367~transcription initiation from RNA polymerase II promoter | 4 | 0.816327 | 0.023838 |
| LW_ILN | GOTERM_BP_DIRECT | GO:2000036~regulation of stem cell population maintenance | 3 | 0.612245 | 0.028094 |
| LW_ILN | GOTERM_BP_DIRECT | GO:0000209~protein polyubiquitination | 6 | 1.22449 | 0.031522 |
| LW_ILN | GOTERM_BP_DIRECT | GO:0008360~regulation of cell shape | 7 | 1.428571 | 0.032757 |
| LW_ILN | GOTERM_BP_DIRECT | GO:0045893~positive regulation of transcription, DNA-templated | 12 | 2.44898 | 0.034114 |
| LW_ILN | GOTERM_BP_DIRECT | GO:0042787~protein ubiquitination involved in ubiquitin-dependent protein catabolic process | 7 | 1.428571 | 0.034522 |
| LW_ILN | GOTERM_BP_DIRECT | GO:0070584~mitochondrion morphogenesis | 3 | 0.612245 | 0.041262 |
| LW_ILN | GOTERM_BP_DIRECT | GO:0016579~protein deubiquitination | 5 | 1.020408 | 0.045213 |
| LW_ILN | GOTERM_BP_DIRECT | GO:2001237~negative regulation of extrinsic apoptotic signaling pathway | 4 | 0.816327 | 0.045986 |
| LW_ILN | GOTERM_BP_DIRECT | GO:0038083~peptidyl-tyrosine autophosphorylation | 4 | 0.816327 | 0.045986 |
| LW_ILN | GOTERM_BP_DIRECT | GO:0000712~resolution of meiotic recombination intermediates | 3 | 0.612245 | 0.048553 |
| LW_Spleen | GOTERM_BP_DIRECT | GO:0006974~cellular response to DNA damage stimulus | 6 | 1.546392 | 0.013763 |
| LW_Spleen | GOTERM_BP_DIRECT | GO:2000785~regulation of autophagosome assembly | 3 | 0.773196 | 0.02729 |
| LW_Spleen | GOTERM_BP_DIRECT | GO:0001666~response to hypoxia | 5 | 1.28866 | 0.032073 |
| LW_Spleen | GOTERM_BP_DIRECT | GO:0000209~protein polyubiquitination | 5 | 1.28866 | 0.040032 |
| LW_Spleen | GOTERM_BP_DIRECT | GO:0043552~positive regulation of phosphatidylinositol 3-kinase activity | 3 | 0.773196 | 0.041592 |
| LW_Spleen | GOTERM_BP_DIRECT | GO:0003084~positive regulation of systemic arterial blood pressure | 2 | 0.515464 | 0.043329 |
| LW_Spleen | GOTERM_BP_DIRECT | GO:0032092~positive regulation of protein binding | 4 | 1.030928 | 0.043612 |
| TC_ILN | GOTERM_BP_DIRECT | GO:0006974~cellular response to DNA damage stimulus | 8 | 1.489758 | 0.006937 |
| TC_ILN | GOTERM_BP_DIRECT | GO:0045773~positive regulation of axon extension | 4 | 0.744879 | 0.007147 |
| TC_ILN | GOTERM_BP_DIRECT | GO:0016192~vesicle-mediated transport | 8 | 1.489758 | 0.007536 |
| TC_ILN | GOTERM_BP_DIRECT | GO:0045944~positive regulation of transcription from RNA polymerase II promoter | 26 | 4.841713 | 0.009134 |
| TC_ILN | GOTERM_BP_DIRECT | GO:0006886~intracellular protein transport | 12 | 2.234637 | 0.01364 |
| TC_ILN | GOTERM_BP_DIRECT | GO:0030100~regulation of endocytosis | 4 | 0.744879 | 0.016423 |
| TC_ILN | GOTERM_BP_DIRECT | GO:0072659~protein localization to plasma membrane | 5 | 0.931099 | 0.026088 |
| TC_ILN | GOTERM_BP_DIRECT | GO:0007420~brain development | 6 | 1.117318 | 0.028531 |
| TC_ILN | GOTERM_BP_DIRECT | GO:1902902~negative regulation of autophagosome assembly | 3 | 0.558659 | 0.029073 |
| TC_ILN | GOTERM_BP_DIRECT | GO:0048568~embryonic organ development | 3 | 0.558659 | 0.029073 |
| TC_ILN | GOTERM_BP_DIRECT | GO:0048490~anterograde synaptic vesicle transport | 3 | 0.558659 | 0.044646 |
| TC_ILN | GOTERM_BP_DIRECT | GO:0042073~intraciliary transport | 3 | 0.558659 | 0.044646 |
| TC_ILN | GOTERM_BP_DIRECT | GO:0043491~protein kinase B signaling | 4 | 0.744879 | 0.048481 |
| TC_Spleen | GOTERM_BP_DIRECT | GO:0018105~peptidyl-serine phosphorylation | 8 | 2.439024 | 8.81E-04 |
| TC_Spleen | GOTERM_BP_DIRECT | GO:0045944~positive regulation of transcription from RNA polymerase II promoter | 18 | 5.487805 | 0.004165 |
| TC_Spleen | GOTERM_BP_DIRECT | GO:0008360~regulation of cell shape | 7 | 2.134146 | 0.004194 |
| TC_Spleen | GOTERM_BP_DIRECT | GO:2001022~positive regulation of response to DNA damage stimulus | 3 | 0.914634 | 0.005172 |
| TC_Spleen | GOTERM_BP_DIRECT | GO:0006974~cellular response to DNA damage stimulus | 6 | 1.829268 | 0.007902 |
| TC_Spleen | GOTERM_BP_DIRECT | GO:0030522~intracellular receptor signaling pathway | 3 | 0.914634 | 0.011953 |
| TC_Spleen | GOTERM_BP_DIRECT | GO:0048666~neuron development | 4 | 1.219512 | 0.015814 |
| TC_Spleen | GOTERM_BP_DIRECT | GO:0000122~negative regulation of transcription from RNA polymerase II promoter | 13 | 3.963415 | 0.017538 |
| TC_Spleen | GOTERM_BP_DIRECT | GO:0006351~transcription, DNA-templated | 12 | 3.658537 | 0.023402 |
| TC_Spleen | GOTERM_BP_DIRECT | GO:0002931~response to ischemia | 3 | 0.914634 | 0.024633 |
| TC_Spleen | GOTERM_BP_DIRECT | GO:0060045~positive regulation of cardiac muscle cell proliferation | 3 | 0.914634 | 0.028383 |
| TC_Spleen | GOTERM_BP_DIRECT | GO:1903146~regulation of mitophagy | 3 | 0.914634 | 0.032344 |
| TC_Spleen | GOTERM_BP_DIRECT | GO:0010977~negative regulation of neuron projection development | 3 | 0.914634 | 0.036508 |
| TC_Spleen | GOTERM_BP_DIRECT | GO:0032288~myelin assembly | 2 | 0.609756 | 0.037815 |
| TC_Spleen | GOTERM_BP_DIRECT | GO:1903753~negative regulation of p38MAPK cascade | 2 | 0.609756 | 0.037815 |
| TC_Spleen | GOTERM_BP_DIRECT | GO:0051225~spindle assembly | 3 | 0.914634 | 0.040865 |
| TC_Spleen | GOTERM_BP_DIRECT | GO:0051297~centrosome organization | 3 | 0.914634 | 0.040865 |
