# Supplementary material for: Global Analysis of Alternative Splicing Difference in Peripheral Immune Organs between Tongcheng Pigs and Large White Pigs Artificially Infected with PRRSV In Vivo
Source: Biomed Res Int. 2020 Jan 30;2020:4045204. doi: 10.1155/2020/4045204 (PMC7011390; doi:10.1155/2020/4045204)
Supplement: Supplementary Materials — Table S1: PCR Primers used in the validation of alternative splicing transcripts. Table S2: differential ASE Statistics upon PRRSV infection in different groups. Table S3: information of differential ASEs upon PRRSV infection. Table S4: detailed information of enriched GO terms belonging to biological process by ASE genes. Table S5: description of KEGG pathways enrichment by ASE genes. Table S6: expression levels of splicing factors in the ILN and spleen of TC pigs and LW pigs upon PRRSV infection. Figure S1: (a) CASP10.SPLICING.fasta; (b) SIKE1.SPLICING.fasta. [file 4045204.f1.zip › TableS5.docx]

| **Table S5 Significantly enriched KEGG pathways of differential ASE genes in ILN and spleen between LW pigs and TC pigs upon PRRSV infection** | | | | | |
| --- | --- | --- | --- | --- | --- |
| Group | Category | Term | Count | % | *P*-value |
| LW_ILN | KEGG_PATHWAY | ssc04660:T cell receptor signaling pathway | 8 | 1.632653 | 0.014885 |
| LW_ILN | KEGG_PATHWAY | ssc04141:Protein processing in endoplasmic reticulum | 10 | 2.040816 | 0.01577 |
| LW_ILN | KEGG_PATHWAY | ssc04668:TNF signaling pathway | 8 | 1.632653 | 0.016357 |
| LW_ILN | KEGG_PATHWAY | ssc04010:MAPK signaling pathway | 13 | 2.653061 | 0.017742 |
| LW_ILN | KEGG_PATHWAY | ssc04014:Ras signaling pathway | 12 | 2.44898 | 0.018825 |
| LW_ILN | KEGG_PATHWAY | ssc04622:RIG-I-like receptor signaling pathway | 6 | 1.22449 | 0.024968 |
| LW_ILN | KEGG_PATHWAY | ssc04210:Apoptosis | 6 | 1.22449 | 0.026404 |
| LW_ILN | KEGG_PATHWAY | ssc04920:Adipocytokine signaling pathway | 6 | 1.22449 | 0.029431 |
| LW_ILN | KEGG_PATHWAY | ssc05221:Acute myeloid leukemia | 5 | 1.020408 | 0.042452 |
| LW_ILN | KEGG_PATHWAY | ssc04666:Fc gamma R-mediated phagocytosis | 6 | 1.22449 | 0.04993 |
| LW_ILN | KEGG_PATHWAY | ssc04146:Peroxisome | 6 | 1.22449 | 0.04993 |
| LW_Spleen | KEGG_PATHWAY | ssc04146:Peroxisome | 7 | 1.804124 | 0.005296 |
| LW_Spleen | KEGG_PATHWAY | ssc04066:HIF-1 signaling pathway | 6 | 1.546392 | 0.04805 |
| TC_ILN | KEGG_PATHWAY | ssc04144:Endocytosis | 17 | 3.165736 | 0.002245 |
| TC_ILN | KEGG_PATHWAY | ssc04012:ErbB signaling pathway | 9 | 1.675978 | 0.002836 |
| TC_ILN | KEGG_PATHWAY | ssc04660:T cell receptor signaling pathway | 9 | 1.675978 | 0.010698 |
| TC_ILN | KEGG_PATHWAY | ssc04910:Insulin signaling pathway | 10 | 1.862197 | 0.012506 |
| TC_ILN | KEGG_PATHWAY | ssc05145:Toxoplasmosis | 9 | 1.675978 | 0.016106 |
| TC_ILN | KEGG_PATHWAY | ssc05169:Epstein-Barr virus infection | 12 | 2.234637 | 0.0187 |
| TC_ILN | KEGG_PATHWAY | ssc04620:Toll-like receptor signaling pathway | 8 | 1.489758 | 0.0252 |
| TC_ILN | KEGG_PATHWAY | ssc05215:Prostate cancer | 7 | 1.303538 | 0.034284 |
| TC_ILN | KEGG_PATHWAY | ssc05210:Colorectal cancer | 6 | 1.117318 | 0.044392 |
| TC_ILN | KEGG_PATHWAY | ssc01130:Biosynthesis of antibiotics | 11 | 2.048417 | 0.046877 |
| TC_ILN | KEGG_PATHWAY | ssc04510:Focal adhesion | 11 | 2.048417 | 0.049879 |
| TC_Spleen | KEGG_PATHWAY | ssc04919:Thyroid hormone signaling pathway | 6 | 1.829268 | 0.041019 |
| TC_Spleen | KEGG_PATHWAY | ssc05202:Transcriptional misregulation in cancer | 7 | 2.134146 | 0.044546 |
