# Supplementary material for: Global Analysis of Alternative Splicing Difference in Peripheral Immune Organs between Tongcheng Pigs and Large White Pigs Artificially Infected with PRRSV In Vivo
Source: Biomed Res Int. 2020 Jan 30;2020:4045204. doi: 10.1155/2020/4045204 (PMC7011390; doi:10.1155/2020/4045204)
Supplement: Supplementary Materials — Table S1: PCR Primers used in the validation of alternative splicing transcripts. Table S2: differential ASE Statistics upon PRRSV infection in different groups. Table S3: information of differential ASEs upon PRRSV infection. Table S4: detailed information of enriched GO terms belonging to biological process by ASE genes. Table S5: description of KEGG pathways enrichment by ASE genes. Table S6: expression levels of splicing factors in the ILN and spleen of TC pigs and LW pigs upon PRRSV infection. Figure S1: (a) CASP10.SPLICING.fasta; (b) SIKE1.SPLICING.fasta. [file 4045204.f1.zip › TableS6.docx]

| **Table S6 Expression levels of splicing factors in ILN and Spleen of TC pigs and LW pigs upon PRRSV infection** | | | | | | | | | |
| --- | --- | --- | --- | --- | --- | --- | --- | --- | --- |
| Gene_id | Official Symbol | LW_ILN_FC | LW_ILN_P | TC_ILN_FC | TC_ILN_P | LW_SPLEEN_FC | LW_SPLEEN_P | TC_SPLEEN_FC | TC_SPLEEN_P |
| ENSSSCG00000009906 | SRSF9 | 1.603788 | 0.003543 | 2.180614 | 0.044479 | 1.391741 | 0.148071 | 1.429228 | 0.062037 |
| ENSSSCG00000001564 | -- | 1.522408 | 0.159485 | 1.635726 | 0.039857 | 1.305617 | 0.342241 | 1.087171 | 0.424845 |
| ENSSSCG00000002312 | SRSF5 | 0.802665 | 0.399706 | 0.877049 | 0.62631 | 0.850418 | 0.264427 | 0.695116 | 0.006602 |
| ENSSSCG00000003791 | SRSF11 | 0.841609 | 0.275665 | 0.723352 | 0.258261 | 0.930785 | 0.459906 | 0.889646 | 0.272792 |
| ENSSSCG00000010002 | -- | 1.200373 | 0.152483 | 0.918215 | 0.707606 | 0.840186 | 0.255209 | 1.174543 | 0.57687 |
| ENSSSCG00000004313 | SRSF12 | 0.663473 | 0.214021 | 0.872955 | 0.803798 | 0.722872 | 0.254941 | 1.312832 | 0.368717 |
| ENSSSCG00000023148 | SRSF2 | 1.113138 | 0.454553 | 1.16191 | 0.30682 | 1.026458 | 0.85394 | 1.169558 | 0.150272 |
| ENSSSCG00000006665 | SF3B4 | 1.637211 | 0.019505 | 1.77799 | 0.022653 | 1.417865 | 0.013768 | 1.362163 | 0.077354 |
| ENSSSCG00000008484 | SRSF7 | 1.496223 | 0.079049 | 1.71209 | 0.075617 | 1.539609 | 0.167654 | 1.373865 | 0.051421 |
| ENSSSCG00000009744 | SFSWAP | 1.059291 | 0.163111 | 1.142751 | 0.004262 | 1.011661 | 0.855393 | 1.235953 | 0.049397 |
| ENSSSCG00000013021 | SF1 | 1.394269 | 0.103843 | 0.780621 | 0.532664 | 1.34201 | 0.112545 | 1.288069 | 0.26543 |
| ENSSSCG00000016075 | SF3B1 | 0.692179 | 0.054266 | 0.674749 | 0.223969 | 0.851874 | 0.081764 | 0.889208 | 0.386637 |
| ENSSSCG00000003643 | SF3A3 | 1.651402 | 0.05077 | 2.089774 | 0.053874 | 1.281787 | 0.273612 | 1.209418 | 0.219347 |
| ENSSSCG00000028288 | PRPF38A | 1.470824 | 0.197388 | 2.332187 | 0.037299 | 1.181538 | 0.373091 | 1.121195 | 0.456924 |
| ENSSSCG00000029341 | -- | 1.64872 | 0.013328 | 1.802705 | 0.069017 | 1.423651 | 0.126092 | 1.419329 | 0.024988 |
| ENSSSCG00000021140 | -- | 1.49017 | 0.017094 | 1.273055 | 0.236821 | 1.239989 | 0.202179 | 1.365716 | 0.007782 |
| ENSSSCG00000025323 | -- | 1.485475 | 0.04262 | 1.658982 | 0.022328 | 1.316779 | 0.132046 | 1.24247 | 0.084964 |
| ENSSSCG00000026354 | - | 0.895861 | 0.450525 | 0.688175 | 0.185905 | 1.01388 | 0.853849 | 0.883508 | 0.178319 |
| ENSSSCG00000007362 | SRSF6 | 1.302974 | 0.176355 | 1.274906 | 0.201395 | 1.238435 | 0.208527 | 1.318762 | 0.265147 |
| ENSSSCG00000002725 | SF3B3 | 1.388274 | 0.176374 | 1.919395 | 0.032173 | 1.334357 | 0.327426 | 1.393054 | 0.046249 |
| ENSSSCG00000002896 | -- | 1.746986 | 0.16474 | 1.968427 | 0.148943 | 1.532635 | 0.224191 | 1.305949 | 0.335973 |
| ENSSSCG00000003588 | SRSF4 | 1.288739 | 0.008108 | 0.762081 | 0.32892 | 1.273701 | 0.096888 | 1.53552 | 0.157776 |
| ENSSSCG00000021195 | SYF2 | 1.252114 | 0.341923 | 1.463974 | 0.068808 | 1.371761 | 0.360097 | 1.056086 | 0.821851 |
| ENSSSCG00000028588 | -- | 1.67656 | 0.002844 | 1.371266 | 0.10771 | 1.2582 | 0.155488 | 1.095902 | 0.654247 |
| ENSSSCG00000002748 | DHX38 | 1.505614 | 0.006781 | 1.927685 | 0.007181 | 1.311038 | 0.035808 | 1.424553 | 0.009495 |
| ENSSSCG00000017626 | SRSF1 | 1.527167 | 0.142673 | 1.420987 | 0.066393 | 1.426367 | 0.238166 | 1.203131 | 0.021962 |
| ENSSSCG00000021615 | SRSF10 | 1.193581 | 0.481646 | 1.336756 | 0.109354 | 1.181948 | 0.496229 | 1.058941 | 0.524903 |
| ENSSSCG00000017033 | SLU7 | 1.005696 | 0.963159 | 0.726605 | 0.297774 | 1.151724 | 0.376772 | 0.782788 | 0.105782 |
| ENSSSCG00000012957 | SF3B2 | 1.676518 | 0.01232 | 1.436047 | 0.00181 | 1.251992 | 0.000635 | 1.314787 | 0.089159 |
| ENSSSCG00000021061 | -- | 1.226219 | 0.270356 | 1.732236 | 0.086796 | 1.252689 | 0.244855 | 1.214672 | 0.024665 |
| ENSSSCG00000003182 | SCAF1 | 1.720694 | 0.193604 | 1.157517 | 0.447492 | 1.202433 | 0.460553 | 1.590791 | 0.111687 |
| ENSSSCG00000028133 | PUF60 | 1.270744 | 0.127912 | 1.962316 | 0.033582 | 1.036492 | 0.698936 | 1.412745 | 0.025749 |
| ENSSSCG00000008756 | DHX15 | 1.324677 | 0.308474 | 1.303354 | 0.039347 | 1.356536 | 0.265984 | 1.020821 | 0.705503 |
| ENSSSCG00000016008 | CWC22 | 1.024323 | 0.928101 | 0.836917 | 0.582835 | 1.2556 | 0.373225 | 0.970157 | 0.753582 |
| ENSSSCG00000007315 | AAR2 | 1.459096 | 0.023881 | 1.68999 | 0.044118 | 1.15893 | 0.118396 | 1.354972 | 0.08156 |
| ENSSSCG00000011134 | RBM17 | 1.136749 | 0.010766 | 1.675761 | 0.042455 | 1.129328 | 0.201812 | 1.392308 | 0.013761 |
| ENSSSCG00000017817 | PRPF8 | 0.974065 | 0.687305 | 0.928631 | 0.574943 | 0.943149 | 0.507019 | 1.01078 | 0.964283 |
| ENSSSCG00000006845 | PRPF38B | 0.974702 | 0.862746 | 0.843938 | 0.311402 | 1.053879 | 0.516648 | 0.960211 | 0.758521 |
| ENSSSCG00000010744 | DHX32 | 1.186392 | 0.104542 | 1.298819 | 0.092497 | 1.208121 | 0.094536 | 1.142997 | 0.291495 |
| ENSSSCG00000029308 | -- | 0.803535 | 0.786007 | 0.864919 | 0.144374 | 0.269994 | 0.015307 | 1.766324 | 0.126217 |
| ENSSSCG00000021023 | SCAF4 | 1.26997 | 0.034411 | 0.812568 | 0.475602 | 1.069632 | 0.744235 | 1.115988 | 0.690123 |
| ENSSSCG00000021478 | CWC25 | 1.343641 | 0.055278 | 1.104213 | 0.336324 | 1.236168 | 0.089461 | 1.082451 | 0.578024 |
| ENSSSCG00000023288 | -- | 0.813408 | 0.057258 | 0.49723 | 0.137095 | 0.856922 | 0.124701 | 0.991954 | 0.959489 |
| ENSSSCG00000013582 | XAB2 | 1.667126 | 0.05169 | 2.008309 | 0.044727 | 1.281334 | 0.012179 | 1.43409 | 0.024248 |
| ENSSSCG00000030507 | SMNDC1 | 1.348861 | 0.145785 | 1.133835 | 0.377433 | 1.299999 | 0.196483 | 0.98115 | 0.777729 |
| ENSSSCG00000001376 | DHX16 | 1.352073 | 0.08746 | 1.344651 | 0.074723 | 1.223583 | 0.002056 | 1.49598 | 0.023252 |
| ENSSSCG00000016572 | TNPO3 | 1.209552 | 0.338756 | 1.33063 | 0.019752 | 1.124666 | 0.547408 | 1.097644 | 0.254682 |
| ENSSSCG00000023231 | RBM22 | 1.366793 | 0.131569 | 1.478003 | 0.129922 | 1.035546 | 0.543163 | 1.284425 | 0.078979 |
| ENSSSCG00000010203 | HNRNPF | 1.407537 | 0.129581 | 1.555591 | 0.035296 | 1.298548 | 0.251217 | 1.222159 | 0.002681 |
| ENSSSCG00000021128 | HNRNPH2 | 1.520066 | 0.106319 | 1.631984 | 0.090052 | 1.357478 | 0.291224 | 1.244038 | 0.158837 |
| ENSSSCG00000009247 | -- | 0.75786 | 0.154205 | 0.762118 | 0.293764 | 1.003102 | 0.984893 | 0.879484 | 0.48281 |
| ENSSSCG00000015988 | HNRNPA3 | 0.973621 | 0.854995 | 0.654776 | 0.222904 | 0.927797 | 0.564346 | 0.926124 | 0.423594 |
| ENSSSCG00000020688 | HNRNPH3 | 1.447567 | 0.003919 | 1.006606 | 0.938132 | 1.070665 | 0.457352 | 1.019265 | 0.687447 |
| ENSSSCG00000023761 | -- | 1.166276 | 0.498485 | 1.330119 | 0.129428 | 1.044498 | 0.831732 | 1.000985 | 0.987725 |
| ENSSSCG00000009249 | HNRNPD | 1.070131 | 0.525178 | 0.749694 | 0.421929 | 0.918295 | 0.639735 | 0.988057 | 0.966352 |
| ENSSSCG00000014322 | HNRNPA0 | 2.334753 | 0.041547 | 1.778852 | 0.019868 | 0.994515 | 0.96269 | 1.533864 | 0.171289 |
| ENSSSCG00000028640 | HNRNPC | 1.636864 | 0.086877 | 1.975237 | 0.071577 | 1.428176 | 0.243037 | 1.111143 | 0.617891 |
| ENSSSCG00000015836 | HNRNPA2B1 | 1.700465 | 0.005284 | 1.040276 | 0.863089 | 1.518342 | 0.040003 | 1.352786 | 0.005001 |
| ENSSSCG00000010238 | -- | 1.02547 | 0.766327 | 0.615622 | 0.195503 | 1.048312 | 0.607805 | 0.902005 | 0.509312 |
| ENSSSCG00000013607 | -- | 0 | 0.373901 | 0 | 0.373901 | -- | 0.373901 | -- | 0.373901 |
| ENSSSCG00000028563 | HNRNPR | 1.199302 | 0.378594 | 1.071727 | 0.680557 | 1.219832 | 0.151127 | 0.957417 | 0.619784 |
| ENSSSCG00000028617 | -- | -- | -- | 0 | 0.241385 | -- | -- | -- | -- |
| ENSSSCG00000024580 | -- | 2.321847 | 0.487199 | -- | -- | 0.967424 | 0.982443 | -- | -- |
| ENSSSCG00000010877 | HNRNPU | 1.556055 | 0.005682 | 1.275697 | 0.021341 | 1.322622 | 0.039185 | 1.194481 | 0.052127 |
| ENSSSCG00000028542 | -- | 1.256993 | 0.053035 | 1.107385 | 0.298027 | 1.103502 | 0.093792 | 1.033334 | 0.84767 |
| ENSSSCG00000014031 | HNRNPAB | 2.257806 | 0.000348 | 2.397894 | 0.00316 | 1.339381 | 0.189642 | 1.695629 | 0.035421 |
| ENSSSCG00000022449 | -- | -- | -- | -- | -- | -- | -- | -- | -- |
| ENSSSCG00000013602 | HNRNPM | 1.750143 | 0.005181 | 1.656534 | 0.025601 | 1.503277 | 0.097852 | 1.259132 | 0.149626 |
| ENSSSCG00000014022 | HNRNPH1 | 1.068728 | 0.511914 | 0.895674 | 0.473997 | 1.07862 | 0.307594 | 0.938737 | 0.302524 |
| ENSSSCG00000020878 | HNRNPLL | 0.571469 | 0.000401 | 0.604878 | 0.033377 | 0.601455 | 0.013974 | 0.596636 | 0.012803 |
| ENSSSCG00000000288 | HNRNPA1 | 1.311335 | 0.027582 | 1.293888 | 0.325308 | 1.095831 | 0.465675 | 1.073459 | 0.617621 |
| ENSSSCG00000030049 | HNRNPUL1 | 1.184199 | 0.060696 | 1.285508 | 0.017862 | 0.968005 | 0.47862 | 1.010049 | 0.933732 |
| ENSSSCG00000022760 | HNRNPK | 1.244241 | 0.300791 | 1.534825 | 0.091942 | 1.260695 | 0.324984 | 1.001428 | 0.966874 |
| ENSSSCG00000007275 | RALY | 1.922917 | 0.003673 | 1.899768 | 0.052193 | 1.102745 | 0.282563 | 1.232547 | 0.286546 |
| ENSSSCG00000001932 | -- | -- | 0.373901 | 0.189703 | 0.340531 | 0.780846 | 0.848222 | 0 | 0.373901 |
| ENSSSCG00000004294 | SYNCRIP | 1.218321 | 0.48513 | 1.026441 | 0.875533 | 1.204192 | 0.438943 | 0.931851 | 0.495799 |
| ENSSSCG00000028626 | -- | 1.337298 | 0.003623 | 1.451563 | 0.022882 | 1.247673 | 0.005751 | 1.257834 | 0.196708 |
| ENSSSCG00000012703 | RBMX | 183.8861 | 0.376216 | 0.212512 | 0.091177 | 0.569495 | 0.661547 | 1.629253 | 0.63812 |
|  |  |  |  |  |  |  |  |  |  |
| FC means the expression fold change value of splicing factor between the PRRSV infected and control groups | | | | | | | | | |
| P means the *P*-value of splicing factor between the PRRSV infected and control groups | | | | | | | | |  |
